# Gametocytocidal Screen Identifies Novel Chemical Classes with *Plasmodium falciparum* Transmission Blocking Activity

**DOI:** 10.1371/journal.pone.0105817

**Published:** 2014-08-26

**Authors:** Natalie G. Sanders, David J. Sullivan, Godfree Mlambo, George Dimopoulos, Abhai K. Tripathi

**Affiliations:** W. Harry Feinstone Department of Molecular Microbiology and Immunology, Johns Hopkins Bloomberg School of Public Health, Johns Hopkins University, Baltimore, Maryland, United States of America; Food and Drug Administration, United States of America

## Abstract

Discovery of transmission blocking compounds is an important intervention strategy necessary to eliminate and eradicate malaria. To date only a small number of drugs that inhibit gametocyte development and thereby transmission from the mosquito to the human host exist. This limitation is largely due to a lack of screening assays easily adaptable to high throughput because of multiple incubation steps or the requirement for high gametocytemia. Here we report the discovery of new compounds with gametocytocidal activity using a simple and robust SYBR Green I- based DNA assay. Our assay utilizes the exflagellation step in male gametocytes and a background suppressor, which masks the staining of dead cells to achieve healthy signal to noise ratio by increasing signal of viable parasites and subtracting signal from dead parasites. By determining the contribution of exflagellation to fluorescent signal and using appropriate cutoff values, we were able to screen for gametocytocidal compounds. After assay validation and optimization, we screened an FDA approved drug library of approximately 1500 compounds, as well as the 400 compound MMV malaria box and identified 44 gametocytocidal compounds with sub to low micromolar IC_50_s. Major classes of compounds with gametocytocidal activity included quaternary ammonium compounds with structural similarity to choline, acridine-like compounds similar to quinacrine and pyronaridine, as well as antidepressant, antineoplastic, and anthelminthic compounds. Top drug candidates showed near complete transmission blocking in membrane feeding assays. This assay is simple, reproducible and demonstrated robust Z-factor values at low gametocytemia levels, making it amenable to HTS for identification of novel and potent gametocytocidal compounds.

## Introduction

Malaria is a historically relentless public health problem and continues in the present day to contribute to severe morbidity and mortality worldwide, impeding development in many of the world's poorest countries. *Plasmodium falciparum* malaria is associated with the highest fatality rates, resulting in an estimated 200 million cases and more than one million deaths in 2012 [1]. Efforts to control, eliminate, and ultimately eradicate this disease have only been partially successful, with failure due in large part to the development of drug resistance in both the *Anopheles* mosquito vector, as well as the parasite [2], [3]. Sustainable interventions and control measures have also posed a challenge, and a multi-faceted strategy targeting both transmission and disease is necessary if there is any hope of controlling this devastating disease [2]–[4].

Of particular interest is the discovery of new chemical entities and classes targeting the sexual stage of the parasite, gametocytes, which are responsible for transmission back to the mosquito vector. To this end, a variety of assays have been developed, each utilizing different measures of parasite viability including alamar blue to detect metabolic activity, detection of parasite proteins such as lactate dehydrogenase, or bioluminescence of viable transgenic parasites [5]–[11]. While the reported assays are more high-throughput than the gold standard of counting Giemsa-stained blood films, they still have limitations including the requirement for transgenic parasites or multiple incubation and transfer steps.

Here we describe a simple assay using the SYBR-green I DNA probe along with a background suppressor to assay for live gametocytes. To achieve robust signal to noise ratio we use a combination of exflagellation, to increase DNA content from viable male gametocytes, and background suppressor to mask the signals from drug killed gametocytes. Incubation time after drug treatment is minimal with no transfer or centrifugation steps and can be easily adapted to higher throughput formats such as 384 or 1536-well plates. In addition, this assay does not require transgenic parasites and thus could be used to screen field isolates. After validating the assay, we screened an FDA-approved library of 1584 compounds as well as the MMV malaria box of 400 confirmed antimalarials that are active against asexual blood stages in *P. falciparum*. We report the results of both drug screens, with particular emphasis on the novel classes of active compounds identified by the assay: quaternary ammonium compounds and acridine-like compounds.

## Materials and Methods

### Ethics statement

This study was carried out in strict accordance with the recommendations in the Guide for the Care and Use of Laboratory Animals of the National Institutes of Health. Mice were only used for mosquito rearing as a blood source according to approved protocol. The protocol was approved by the Animal Care and Use Committee of the Johns Hopkins University (ACUC MO12H76).

### *P. falciparum* gametocyte cultivation

The *P. falciparum* NF54 strain was cultured according to the method described by Trager and Jenson with minor modifications. Briefly parasites were cultured using O^+^ human erythrocytes at 4% hematocrit in parasite culture medium (RPMI 1640 supplemented with 25 mM HEPES, 10 mM Glutamine, 0.074 mM hypoxanthine and 10% O^+^ human serum. Cultures were maintained under standard conditions of 37°C in a candle jar made of glass desiccators. Gametocyte cultures were initiated at 0.5% mixed stage parasitemia from low passage stock and cultures were maintained up to day 15 with daily media changes. To achieve greater level of asexual parasitemia before gametocytogenesis, hematocrit was reduced to 2% between days 3 to 6. After day 6 hematocrit was brought back to approximately 4%. To block reinvasion of remaining asexual parasites and obtain pure and near synchronous gametocytes, cultures were treated with 50 mM *N*-acetyl-D-glucosamine (NAG) for 72 hours between days 8 to 11.

### Development and optimization of gametocytocidal assay

To determine the effect of drugs on mature gametocytes we developed a SYBR Green I based DNA quantification assay. Because gametocytes do not multiply, we utilized the decrease in live gametocytes after killing combined with an increase in DNA content of male gametes after exflagellation to quantitate the effect of drugs. For determination of live gametocytes, our assay uses a background suppressor, a live-cell impermeable dye (CyQUANT direct cell proliferation assay kit, Life Technologies, Grand Island, NY, USA) which can enter dead cells and specifically blocks green fluorescence, resulting in the subtraction of SYBR Green I signal from drug killed parasites. For assay optimization mature gametocytes were enriched using Percoll density gradient centrifugation. Enriched gametocytes were plated in 96 well plates and serially diluted with uninfected 2% hematocrit erythrocytes to obtain serial gametocytemia values. Triplicate wells of each parasite dilution were either treated with 10 µM pyrvinium pamoate or 0.1% DMSO (vehicle control) for 48 hrs at 37°C in candle jar as described above. After drug exposure, 11 µl of 10x exflagellation medium (RPMI 1640 with 200 mM HEPES, 40 mM sodium bicarbonate, 100 mM glucose pH 8.0) was added and plates were incubated at room temperature for 30 min. Next 11 µl of 10x CyQUANT direct background suppressor and SYBR Green I in PBS was added and plate was incubated at room temperature for 2 hrs. After addition of detection reagents plates were protected from light. Fluorescence was then measured at excitation and emission wavelengths of 485 and 535 in a plate reader (HTS7000 Perkin Elmer). To achieve consistent reads, special care was taken not to disturb the settled layer of gametocyte infected erythrocytes for consistent readings, during addition of reagents, incubations and detection. To determine the respective contribution of background suppressor (gametocyte killing) and exflagellation (increase in DNA content), each assay was performed in parallel plates with and without exflagellation. To prevent spontaneous exflagellation, the plate without exflagellation was maintained at 37°C until fluorescence values were determined.

### Screening of JHU FDA approved compound library

The Johns Hopkins University Clinical Compound Library version 1.3 is comprised of more than 1500 drugs, which were approved by the FDA for treatments of different diseases or medical conditions. The JHU drug library is stocked in 96 well plates at 10 mM in 100% DMSO. In order to achieve dispensable concentration we diluted compounds in incomplete RPMI to new master plates at 400 µM. We dispensed 5 µl of Compound library to the 96-well plates, to a final compound concentration of 20 µM and DMSO concentration of 0.1%. Columns 1 and 12 of each plate were used as in-plate controls and contained 0.1% DMSO (negative control, 0% inhibition) and 20 µM clotrimazole (positive control, ∼70% inhibition), respectively. Ninety-five µl of day 15 pure gametocyte cultures at approximately 3–5% gametocytemia were then added to the compound containing plates at 1% hematocrit. Plates were incubated for up to 48 hrs at 37°C in microaerophilic conditions of a candle jar. After 48 hrs of drug treatment, gametogenesis was induced by adding 11 µl of 10x exflagellation media and incubation for 30 min at room temperature. Next 11 µl of 10x CyQUANT direct background suppressor and SYBR Green I (Life Technologies, Grand Island, NY, USA) in PBS was added to the plates and further incubated at room temperature for 2 hrs in the dark. Plates were then read at excitation and emission wavelengths of 485 and 535 nm respectively and raw data was transferred to Microsoft Excel. Fluorescence signals from both negative (0.1% DMSO) and positive (clotrimazole) wells were used for quality control of the assay and to determine percent inhibition by each compound. Compounds which showed values between positive and negative controls were considered primary hits. All the hits from the primary screen were retested at 10 µM for microscopic examination and at multiple concentrations for IC_50_ determinations.

### Screening of the MMV Malaria Box

The Medicines for Malaria Venture (MMV) kindly provided the Malaria Box which was comprised of 200 drug like and 200 probe like inhibitors of *P. falciparum* asexual stage. The MMV Box was supplied in 96-well plates at 10 mM stocks in 100% DMSO. We diluted the compound library by 50 fold to make master plates at 200 µM and 5 µl of each compound was dispensed into the duplicate assay plates, to achieve final concentration of 10 µM. The first and last columns on each plate were used for the negative (0.1% DMSO) and positive (10 µM pyrvinium pamoate) controls. Plate set up and detection of fluorescence was performed as described above in the method section for the FDA approved compound library. Compounds showing >50% inhibition in both replicates were considered primary hits. All the primary hits were then retested at multiple doses and IC_50_ values were determined.

### Z factor determinations

**Figure 1:**
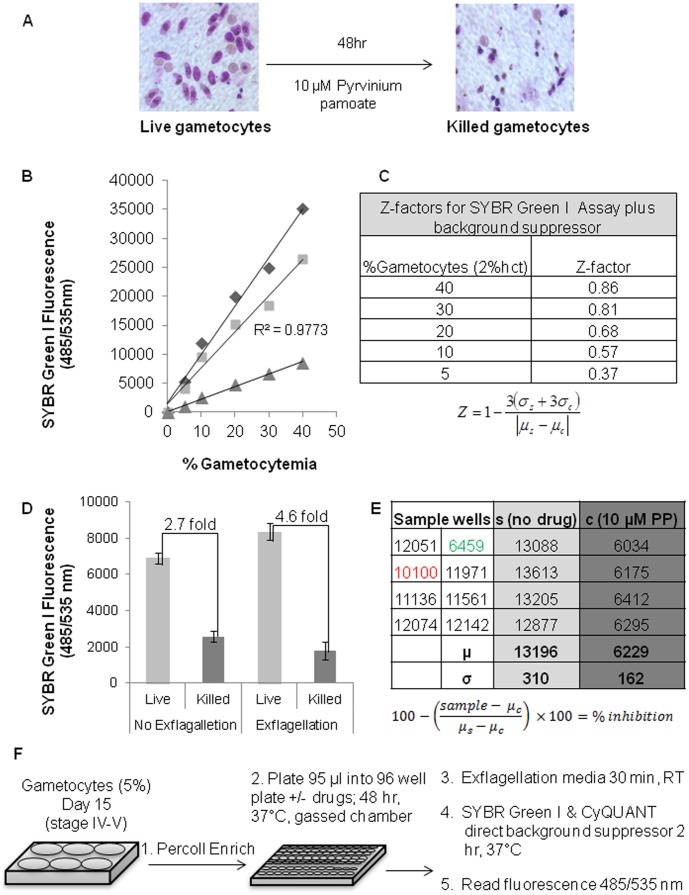
SYBR Green I-Background suppressor gametocytocidal assay. (A) Giemsa stained culture before and after treatment with 10 µM pyrvinium pamoate. (B) SYBR Green I fluorescence of gametocytes, total (diamond), killed (triangle) and live gametocytes after drug treatment (total minus killed, square) with decreasing number of gametocytes per uninfected cell, diluted with 2% hematocrit RBCs in media in presence of background suppressor. (C) Z-factors calculated for each gametocyte dilution. Z factors were calculated using the equation shown, described previously for validating high throughput assays (σ =  standard deviation, μ = mean, s = sample, c = control or in this case zero gametocytes) [10]. (D) SYBR Green I fluorescence of live or pyrvinium pamoate-killed gametocytes in the presence of CyQUANT background suppressor, with and without exflagellation with background well fluorescence (no parasites) subtracted out as a blank. (E) Example of assay plate SYBR Green I fluorescence in the presence of background suppressor and calculations for % inhibition. Green indicates a positive hit with high inhibition attributable to gametocyte killing and red indicates an intermediate hit, with inhibition attributable to potentially exflagellation inhibition and/or moderate gametocyte killing. (F) Overall assay setup with five steps: 1. Culture and enrich gametocytes, 2. Incubate with drug for 48 hr, 3. Add exflagellation media and incubate 30 min, 4. Add SYBR Green I and background suppressor and incubate 2 hr, 5. Read SYBR Green I fluorescence at excitation 485 nm and emission 535 nm.

Z factors were calculated using an equation described previously for validating high throughput assays (Figure 1C) [12]. In all assays, means and standard deviations were calculated from a minimum of four replicates for each sample well (positive pyrvinium pamoate control) and four to eight replicates for control well (negative no drug control). There were two biological replicates completed for every drug/plate (one in the second screen of the FDA drug library) and the Z-factors reported were mean Z-factors calculated from the sum of all Z-factors calculated for each assay in each screen. For example, the Z-factor for the FDA approved drug library screen was calculated as a mean of the Z-factors calculated for each assay plate (with four positive control wells and four to eight negative control wells per plate) in that screen.

### Mosquito rearing and membrane feeding assay

*Anopheles gambiae* Keele strain mosquitoes were maintained on a 10% sugar solution at 27°C and 80% humidity with a 12-h light/dark cycle according to standard rearing methods. Day 15 gametocytes were treated with gametocytocidal compounds or 0.1% DMSO for 48 hr and then were centrifuged and diluted to 0.3% final gametocytemia in a mixture of RBCs supplemented with human serum for mosquito membrane feeding assays. Unfed mosquitoes were removed after feeding, and midguts were dissected 7 days later and stained with 0.1% mercurochrome. The number of oocysts per midgut was determined with a light-contrast microscope, and the median infection intensity was calculated for the control and each experimental group.

## Results

### Assay optimization

Using SYBR Green I as a live-cell permeable fluorescent probe, we were able to detect gametocytes based on DNA content, with exflagellation as a means to increase DNA content in viable male gametes. To increase our signal to noise ratio, we used a background suppressor from the CyQUANT Direct Cell Proliferation Assay kit which works specifically by entering permeabilized or dead cells and masking green fluorescence. By using SYBR Green I in conjunction with the background suppressor, we were able to mask the signal from dead or damaged gametocytes and only read SYBR Green I fluorescence from live or intact cells. The assay was optimized to determine sensitivity comparing drug treated and untreated parasites. SYBR Green I fluorescent signal from total and killed (10 µM pyrvinium pamoate treated, Figure 1A) gametocytes was shown to increase linearly with increasing gametocytemia (Figure 1B) and after subtracting out signal from killed gametocytes, retained fluorescent signal with a coefficient of determination of 0.97, indicating strong predictive value of gametocyte number on fluorescent signal. To determine the limit of detection and sensitivity of the assay, a Z-factor was calculated for serially diluted gametocyte culture at 2% hematocrit, which showed increase in Z-factor values with increasing gametocyetmia levels (Figure 1C). Addition of the CyQUANT background suppressor dye greatly increased the sensitivity of the assay compared to exflagellation, which marginally enhanced the signal of live gametocytes (Figure 1D). Specifically, beginning with an average ratio of 4∶1 female to male mature gametocytes, exflagellation increased live gametocyte signal from 7000 to 8000 fluorescent units, suggesting a contribution of 10–20% of exflagellation to overall fluorescent signal (Figure 1D). Drugs inhibiting exflagellation but not killing the parasites would result in low to intermediate inhibition in this assay (red highlighted value, Figure 1E), with anything greater than 20% inhibition indicative of some gametocyte killing (green highlighted value, Figure 1E). Making blood films of positive hits can further differentiate whether parasites are being killed or damaged or whether exflagellation inhibition is occurring. For our assay, we set a cutoff value of greater than 70% inhibition (equal to or better than clotrimazole) for the FDA drug library screen and greater than 50% inhibition (using pyrvinium pamoate as a positive control) for the MMV box screen to capture gametocytocidal compounds. The final assay setup for drug screening is briefly illustrated in Figure 1E.

### Assay validation and FDA-approved drug library screen

**Figure 2:**
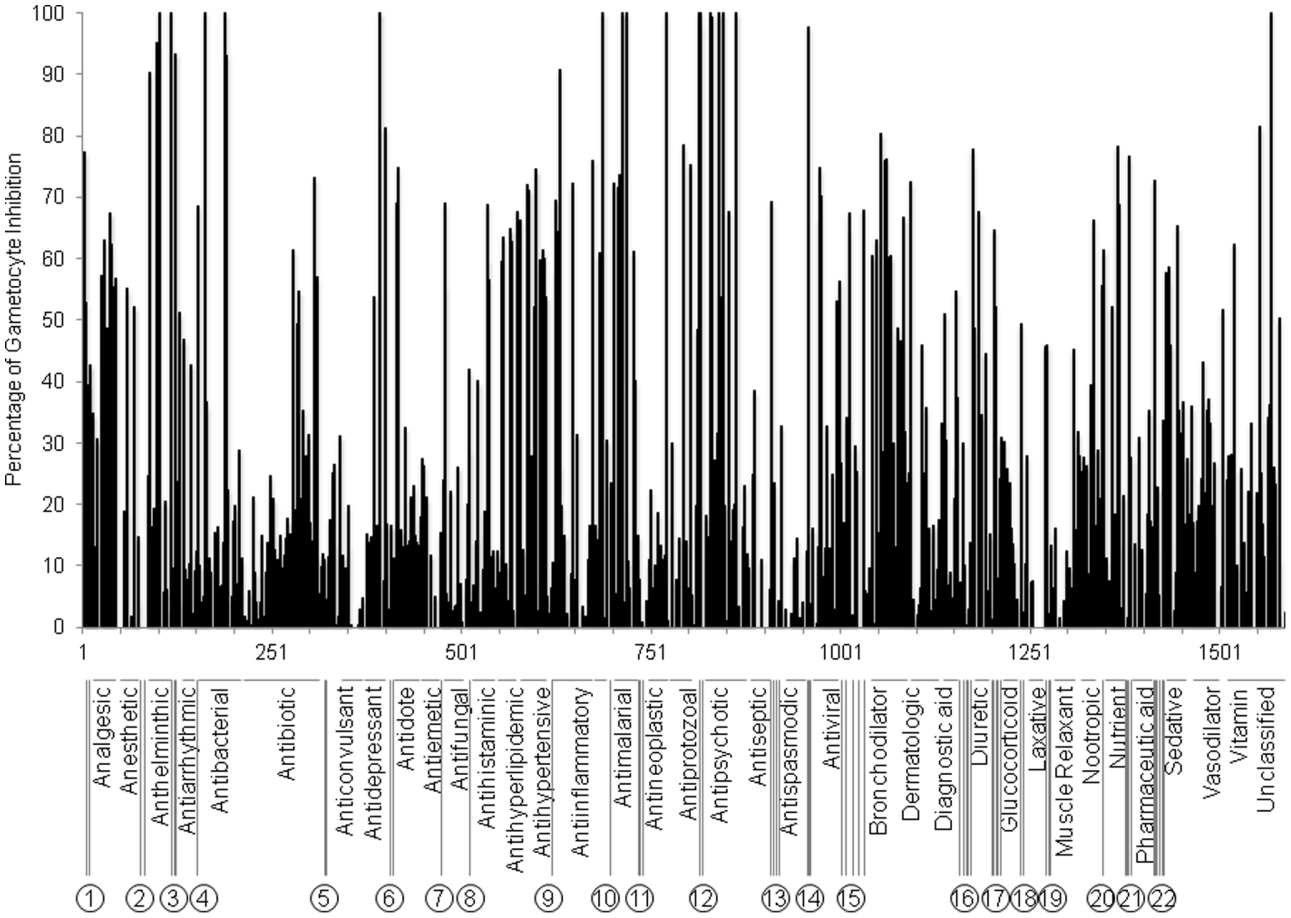
Inhibition by FDA drug library. SYBR Green I assay results for the Johns Hopkins Clinical Compound Library version 1.3 of FDA approved drugs screened at 20 µM. Plot of percentage of gametocytocidal activity of 1,584 compounds compared to clotrimazole control.

**Figure 3:**
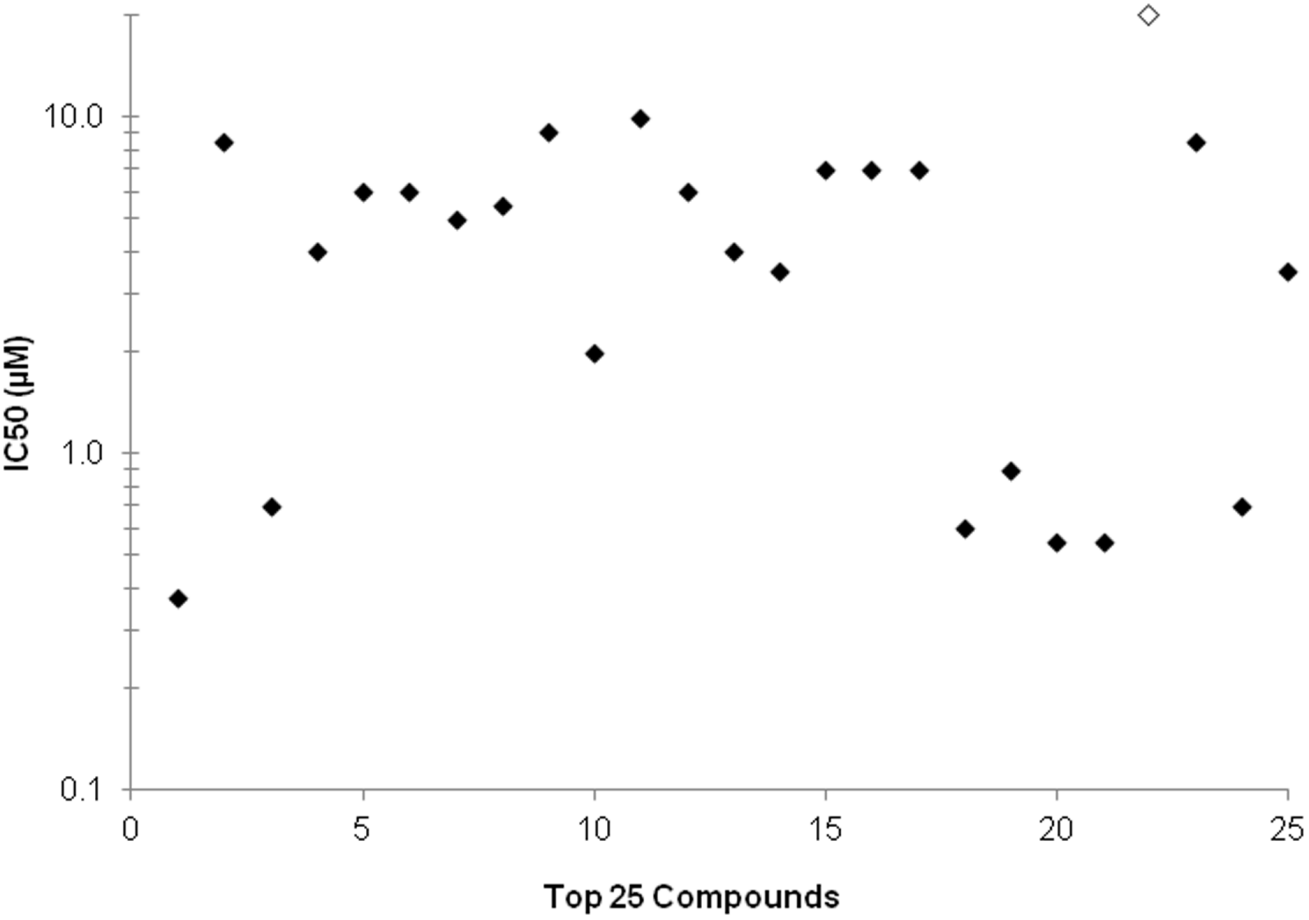
Plot of IC_50_ results. IC_50_ values less than or equal to 20 µM of 25 hits from FDA approved drug library screen. Primaquine (open diamond) demonstrated an IC_50_ value equal to 20 µM.

The Johns Hopkins University Clinical Compound Library version 1.3 of FDA-approved drugs was screened using the assay described above to identify compounds that had gametocytocidal activity, confirmed with Giemsa stained smears of drug treated cultures for the top hits. During the initial screening, clotrimazole was identified as a moderately active gametocytocidal compound, showing 70% inhibition at 20 µM and was then used as a lower cutoff control for identification of screening hits, in order to screen for compounds that were gametocytocidal and did not only inhibit exflagellation. Uninfected erythrocytes were used as baseline for the initial screening. The FDA approved drug library was screened at 20 µM and we initially selected the top 70 compounds showing more than 50% inhibition for evaluation using the gold standard of microscopic examination and IC_50_ determination at multiple concentrations (Figure 2, Table S1). As expected most hits showing more than 70% inhibition during initial screening were confirmed to be gametocytocidal by microscopic examination and they showed a clear dose dependent response. Overall we identified 25 compounds with IC_50_ values less than 20 µM, with most less than 10 µM (Table 1, Figure 3). Most of the compounds with intermediate activity were determined to inhibit exflagellation (data not shown) but were not gametocytocidal as indicated by Giemsa smears of drug-treated gametocytes. The mean Z-factor calculated from the FDA approved drug library screen was 0.52 (SEM = 0.07, Table S2).

**Table 1:** Gametocytocidal compounds identified in JHU FDA-approved drug library screen with greater than 70% inhibition and/or IC50≤20 µM.

		Gametocyte		Asexual	
Compound	Indication	20 µM % inh	Avg µM IC_50_	10 µM % inh (SD)	Avg µM IC_50_
**Melphalan**	Antineoplastic	151	4.0	22.9 (2.3)	20.0
**Gentian violet**	Antiseptic	148	8.5	99.2 (0.6)	0.6
**Homidium (Ethidium) bromide**	Anthelminthic	148	0.38	99.3 (0.0)	0.1
**Ifosfamide**	Antineoplastic	136	2.0	0.0 (0.0)	NA
**Pentamidine**	Antiprotozoal	129	0.7	97.7 (0.0)	1.0
**Thonzonium bromide**	Antiseptic	113	6.0	98.1 (0.0)	6.3
**Cetalkonium chloride**	Antiseptic	112	6.0	93.9 (0.1)	12.6
**Benzethonium chloride**	Antiseptic	112	6.0	98.6 (0.0)	4.0
**Cetylpyridinium bromide**	Antiseptic	110	9.0	86.4	6.3
**Benzalkonium chloride**	Antiseptic	109	7.0	98.5 (0.0)	5.0
**Methylbenzethonium chloride**	Antiseptic	108	10.0	98.3 (1.4)	-
**Pyrvinium pamoate**	Anthelminthic	103	4.0	99.6	0.6
**Maprotiline**	Antidepressant	102	0.9	37.4 (0.9)	20
**Anastrozole**	Antineoplastic	102	0.6	-	NA
**Cetylpyridinium chloride**	Antiseptic	99	7.0	66.4 (8.8)	-
**Benzododecinium chloride**	Antiseptic	98	5.0	98.0†	0.1‡
**Tilorone**	Antiviral	98	5.5	99.2 (0.0)	0.2‡
**Dithiazanine iodide**	Anthelminthic	95	7.0	92.9 (0.28)	3.2
**Pyrithione zinc**	Antiseptic	93	0.6	98.6 (0.9)	-
**Antimony potassium tartrate**	Anthelminthic	90	3.5	97.7 (0.0)	NA
**Primaquine**	Antimalarial	76	20	84.2 (10.0)	1.3
**Anazolene sodium**	Diagnostic aid	72	0.6	−6.3 (8.9)	20.0
**Megestrol acetate**	Progestogen	69	3.5	−0.7 (5.7)	NA
**Acetomenaphthone**	Pharmaceutic aid	66	8.5	-	12.6
**1-Pentanol**	Dermatologic	61	0.7	5.2 (12.5)	-
**Clotrimazole**	Antifungal	55	-	-	1.3

Asexual stage 10 µM inhibition data was obtained from the Collaborative Drug Discovery Database (CDDD), 10 µM drug 3D7 48 hr, ^3^H hypoxanthine assay for parasite inhibition protocol, and asexual IC_50_ data was obtained from from Eastman et al. or from the CDDD WRAIR IC 50 nM D6 protocol as noted [47], [48]. Gametocytocidal IC_50_ values were calculated from one experiment with three replicates for top compounds.

† Data only available for 96 hr assay,

‡ WRAIR D6 data, – Unavailable, NA not active.

### Major drug classes with gametocytocidal activity

**Figure 4:**
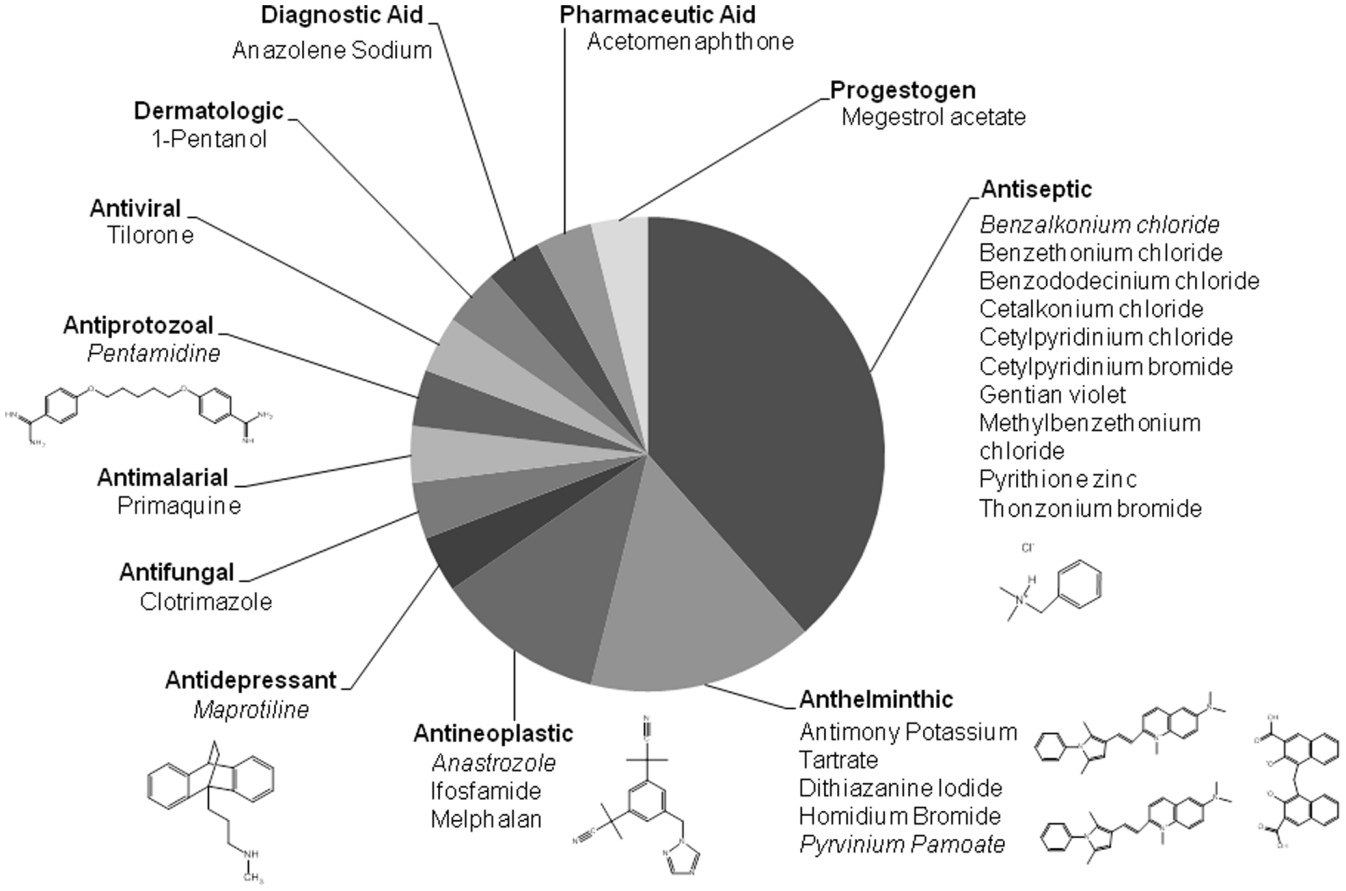
Drug class representation of active molecules. Classes of drug indications identified with activity against gametocytes with an IC_50_≤20 µM. Structures shown correspond to italicized compounds.

As a result of the FDA drug library screen, several drug classes were identified that showed activity against gametocytes, including a known antimalarial, primaquine, as well as other classes including antiseptics, antineoplastics, antihelminthics, antivirals, antiprotozoals, antidepressants, and pharmaceutical aids (Figure 4, complete list of indications is in Table S3). Eight of the twenty-five positive hits were identified as a single class of drugs, quaternary ammonium compounds (QACs) which were classified as antiseptics. Pyrvinium pamoate, an anthelminthic, demonstrated 100% inhibition at 10 µM and was used for further assays as a positive control for 100% inhibition or ‘killed’ parasite control in the presence of the background suppressor. By using a positive control of killed parasites in conjunction with the background suppressor rather than uninfected red blood cells, we were able to prevent artificially high inhibition values and screen for live gametocytes, not total gametocytes.

### Validation of gametocytocidal compounds with membrane feeding assay

**Figure 5:**
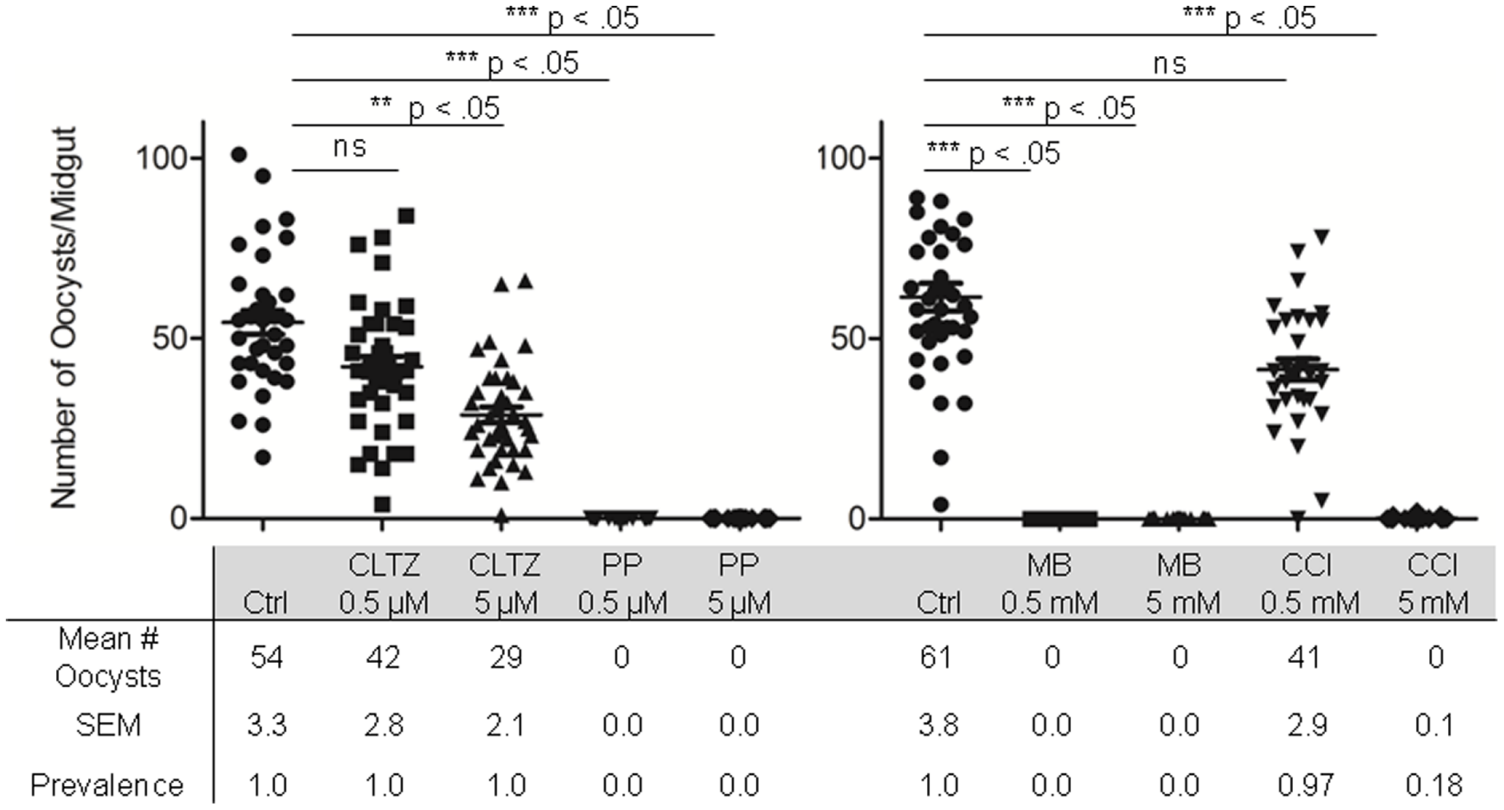
Inhibition of oocyst development of top compounds from JHU FDA-approved clinical compound library. Dose dependent transmission blocking activity of clotrimazole (CLTZ), pyrvinium pamoate (PP), methylene blue (MB) and cetalkonium chloride (CCl) measured by number of oocysts per mosquito midgut.

In order to validate the transmission blocking activity of compounds exhibiting the most potent gametocytocidal activity, mosquito infections through feeding on treated and untreated gametocyte cultures were performed. Gametocyte cultures were treated with methylene blue, a known gametocytocidal compound, clotrimazole, pyrvinium pamoate, and one of the quaternary ammonium compounds cetalkonium chloride for 48 hrs prior to ingestion by mosquitoes through a membrane feeder, and mosquito infections were determined 7 days later as a measure of oocyst stage parasite on the mosquito midgut tissue (Figure 5). All compounds demonstrated dose dependent transmission blocking activity, with pyrvinium pamoate showing the highest potency with 100% efficacy at 500 nM.

### MMV malaria box screen

**Figure 6:**
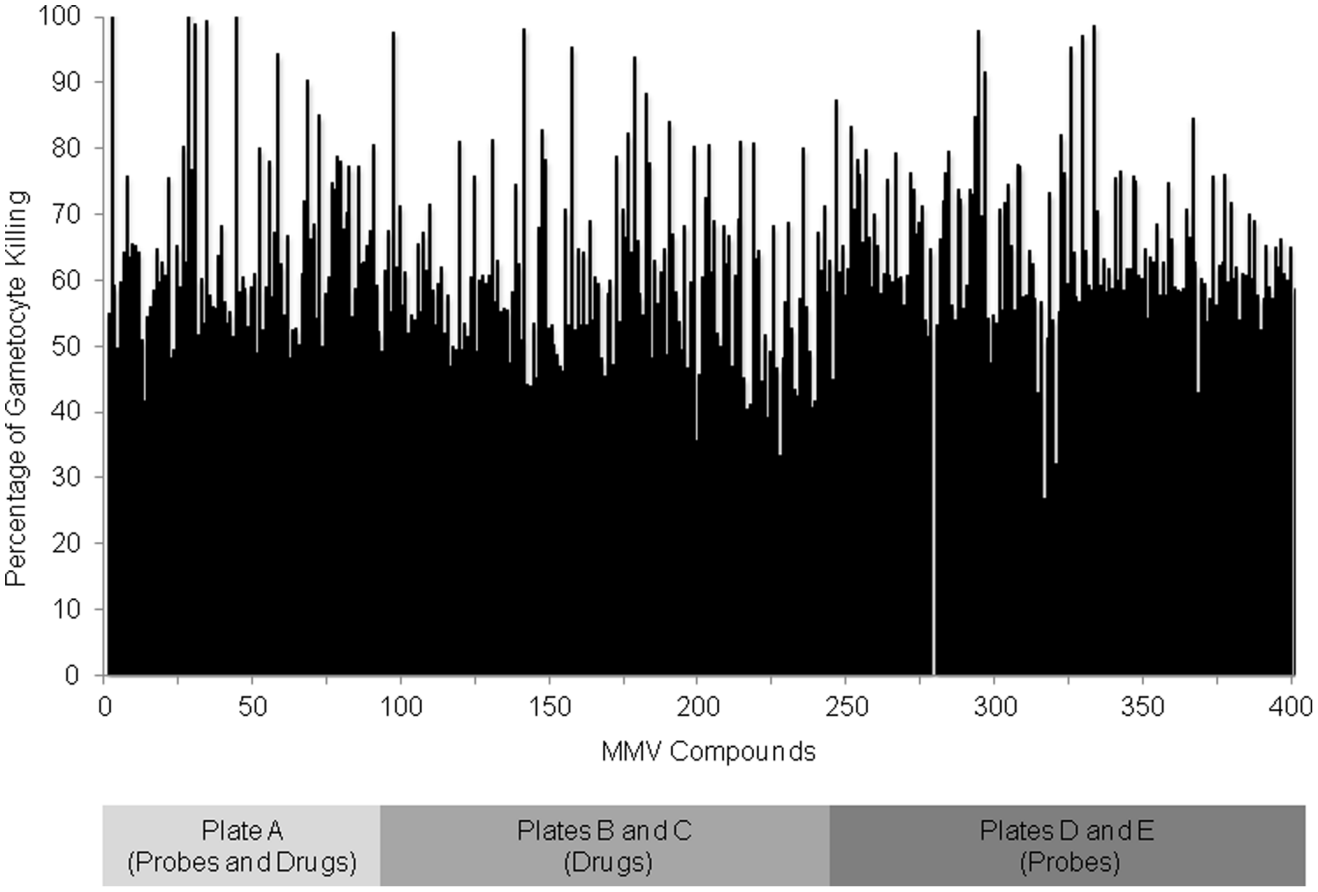
Inhibition by MMV Malaria Box. SYBR Green I assay results for the MMV box screened at 10 µM. Plot of percentage of gametocytocidal activity of 400 compounds compared to pyrvinium pamoate control.

Medicines for Malaria Venture (MMV) has generously put together a ‘malaria box’ of four hundred compounds with proven antimalarial activity against asexual blood stage parasites and made them freely available for use in the development of effective antimalarial compound screens, particularly those designed to identify liver stage and transmission blocking drugs. We screened these four hundred compounds using our gametocytocidal assay, this time using pyrvinium pamoate as a positive control due to increased efficacy compared to clotrimazole (Figure 6, Table S4). Our initial screen of the MMV box identified eighteen compounds with greater than 80% inhibition at 10 µM which we further screened to determine their IC_50_s (Table 2). Seventeen of the compounds were confirmed as having greater than 50% inhibition at 10 µM and IC_50_s less than 10 µM, with one compound MMV019918 showing a submicromolar IC_50_. Of these seventeen compounds with gametocytocidal activity, seven were drug-like, while ten were probe-like, as described by MMV [13]. In addition, compounds with the greatest activity against gametocytes also showed nanomolar IC_50_s against the asexual stage parasite as reported with the compound information by MMV. The mean Z-factor calculated from the MMV malaria box screen was 0.57 (SEM = 0.04).

**Table 2:** Gametocytocidal compounds identified from MMV Malaria Box with greater than 50% inhibition at 10 µM and available corresponding data on asexual stage inhibition from MMV.

	Gametocyte			Asexual	
MMV #	% inh 10 µM	IC_50_ (SD, µM)	% inh 5 µM	EC_50_ (nM)	CHEMBL EC_50_ (µM)
**MMV665941**	122	1.8 (0.2)	96	255	0.62
**MMV000448**	110	5.4 (1.4)	95	235	0.03, 1.04, 0.53
**MMV006172**	104	2.6 (0.3)	97	142	0.057, 0.64
**MMV396797**	100	8.8 (1.2)	-	477	NA
**MMV665878**	100	1.1 (0.6)	99	139	0.27
**MMV667491**	99	4.5 (1.5)	-	1230	NA
**MMV665830**	98	3.3 (0.6)	98	1005	0.25
**MMV019780**	98	3.8 (0.7)	98	697	0.84
**MMV019555**	97	3.4 (0.5)	100	376	0.20
**MMV019881**	96	5.5 (0.9)	98	646	1.04
**MMV019918**	92	0.9 (0.3)	96	801	1.51
**MMV019690**	90	>10	97	935	0.78
**MMV000445**	86	10.00	98	1135	1.97
**MMV007591**	85	5.4 (1.0)	85	ND	1.12
**MMV000848**	85	3.6 (3.2)	97	660	1.08
**MMV020505**	83	6.6 (1.1)	96	876	0.80
**MMV006303**	82	2.1 (0.6)	-	391	0.03
**MMV396794**	82	8.2 (0.9)	NA	1160	NA

**Figure 7:**
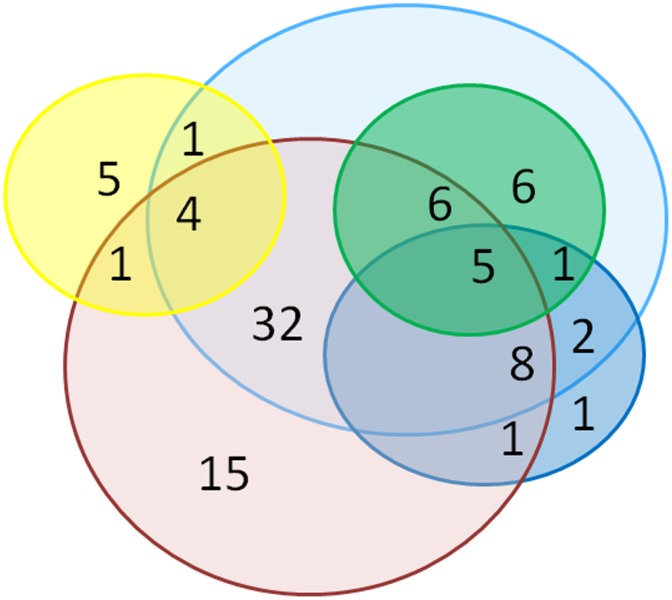
Overlap of recent screening assays for Malaria Box. Venn Diagram comparing our SYBR Green I assay (green) MMV box hits with hits from four other assays: Confocal fluorescence microscopy (red), Alamar blue early (dark blue) and late (light blue) and Luciferase (yellow) [10], [14]–[16].

Furthermore, we compared our MMV box hits with hits from four other assays using different reporters, including luciferase-expressing parasites, alamar blue, or confocal fluorescence microscopy [10], [14]–[16]. We found that all of our eighteen hits overlapped between different assays (Figure 7, Table S5).

## Discussion

To realize the goal of malaria elimination and eradication we need to add new and potent weapons active against multiple life stages of the parasite. Because most of the currently licensed antimalarials target only the asexual intra-erythrocytic stage, which is responsible for the pathology of disease, we urgently need to expand our antimalarial arsenal. Drugs which can effectively kill sexual gametocyte stages, responsible for transmission to the mosquito vector, will be required for malaria elimination. In order to find new tools we have established a simple and robust HTS gametocytocidal assay based on DNA content of live gametocytes. Because gametocytes do not multiply we have utilized male gametocyte exflagellation and a background suppressor to subtract the DNA fluorescence signals from dead cells to achieve a robust signal to noise ratio. As emphasized earlier in our description of assay optimization, we carefully took into consideration the contribution of exflagellation to fluorescent signal, and set cutoff values for our assay which allowed us to screen for compounds with gametocytocidal activity and not merely exflagellation inhibition. However, it should be noted that our assay does not allow us to distinguish between male and female gametocyte killing, but instead looks at overall live gametocytes, and at lower inhibition levels, male gametocyte viability. Because sex ratio tends to be biased in all *Plasmodium* species [17], we start out with two to four times as many females as males, and quantification is not skewed to a male exflagellation assay. Linearity of the assay was determined as a function of percent gametocytes at 2% HCT, which showed a linear relationship with an R2 value of ≥0.95. While many gametocytocidal assays have been developed, many of these assays have features that make them difficult to adapt to high-throughput screening such as multiple incubations steps, requirement for high gametocytemia, or transgenic parasites, making it impossible to use field isolates without further genetic manipulation. Our assay is simple enough to be used in any laboratory with access to malaria culture and a fluorescence plate reader, while also maintaining the sensitivity and robustness required for a high-throughput screening assay. We utilized our assay to screen an FDA-approved drug library of 1500 compounds as well as the MMVs Malaria box of 400 compounds to identify new pharmacophores with gametocytocidal activity.

Screening of the FDA approved drug library at 20 µM led to the identification of several classes of compounds with gametocytocidal activity. Most of the hits from FDA approved library were antiseptic, anthelminthic, and antineoplastic compounds as well as some antimicrobials and an antidepressant drug. Clotrimazole, an antifungal, was identified as having 70% inhibition against gametocytes with an asexual IC_50_ of 1.3 µM, and was recently reported as a hit in another gametocytocidal screen [11]. Pyrithione zinc is an antiseptic which showed activity in our assay with high efficacy against both sexual and asexual stages of the parasite and was also recently reported in the screening of a different library for gametocytocidal drugs [10]. As expected our screen identified primaquine as a gametocytocidal compound, albeit at a higher than reported IC_50_ due to lack of metabolism to the highly effective phenolic metabolites of primaquine required for inhibition [18]. The antineoplastic compounds, anastrozole, ifosfamide, and melphalan, demonstrated greater than 50% inhibition at 0.55 to 4 µM concentrations against gametocytes (Table 1). The data available for melphalan showed 20% inhibition of 3D7 at 10 µM and an IC_50_ of 20 µM compared to an IC_50_ of 4 µM against *P. falciparum* gametocytes, suggesting that melphalan shows slightly less efficacy against asexual compared to sexual parasites. Of the anthelminthics, homidium bromide and pyrvinium pamoate demonstrated the highest efficacy against gametocytes, with 100% inhibition at 20 µM and IC_50_ values of 0.38 µM and 4 µM respectively, while also effectively inhibiting 70–100% of asexual stages at 10 µM. Homidium bromide (ethidium bromide) is a well-known fluorescent DNA-intercalating agent used in molecular biology and is known to be mutagenic, whereas pyrvinium pamoate is an FDA-approved anthelminthic compound used to treat pinworm, with activity against *Cryptosporidium parvum*, and thought to inhibit mitochondrial NADH-fumarate reductase [19]–[21]. A recent study demonstrates nanomolar inhibition of pyrvinium pamoate against both 3D7 and K1 strains of *P. falciparum* asexual blood stage parasites with further derivatization studies suggesting the quaternary amino group in the quinoline ring is not required for antimalarial activity [22]. Removing the positive charge from the molecule may allow better bioavailability of pyrvinium pamoate, and further investigation of gametocytocidal activity of uncharged derivatives is warranted. The other anthelminthics antimony potassium tartrate and dithiazanine iodide inhibited 90% of gametocytes at 20 µM and 90% of asexual stages at 10 µM. Dithiazanine iodide has some structural similarity to pyrvinium pamoate and also possesses a quaternary amine, which raises the question of whether a positive charge is critical for gametocytocidal activity. Interestingly, maprotiline, a tetracyclic antidepressant similar to the tricyclic antidepressant methylene blue, demonstrated nanomolar inhibition of both gametocyte and asexual stages of *P. falciparum*, but showed greater efficacy against gametocytes. Methylene blue has reported efficacy against gametocytes *in vitro* and also showed *in vivo* efficacy against asexual parasites in multiple murine models of cerebral malaria, protecting 75% of mice at 10 mg/kg for five days post-infection [9], [23]–[26]. Our observations suggest further exploration of tetracyclic and tricyclic antidepressants for gametocytocidal activity.

The antiseptic QACs were the most highly represented class of drugs in the hits from the FDA approved library screen, comprising eight out of twenty five hits. Most of the QACs identified in the screen, including cetalkoniumchloride, thonzonium bromide, and benzododecinium chloride, demonstrated almost 100% efficacy against gametocytes at 20 µM with low micromolar IC_50_s. QACs with antimicrobial activities were identified as early as the 1930s and are among the most useful antiseptics and disinfectants, and have been used for a variety of clinical purposes [27]–[31]. These drugs can function as choline analogs and can inhibit the de novo phosphatidylcholine biosynthetic pathway of the malaria parasite. QACs have previously been shown to inhibit asexual blood stages of *P. falciparum* at nanomolar concentrations, with greater activity seen with long alkyl side chains and increased steric hindrance around the nitrogen atom [32]. Phosphatidylcholine, the predominant phospholipid produced by malaria parasites, plays essential structural and regulatory roles in parasite development and differentiation. Previous studies in *P. falciparum* have demonstrated the presence of two pathways for phosphatidylcholine biosynthesis, the cytidine diphosphate (CDP)-choline pathway, which uses host choline and fatty acids as precursors, and the serine decarboxylase-phosphoethanolamine methyltransferase (SDPM) pathway, which uses host serine and fatty acids as precursors. Recent studies have shown that QACs inhibit multiple steps during phospholipid biosynthesis by targeting the choline carrier as well as enzymes of both the SDPM and the CDP–choline pathways [33], [34]. A recently published study demonstrates the essentiality of phosphotidylcholine synthesis for gametocyte development and transmission by knocking out or inhibiting the key enzyme in this pathway, phosphoethanolamine methyl transferase, which results in inhibition of gametocyte maturation and also blocks transmission [35]. These observations strongly suggest a critical role for phospholipid metabolism during *P. falciparum* gametocyte stages and may present a unique target for multistage drug development. While challenges with poor absorption have been associated with this group of compounds due to a net positive charge, improvements using a prodrug approach have shown promise [32]. A choline analog, Albitiazolium is already in clinical trials for complicated malaria using intra-peritoneal or intra-muscular routes, and efforts are underway to develop this compound for uncomplicated malaria using an oral route [36]. Thus we have not only identified a class of compounds with efficacy against both asexual and sexual stages but also a shared target which can be utilized to identify new pharmacophores active against both asexual and transmission stages of malaria parasites.

In regards to cytotoxicity, route of drug administration and approved drug levels for the aforementioned hits, many of the compounds identified, including the QACs, are topical agents which are not approved for oral drug use. Anthelminthic compounds such as pyrvinium pamoate and dithiazanine iodide are approved for oral administration, but are not absorbed to appreciable levels by the GI tract and thus are not available in the bloodstream. Antineoplastics such as melphalan can be given orally or intravenously, but perhaps not surprisingly have side effects including bone marrow suppression. Maprotiline, however, is an orally administered antidepressant with an LD_50_ of 90 mg/kg in women, according to DrugBank, and approved prescription of 75–150 mg daily, depending on the severity of depression [37], [38]. While many of these FDA approved drug hits may not be immediately available or appropriate for oral antimalarial chemotherapy, they do provide novel pharmacophores with gametocytocidal and/or asexual activity, and are suggestive of new drug targets.

The successful screening and hit identification from FDA approved library led us to request the 400 compound Malaria Box of asexual blood stage active compounds from MMV. We screened the Malaria Box at 10 µM in duplicate, this time using 10 µM pyrvinium pamoate as a positive control (100% inhibition) and 0.1% DMSO as a vehicle control. As compared to the FDA approved library, we observed a higher number of compounds showing inhibition, which was expected as all these compounds have potent activity against the asexual blood stages. In all we obtained 18 hits, 17 of which showed a dose dependent response against mature gametocytes. The majority of the active compounds were very similar in structure, with seven containing acridine-like structures, three fused benzene rings with a central nitrogen, with varied side chains, one similar to that of chloroquine (MMV665830). Quinacrine and pyronaridine are both acridine-based compounds which have been proven clinically effective against malaria [39]. Multiple mechanisms of action have been proposed and proven for the various acridine-like compounds, including inhibition of hemozoin crystallization [40]–[42], mitochondrial *bc*_1_ complex [43], [44], DNA Topoisomerase II [45], [46], and also DNA intercalation, though the latter has not been correlated with increased antimalarial activity [39]. Of note, pyronaridine and other Topo II inhibitors have been shown to inhibit both asexual and sexual stages of *P. falciparum* in a previous study, suggesting that Topoisomerase II inhibitors may be utilized to target multiple parasite stages including gametocytes [45]. Towards the end of our library screening and data analysis, four manuscripts describing results of gametocytocidal screening of the MMV malaria box were published. Comparing our MMV hits with these four recent assays, we found that all of our hits overlapped with either the early or late alamar blue or confocal microscopy assays or both, but no hits were shared with the early gametocyte luciferase-based assay (Figure 7, Table S5) [10], [14]–[16]. MMV019918 was a top hit identified by four assays, including our SYBR Green I, the alamar blue and confocal microscopy assays, with nanomolar inhibition against late and early stages (IC_50_s ranging from 320–890 nM depending on the assay). Four other compounds including MMV000448, MMV006172, MMV007591 and MMV019555 were also identified by all four assays.

## Conclusions

We have successfully produced and validated a gametocytocidal drug screening assay that will be easily adaptable to high-throughput format using SYBR Green I and a background suppressor to read DNA content of live gametocytes after exposure to drug. Using this assay we screened an FDA-approved drug library and the MMV Malaria box, totaling approximately 2000 compounds and identified two highly represented classes of compounds, QACs and acridine-like compounds, which were effective against both sexual and asexual stages of the parasite. Further target validation is required to ascertain the mechanism of action of these compounds in gametocytes.

## Supporting Information

Table S1 **Top 70 Compounds FDA library screen IC50 data and Giemsa stained blood films.**
(PDF)

Table S2 **Controls and Z-factor analysis.**
(PDF)

Table S3 **Complete list of indications of all compounds in the Johns Hopkins Clinical Compound Library version 1.3.**
(PDF)

Table S4 **MMV Malaria Box Screen Data and Analysis.**
(PDF)

Table S5 **Compilation of top MMV malaria box hits from four gametocyte assays with three different reporters compared to the SYBR Green assay hits.**
(PDF)

Table S6 **FDA drug library SYBR Green I fluorescence data and analysis.**
(PDF)

Table S7 **Membrane feeding assay data.**
(PDF)
