# Supplementary material for: Gametocytocidal Screen Identifies Novel Chemical Classes with Plasmodium falciparum Transmission Blocking Activity
Source: PLoS One. 2014 Aug 26;9(8):e105817. doi: 10.1371/journal.pone.0105817 (PMC4144897; doi:10.1371/journal.pone.0105817)
Supplement: Table S1 — Top 70 Compounds FDA library screen IC50 data and Giemsa stained blood films. (PDF) [file pone.0105817.s001.pdf]

Table S1. Top 70 Compounds FDA Library Screen IC50 Data andGiemsa Stained blood films

compound	um drug	rep1	rep2	rep3	avg	stdev	avg-blank	%inhibition	IC_{50} (µM)	Giemsa blood film @ 10 µM
No drug	-	-	-	-	-	-	-	-	-	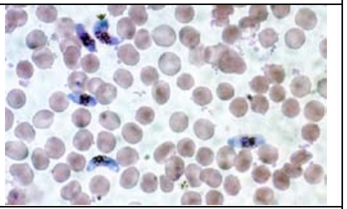
indatraline	20	15164	15723		15444	395	8909	2.5	>20	
	2	15537	14747		15142	559	8607	5.8		
	0.2	14944	15333		15139	275	8604	5.8		
homidium bromide, homidium bromide (ethidium bromide)	20	4932	7143		6038	1563	-498	105.4	0.375	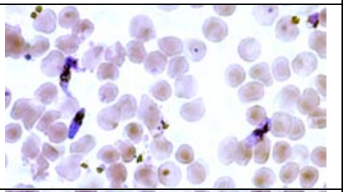
	2	5949	5880		5915	49	-621	106.8		
	0.2	13291	13205		13248	61	6713	26.5		
gentian violet	20	9505	10302		9904	564	3369	63.1	8.5	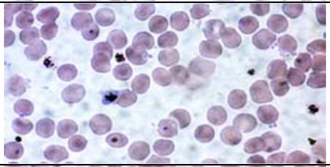
	2	12806	13012		12909	146	6374	30.2		
	0.2	14905	14967		14936	44	8401	8.1		
pentamidine	20	14403	14849		14626	315	8091	11.4	0.7	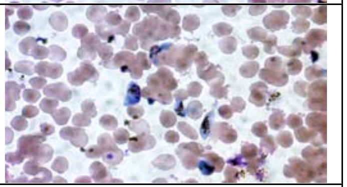
	2	8918	9157		9038	169	2503	72.6		
	0.2	15850	15417		15634	306	9099	0.4		
melphalan	20	5848	5488		5668	255	-867	109.5	4	
	2	13383	12324		12854	749	6319	30.8		
	0.2	13848	13980		13914	93	7379	19.2		
cetalkonium chloride, cetalkonium chloride (benzyldimethylhexadecy lammonium chloride)	20	6320	8119		7220	1272	685	92.5	6	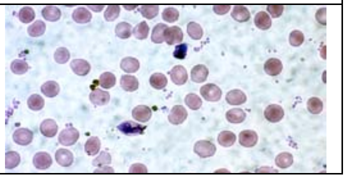

	2	13530	13718		13624	133	7089	22.4		
	0.2	13825	13573		13699	178	7164	21.6		
thonzonium, thonzonium bromide	20	6901	6476		6689	301	154	98.3	6	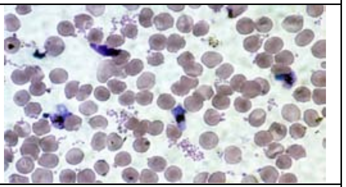
	2	14674	13817		14246	606	7711	15.6		
	0.2	14933	13935		14434	706	7899	13.5		
benzododecinium chloride, benzododecinium chloride (benzyl-dimethyl-dodecylammonium-chloride)	20	7282	7277		7280	4	745	91.9	5	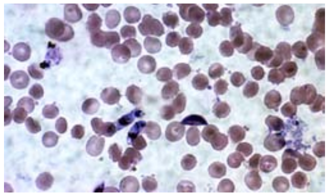
	2	13175	12983		13079	136	6544	28.4		
	0.2	13980	14924		14452	668	7917	13.3		
tilorone, tilorone hydrochloride, tilorone dihydrochloride	20	8017	8916		8467	636	1932	78.9	5.5	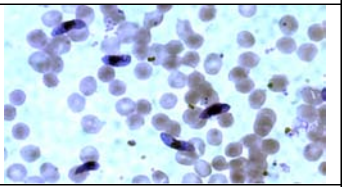
	2	12946	12833		12890	80	6355	30.5		
	0.2	14795	15468		15132	476	8597	5.9		
cetylpyridinium bromide monohydrate (no monohydrate), cetylpyridinium bromide monohydrate	20	8147	9456		8802	926	2267	75.2	9	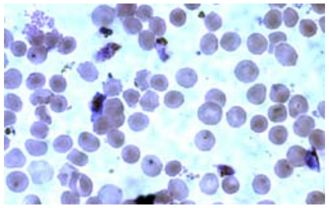
	2	14194	15194		14694	707	8159	10.7		
	0.2	14371	13901		14136	332	7601	16.8		
ifosfamide	20	6327	6420		6374	66	-162	101.8	2	
	2	11912	12122		12017	148	5482	40.0		
	0.2	14461	14134		14298	231	7763	15.0		
methylbenzethonium chloride	20	6885	11477		9181	3247	2646	71.0	10	
	2	14810	15354		15082	385	8547	6.5		

	0.2	14563	15150		14857	415	8322	8.9		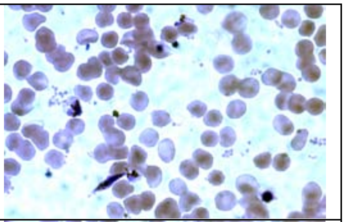
parthenolide	20	10298	12757		11528	1739	4889	38.8	>20	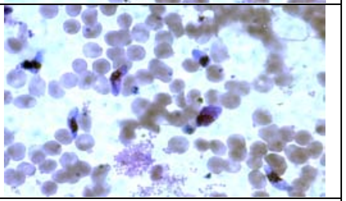
	2	14048	14698		14373	460	7734	3.1		
	0.2	13908	14071		13990	115	7351	7.9		
benzethonium chloride	20	7224	7148		7186	54	547	93.1	6	
	2	12529	13372		12951	596	6312	20.9		
	0.2	8329	14410		11370	4300	4731	40.7		
pyrvinium pamoate	20	6051	6265		6158	151	-481	106.0	4	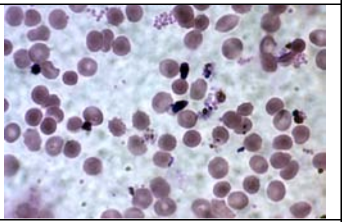
	2	11969	11419		11694	389	5055	36.7		
	0.2	8065	13531		10798	3865	4159	47.9		
antimony potassium tartrate, potassium antimonyl tartrate trihydrate, 99+%	20	6397	6882		6640	343	1	100.0	3.5	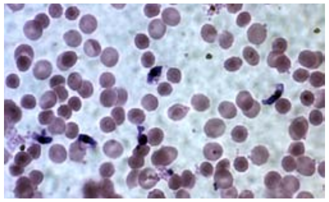
	2	10783	11955		11369	829	4730	40.7		
	0.2	8850	14538		11694	4022	5055	36.7		
piperine	20	14381	8871		11626	3896	4987	37.5	>20	
	2	13408	14172		13790	540	7151	10.4		
	0.2	8524	13163		10844	3280	4205	47.3		
benzalkonium chloride	20	6352	9050		7701	1908	1062	86.7	7	

	2	13622	13799		13711	125	7072	11.4		
	0.2	14795	13975		14385	580	7746	3.0		
cetylpyridinium, cetylpyridinium chloride, cetylpyridinium (pyrisept)	20	7153	7249		7201	68	562	93.0	7	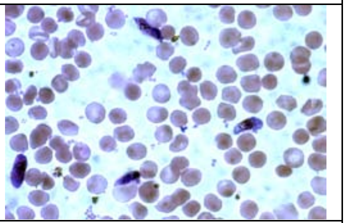
	2	13605	14079		13842	335	7203	9.8		
	0.2	14271	13565		13918	499	7279	8.8		
dithiazanine iodide, dithiazanine iodide (3,3 diethylthiadicarbocyanine iodide)	20	8444	6596		7520	1307	881	89.0	7	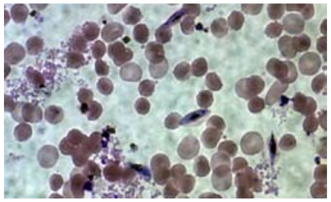
	2	13369	13832		13601	327	6962	12.8		
	0.2	14930	14041		14486	629	7847	1.7		
pyrithione, pyrithione zinc, pyrithione zinc (1-hydroxypyridine-2-thione zinc salt), 2-mercaptopyridine n-oxide	20	7868	6997		7433	616	794	90.1	0.6	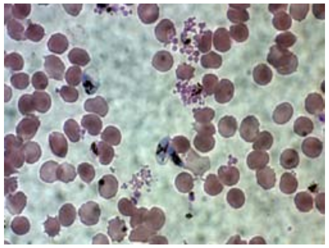
	2	7436	7152		7294	201	655	91.8		
	0.2	14032	14641		14337	431	7698	3.6		
maprotiline, maprotiline hydrochloride	20	8372	8160		8266	150	1627	79.6	0.9	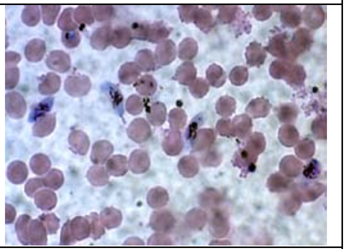
	2	9405	9006		9206	282	2567	67.8		
	0.2	14248	13874		14061	264	7422	7.0		
rufloxacin, rufloxacin hcl	20	8456	14328		11392	4152	4753	40.5	>20	
	2	14200	15048		14624	600	7985	0.0		

	0.2	14577	14187		14382	276	7743	3.0		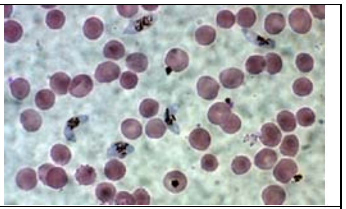
vinblastine, vinblastine sulfate	20	11130	13005		12068	1326	5429	32.0	0.9?	
	2	8825	8574		8700	177	2061	74.2		
	0.2	14584	14861		14723	196	8084	-1.3		
roxithromycin	20	16478	16019		16249	325	9610	-20.4	>20	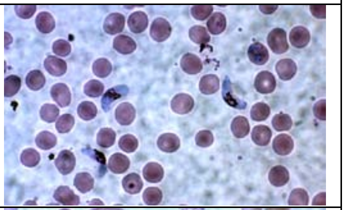
	2	13923	9058		11491	3440	4583	50.0		
	0.2	14739	15421		15080	482	8172	10.8		
pituitary acetone powder from carp	20	14895	14992		14944	69	8036	12.3	>20	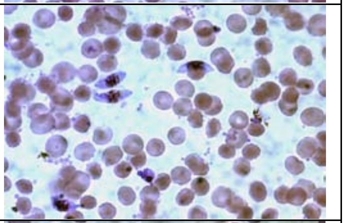
	2	14258	14162		14210	68	7302	20.3		
	0.2	14993	13735		14364	890	7456	18.6		
anazolene sodium, anazolene sodium (acid blue 92)	20	15697	14749		15223	670	8315	9.2	0.6	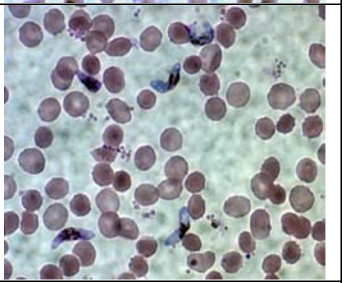
	2	8934	8569		8752	258	1844	79.9		
	0.2	14598	14581		14590	12	7682	16.1		
anastrozole	20	7562	7764		7663	143	755	91.8	0.55	
	2	8670	8427		8549	172	1641	82.1		
	0.2	14244	14531		14388	203	7480	18.3		
primaquine, primaquine phosphate, primaquine	20	12544	12863		12704	226	5796	36.7	>20	

diphosphate										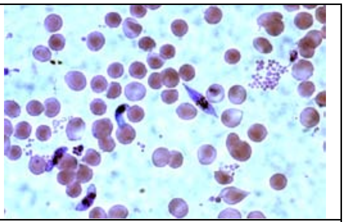
	2	14104	14733		14419	445	7511	18.0		
	0.2	14289	15363		14826	759	7918	13.5		
glucose, agar, galactose, l-galactose, d-(+)-mannose, glucose (d)	20	14627	13463		14045	823	7137	22.1	>20	
	2	13446	13532		13489	61	6581	28.1		
	0.2	13816	13285		13551	375	6643	27.5		
nifursol	20	14465	14276		14371	134	7463	18.5	>20	
	2	14079	13958		14019	86	7111	22.4		
	0.2	14906	14060		14483	598	7575	17.3		
picotamide, picotamide monohydrate	20	14172	15216		14694	738	7786	15.0	>20	
	2	14603	13582		14093	722	7185	21.6		
	0.2	14542	13931		14237	432	7329	20.0		
acetomenaphthone	20	8934	8444		8689	346	1781	80.6	8.5	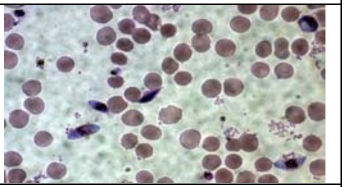
	2	15923	15078		15501	598	8593	6.2		
	0.2	13894	15159		14527	894	7619	16.8		
medroxyprogesterone acetate	20	14481	13774		14128	500	7220	21.2	>20	
	2	13801	14193		13997	277	7089	22.6		
	0.2	14370	13683		14027	486	7119	22.3		
proscillaridin, proscillaridin a	20	14443	14593		14518	106	7610	16.9	>20	

	2	14402	14863		14633	326	7725	15.7		
	0.2	13520	14370		13945	601	7037	23.2		
insulin lispro	20	14415	15178		14797	540	7889	13.9	>20	
	2	14635	13936		14286	494	7378	19.4		
	0.2	14661	14418		14540	172	7632	16.7		
		15924	16071	16204	16066	140	9158	0.0		
		7236	7136	6352	6908	484	0	100.0		
estrone sodium sulfate, estrone-3-sulfate, estrone	20	15938	15444		15691	349	8568	6.4	>20	
	2	15472	14634		15053	593	7930	4.8		
	0.2	15774	16008		15891	165	8768	-5.2		
phenformin, phenformin hydrochloride	20	15448	15584		15516	96	8393	-0.7	>20	
	2	14620	14125		14373	350	7250	13.0		
	0.2	15365	14346		14856	721	7733	7.2		
sodium fluoride	20	15853	15183		15518	474	8395	-0.8	>20	
	2	14368	14777		14573	289	7450	10.6		
	0.2	15826	15317		15572	360	8449	-1.4		
quazinone	20	15305	15424		15365	84	8242	1.1	>20	
	2	14816	14089		14453	514	7330	12.0		
	0.2	15951	16438		16195	344	9072	-8.9		
protriptyline, protriptyline hydrochloride	20	11414	14626		13020	2271	5897	29.2	>20	
	2	15885	15605		15745	198	8622	-3.5		
	0.2	14875	15712		15294	592	8171	1.9		
xanthinol niacinate, xanthinol niacinate (xanthinol nicotinate)	20	15060	14565		14813	350	7690	7.7	>20	

	2	15432	14025		14729	995	7606	8.7		
	0.2	16214	14205		15210	1421	8087	2.9		
pholcodine, pholedrine (p hydroxymethamphetamin e)	20	14933	14585		14759	246	7636	8.3	>20	
	2	15393	14296		14845	776	7722	7.3		
	0.2	15130	14340		14735	559	7612	8.6		
mestranol, ethynylestradiol 3-methyl ether, estriol methyl ether, mestranol (ethynylestradiol 3-methyl ester)	20	15183	14034		14609	812	7486	10.2	>20	
	2	15468	14205		14837	893	7714	7.4		
	0.2	15638	14264		14951	972	7828	6.0		
carbenicillin, carbenicillin disodium	20	15186	15529		15358	243	8235	1.2	>20	
	2	15752	15614		15683	98	8560	-2.7		
	0.2	14307	15792		15050	1050	7927	4.9		
protirelin, tamsulosin hcl, protirelin (pyroglu-his-pro amide, thyrotropin releasing hormone)	20	15751	14385		15068	966	7945	4.6	>20	
	2	15713	14290		15002	1006	7879	5.4		
	0.2	14314	14400		14357	61	7234	13.2		
phosphocreatine	20	15282	15250		15266	23	8143	2.3	>20	
	2	15319	14520		14920	565	7797	6.4		

	0.2	14245	15073		14659	585	7536	9.5		
syrosingopine	20	15864	15708		15786	110	8663	-4.0	>20	
	2	15354	14283		14819	757	7696	7.6		
	0.2	14709	14655		14682	38	7559	9.3		
		15147	15353	15863	15454	369	8332	0.0		
		7019	7393	6956	7123	236	0	100.0		
prazosin, prazosin hydrochloride	20	13219	12583		12901	450	6686	23.7	>20	
	2	14951	14963		14957	8	8742	0.2		
	0.2	14723	16111		15417	981	9202	-5.0		
methylthiouracil, methylthiouracil (6-methyl-2-thiouracil)	20	14430	14746		14588	223	8373	4.4	>20	
	2	14138	15011		14575	617	8359	4.6		
	0.2	13905	14153		14029	175	7814	10.8		
phentolamine, phentolamine hydrochloride, phentolamine methane-sulfonate	20	14907	15412		15160	357	8944	-2.1	>20	
	2	14115	14505		14310	276	8095	7.6		
	0.2	14234	14283		14259	35	8043	8.2		
physostigmine, eserine, eserine sulfate, physostigmine sulfate, physostigmine salicylate, physostigmine sulfate, eserine (physostigmine)	20	11127	11307		11217	127	5002	42.9	>20	
	2	11419	11379		11399	28	5184	40.8		

	0.2	15428	14813		15121	435	8905	-1.6		
pantethine, pantethine (d-pantethine)	20	15500	16032		15766	376	9551	-9.0	>20	
	2	15809	15552		15681	182	9465	-8.0		
	0.2	15907	15469		15688	310	9473	-8.1		
piperacetazine	20	13961	13930		13946	22	7730	11.8	>20	
	2	14467	14247		14357	156	8142	7.1		
	0.2	14397	13987		14192	290	7977	9.0		
nitrocefin	20	14392	14013		14203	268	7987	8.8	>20	
	2	14011	14464		14238	320	8022	8.4		
	0.2	14300	14247		14274	37	8058	8.0		
acarbose	20	14884	14411		14648	334	8432	3.8	>20	
	2	13550	12922		13236	444	7021	19.9		
	0.2	14115	13409		13762	499	7547	13.9		
insulin lispro	20	15124	15381		15253	182	9037	-3.1	>20	
	2	15980	15666		15823	222	9608	-9.7		
	0.2	14839	14657		14748	129	8533	2.6		
cabufocon (cellulose acetate butyrate)	20	13694	14297		13996	426	7780	11.2	>20	
	2	14511	15135		14823	441	8608	1.8		
	0.2	14244	14109		14177	95	7961	9.1		
mebutamate, mebutamate (2-methyl-2-(1-methylpropyl)-1,3-propanediol dicarbamate)	20	14477	14659		14568	129	8353	4.7	>20	
	2	16144	14124		15134	1428	8919	-1.8		

	0.2	14551	14253		14402	211	8187	6.6		
travoprost, travoprost (fluprostenol isopropyl ester)	20	16183	15504		15844	480	9628	-9.9	>20	
	2	16117	14569		15343	1095	9128	-4.2		
	0.2	14668	14133		14401	378	8185	6.6		
		14939	14987	15006	14977	35	8762	0.0		
		6665	5859	6122	6215	411	0	100.0		
colesevelam hydrochloride, colesevelam, colsevelam	20	14552	14876		14714	229	8198	7.4	>20	
	2	15261	14256		14759	711	8242	6.9		
	0.2	15025	14975		15000	35	8484	4.2		
risedronate, risedronic acid, risedronate sodium	20	14616	13931		14274	484	7757	12.4	>20	
	2	13730	14256		13993	372	7477	15.5		
	0.2	14063	13618		13841	315	7324	17.3		
estropipate	20	8425	15024		11725	4666	5208	41.2	>20	
	2	13536	15084		14310	1095	7794	12.0		
	0.2	14499	8880		11690	3973	5173	41.6		
polyoxyl 10 oleyl ether, polyoxyl 10 oleyl ether brij 92	20	9157	13535		11346	3096	4830	45.4	>20	
	2	14132	13846		13989	202	7473	15.6		
	0.2	14951	14011		14481	665	7965	10.0		
methyldopa, methyldopa (l,-), methyl-dopa (alpha-	20	14912	13693		14303	862	7786	12.1	>20	

methyl-l-beta-3,4-dihydroxyphenylalanine)										
	2	14230	14517		14374	203	7857	11.2		
	0.2	14309	14737		14523	303	8007	9.6		
niguldipine	20	13574	13540		13557	24	7041	20.5	>20	
	2	14192	13800		13996	277	7480	15.5		
	0.2	14507	14696		14602	134	8085	8.7		
nateglinide	20	13413	13887		13650	335	7134	19.4	>20	
	2	14673	8287		11480	4516	4964	43.9		
	0.2	14139	14904		14522	541	8005	9.6		
lomerizine, lomerizine hcl	20	13214	12194		12704	721	6188	30.1	>20	
	2	14130	12866		13498	894	6982	21.1		
	0.2	13723	13737		13730	10	7214	18.5		
lovastatin	20	13800	13511		13656	204	7139	19.4	0.35?	
	2	9196	8520		8858	478	2342	73.5		
	0.2	14730	9126		11928	3963	5412	38.9		
1-pentanol	20	8697	8381		8539	223	2023	77.2	0.7	
	2	8408	9372		8890	682	2374	73.2		
	0.2	14449	14388		14419	43	7902	10.7		
megestrol acetate	20	8677	8856		8767	127	2250	74.6	3.5	
	2	8256	15042		11649	4798	5133	42.0		
	0.2	14565	14690		14628	88	8111	8.4		
		15162	15581	15365	15369	210	8853	0.0		
		6223	6143	7183	6516	579	0	100.0		
