# Supplementary material for: Gametocytocidal Screen Identifies Novel Chemical Classes with Plasmodium falciparum Transmission Blocking Activity
Source: PLoS One. 2014 Aug 26;9(8):e105817. doi: 10.1371/journal.pone.0105817 (PMC4144897; doi:10.1371/journal.pone.0105817)
Supplement: Table S2 — Controls and Z-factor analysis. (PDF) [file pone.0105817.s002.pdf]

Table S2. Controls and Z-factor analysis

Library Screen 2							MMV Malaria Box					
Plate	µs	σs	µc	σc	Z	µs-µc	Plate	µs	σs	µc	σc	Z
1	14718	641	9255	340	0.46	5464	A	9950	575	19196	633	0.61
2	14147	208	8136	346	0.72	6011	A	9569	430	19591	515	0.72
3	14192	338	8190	272	0.70	6002	B	9806	690	19644	787	0.55
4	14601	416	8230	132	0.74	6371	B	10189	729	19305	729	0.52
5	14594	545	8256	155	0.67	6338	C	7976	486	15669	1331	0.29
6	12743	2353	8479	19	-0.67	4264	C	6379	185	12125	570	0.61
7	14851	468	8253	144	0.72	6598	D	8215	660	15765	591	0.50
8	14328	395	7762	330	0.67	6567	D	8309	194	16481	1028	0.55
9	14330	299	8298	1044	0.33	6033	E	6392	278	13133	394	0.70
10	14668	132	8002	1004	0.49	6665	E	6508	152	12904	557	0.67
11	14750	693	9554	510	0.31	5197						
12	14630	820	7464	341	0.51	7166					Mean	0.57
13	15242	194	9827	116	0.83	5416					Stdev	0.12
14	14278	627	9773	161	0.47	4505					SEM	0.04
15	14370	1017	9728	1010	-0.31	4642						
16	15501	1085	7541	373	0.45	7960						
20	13230	464	8430	306	0.52	4801						
31	14865	412	7643	138	0.77	7222						
32	15490	2315	8077	101	0.02	7414						
33	17309	694	8368	313	0.66	8941						
34	17699	98	8468	232	0.89	9231						
35	14479	245	7632	264	0.78	6848						
36	16271	262	7853	349	0.78	8418						
37	14803	594	9182	240	0.55	5621						
41	16455	646	8734	272	0.64	7722						
42	17623	687	8075	192	0.72	9549						
43	15775	767	7969	418	0.54	7805						
				Mean	0.52							
				StDev	0.35							
				SEM	0.07							
