# Supplementary material for: Gametocytocidal Screen Identifies Novel Chemical Classes with Plasmodium falciparum Transmission Blocking Activity
Source: PLoS One. 2014 Aug 26;9(8):e105817. doi: 10.1371/journal.pone.0105817 (PMC4144897; doi:10.1371/journal.pone.0105817)
Supplement: Table S3 — Complete list of indications of all compounds in the Johns Hopkins Clinical Compound Library version 1.3. (PDF) [file pone.0105817.s003.pdf]

## Table S3. Complete list of indications of all compounds in the Johns Hopkins Clinical Compound Library version 1.3

| Ref # | Indication | # Plotted | Ref # | Indication | # Plotted |
|---|---|---|---|---|---|
| 1 | Abortifacient | 1 | | Bronchodilator | 971-991 |
| | Alcohol deterrant | 2 | 15 | capillary protectant | 992-994 |
| | Analgesic | 4-41 | | Cardiotonic | 995-1008 |
| | Anasthetic | 42-69 | | Choleretic | 1009-1015 |
| 2 | Anorexic | 70 | | Cholinergic | 1016-1026 |
| | Antacid | 71-84 | | Decongestant | 1027-1037 |
| | Anthelminthic | 85-121 | | Dermatologic | 1038-1087 |
| 3 | Antiinflammatory | 122 | | Diagnostic aid | 1088-1122 |
| | Antiamebic | 123-127 | | Diuretic | 1123-1147 |
| | Antianginal | 128-134 | 16 | Ectoparasiticide, Dermatologic | 1148-1149 |
| | Antiarrhythmic | 125-143 | | Erectile dysfunction | 1150-1152 |
| 4 | Antiarthritic | 144-145 | | Estrogen, Steroid, Pituitary | 1153-1160 |
| | Antiasthmatic | 146-148 | | Expectorant | 1161-1167 |
| | Antibacterial | 149-204 | | Gastroprokinetic | 1168-1169 |
| | Antibiotic | 205-343 | | Glucocorticoid | 1170-1181 |
| 5 | Anticholelithogenic | 344-345 | 17 | Hematinic | 1182-1187 |
| | Anticoagulant | 346-351 | | Hemostatic | 1188-1195 |
| | Anticonvulsant | 352-375 | | Hepatoprotectant, Hepatic protectant, | 1196-1198 |
| | Antidepressant | 376-405 | | Immunomodulator | 1199-1201 |
| 6 | Antidiabetic | 406-420 | | Immunosuppressant | 1202-1205 |
| | Antidiarrheal | 421-423 | | Insecticide | 1206-1211 |
| | Antidote | 424-449 | | Laxative | 1212-1227 |
| | Antiemetic | 450-467 | 18 | Miscellaneous | 1228-1241 |
| 7 | Antiflatulent | 468-469 | | Mucolytic | 1242-1245 |
| | Antifungal | 470-500 | | Muscle relaxant (skeletal) | 1246-1254 |
| 8 | Antiglaucoma | 501-505 | 19 | Mydriatic | 1255-1263 |
| | Antihistaminic | 506-538 | | N/A | 1264-1274 |
| | Antihyperlipidemic | 539-558 | | Neuromuscular blocking agent | 1275-1280 |
| | Antihypertensive | 559-612 | | Nootropic | 1281-1308 |
| 9 | Antihypotensive | 613-625 | | Nutrient | 1309-1327 |
| | Antiinflammatory | 626-666 | 20 | Oxytocic | 1328-1329 |
| | Antimalarial | 667-677 | | Phamaceutic aid | 1330-1354 |
| 10 | Antimigraine | 678-682 | 21 | Pituitary | 1355 |
| | Antineoplastic | 683-741 | | Plasma volume expander | 1356-1357 |
| 11 | Antiobesity | 742 | | Progestogen | 1358-1369 |
| | Antiparkinsonian | 743-757 | | Respiratory stimulant | 1370-1371 |
| | Antiprotozoal | 758-770 | | Sedative | 1372-1392 |
| | Antipsychotic | 771-796 | 22 | Steroid | 1393-1402 |
| 12 | Antirheumatic | 798-799 | | Therapeutic plant extract | 1403-1407 |
| | Antiseizure | 800 | | Thyroid | 1408-1412 |
| | Antiseptic | 801-875 | | Tocolytic | 1413-1414 |
| | Antispasmodic | 876-902 | | Unclassified | 1415-1419 |
| 13 | Antithrombotic | 903-906 | | Uricosuric | 1420-1421 |
| | Antitussive | 907-916 | | Urologic | 1422-1425 |
| | Antiulcerative | 917-932 | | Vasodilator | 1426-1441 |
| | Antiurolithic | 933 | | Vitamin | 1442-1485 |
| | Antiviral | 934-956 | | Unclassified cont'd | 1486-1581 |
| 14 | Anxiolytic | 957-961 | | | |
| | Astringent | 963-964 | | | |
| | Bone resorption inhibitor | 965-970 | | | |
