# Supplementary material for: Gametocytocidal Screen Identifies Novel Chemical Classes with Plasmodium falciparum Transmission Blocking Activity
Source: PLoS One. 2014 Aug 26;9(8):e105817. doi: 10.1371/journal.pone.0105817 (PMC4144897; doi:10.1371/journal.pone.0105817)
Supplement: Table S4 — MMV Malaria Box Screen Data and Analysis. (PDF) [file pone.0105817.s004.pdf]

Table S4. MMV Malaria Box screen data and analysis

	HEOS_COMPOUND_ID	Plate_March2012	WELL_May2012_April2013	SYBR Green I reps 1 and 2		average	avg - µc	% inh
**1**	MMV019066	A	A02	17899	19144	18522	8762	54.8
**2**	MMV665941	A	A03	6344	6863	6604	-3156	116.3
**3**	MMV396680	A	A04	17164	18241	17703	7943	59.0
**4**	MMV666601	A	A05	18788	20247	19518	9758	49.7
**5**	MMV008294	A	A06	17077	18044	17561	7801	59.8
**6**	MMV011259	A	A07	16308	17103	16706	6946	64.2
**7**	MMV019406	A	A08	14190	14759	14475	4715	75.7
**8**	MMV006278	A	A09	16584	17131	16858	7098	63.4
**9**	MMV666688	A	A10	15661	17279	16470	6710	65.4
**10**	MMV019110	A	A11	16317	16687	16502	6742	65.2
**11**	MMV006427	A	B02	16719	16692	16706	6946	64.2
**12**	MMV666062	A	B03	19512	19038	19275	9515	50.9
**13**	MMV020885	A	B04	21551	20579	21065	11305	41.7
**14**	MMV000570	A	B05	18133	19070	18602	8842	54.4
**15**	MMV020439	A	B06	18787	17845	18316	8556	55.9
**16**	MMV396672	A	B07	17246	18384	17815	8055	58.5
**17**	MMV019871	A	B08	16482	16757	16620	6860	64.6
**18**	MMV085583	A	B09	17018	18187	17603	7843	59.6
**19**	MMV008416	A	B10	16802	17178	16990	7230	62.7
**20**	MMV665874	A	B11	17634	17167	17401	7641	60.6
**21**	MMV006203	A	C02	14351	14662	14507	4747	75.5
**22**	MMV665977	A	C03	18017	21586	19802	10042	48.2
**23**	MMV020549	A	C04	18844	20289	19567	9807	49.4
**24**	MMV001246	A	C05	15825	17191	16508	6748	65.2
**25**	MMV666607	A	C06	16819	18645	17732	7972	58.9
**26**	MMV665915	A	C07	13591	13599	13595	3835	80.2
**27**	MMV007695	A	C08	16756	17194	16975	7215	62.8
**28**	MMV000448	A	C09	4699	11145	7922	-1838	109.5
**29**	MMV020500	A	C10	14285	14246	14266	4506	76.8
**30**	MMV665878	A	C11	9999	9942	9971	211	98.9
**31**	MMV666101	A	D02	19191	19068	19130	9370	51.7
**32**	MMV666596	A	D03	16697	18258	17478	7718	60.2
**33**	MMV396679	A	D04	18789	18807	18798	9038	53.4
**34**	MMV396797	A	D05	9465	10297	9881	121	99.4
**35**	MMV008138	A	D06	17349	18613	17981	8221	57.6

**36**	MMV665916	A	D07	18191	18455	18323	8563	55.8
**37**	MMV020788	A	D08	18711	18040	18376	8616	55.6
**38**	MMV666691	A	D09	17416	16218	16817	7057	63.6
**39**	MMV665785	A	D10	15574	16272	15923	6163	68.2
**40**	MMV665831	A	D11	18087	18196	18142	8382	56.8
**41**	MMV011099	A	E02	18116	19467	18792	9032	53.4
**42**	MMV000642	A	E03	16168	20741	18455	8695	55.2
**43**	MMV666600	A	E04	18809	19578	19194	9434	51.4
**44**	MMV006172	A	E05	9396	8997	9197	-563	102.9
**45**	MMV006309	A	E06	16606	19155	17881	8121	58.1
**46**	MMV006087	A	E07	17228	17631	17430	7670	60.5
**47**	MMV020492	A	E08	16385	19212	17799	8039	58.5
**48**	MMV006455	A	E09	18461	19301	18881	9121	53.0
**49**	MMV665782	A	E10	17256	18176	17716	7956	59.0
**50**	MMV665876	A	E11	17062	17574	17318	7558	61.0
**51**	MMV666023	A	F02	19685	19676	19681	9921	48.8
**52**	MMV009063	A	F03	12812	14470	13641	3881	80.0
**53**	MMV006558	A	F04	18687	19341	19014	9254	52.3
**54**	MMV007160	A	F05	17186	18280	17733	7973	58.9
**55**	MMV006429	A	F06	12458	15568	14013	4253	78.1
**56**	MMV396703	A	F07	17916	18138	18027	8267	57.4
**57**	MMV006937	A	F08	16090	16132	16111	6351	67.3
**58**	MMV085203	A	F09	10837	10903	10870	1110	94.3
**59**	MMV665820	A	F10	16391	17710	17051	7291	62.4
**60**	MMV665841	A	F11	18474	18678	18576	8816	54.5
**61**	MMV007116	A	G02	16177	16215	16196	6436	66.8
**62**	MMV007384	A	G03	18963	20662	19813	10053	48.2
**63**	MMV020548	A	G04	17616	20338	18977	9217	52.5
**64**	MMV019258	A	G05	18136	19719	18928	9168	52.7
**65**	MMV007686	A	G06	17509	21331	19420	9660	50.2
**66**	MMV011256	A	G07	16146	18497	17322	7562	61.0
**67**	MMV666693	A	G08	15772	14653	15213	5453	71.9
**68**	MMV008956	A	G09	4796	18536	11666	1906	90.2
**69**	MMV665827	A	G10	15492	17119	16306	6546	66.2
**70**	MMV001038	A	G11	15045	16707	15876	6116	68.5
**71**	MMV007839	A	H02	18678	18671	18675	8915	54.0

**72**	MMV000662	A	H03	11793	13560	12677	2917	85.0
**73**	MMV396678	A	H04	19062	19907	19485	9725	49.9
**74**	MMV006861	A	H05	18220	17646	17933	8173	57.9
**75**	MMV006457	A	H06	15732	19095	17414	7654	60.5
**76**	MMV396693	A	H07	14233	15090	14662	4902	74.7
**77**	MMV011567	A	H08	14565	15107	14836	5076	73.8
**78**	MMV007907	A	H09	13055	14720	13888	4128	78.7
**79**	MMV665805	A	H10	12697	15358	14028	4268	78.0
**80**	MMV666021	A	H11	15011	17020	16016	6256	67.7
**81**	MMV665800	B	A02	15982	15604	15793	5796	70.2
**82**	MMV000634	B	A03	13409	15447	14428	4431	77.2
**83**	MMV666103	B	A04	18615	19102	18859	8861	54.5
**84**	MMV666057	B	A05	17474	18615	18045	8047	58.7
**85**	MMV007564	B	A06	15118	13749	14434	4436	77.2
**86**	MMV001255	B	A07	17280	17312	17296	7299	62.5
**87**	MMV665917	B	A08	17175	17386	17281	7283	62.6
**88**	MMV000563	B	A09	17355	16219	16787	6790	65.1
**89**	MMV665850	B	A10	16398	16310	16354	6357	67.4
**90**	MMV665817	B	A11	13569	14045	13807	3810	80.4
**91**	MMV665979	B	B02	17903	17973	17938	7941	59.2
**92**	MMV665928	B	B03	19068	19576	19322	9325	52.1
**93**	MMV666105	B	B04	20020	19800	19910	9913	49.1
**94**	MMV666072	B	B05	16934	18117	17526	7528	61.3
**95**	MMV000653	B	B06	16104	16553	16329	6331	67.5
**96**	MMV000620	B	B07	18642	18840	18741	8744	55.1
**97**	MMV665909	B	B08	10875	10018	10447	449	97.7
**98**	MMV665940	B	B09	18311	16503	17407	7410	62.0
**99**	MMV665891	B	B10	16111	15083	15597	5600	71.2
**100**	MMV665899	B	B11	18805	18239	18522	8525	56.2
**101**	MMV665961	B	C02	17485	17649	17567	7570	61.1
**102**	MMV665929	B	C03	19656	19046	19351	9354	52.0
**103**	MMV666108	B	C04	19207	18460	18834	8836	54.6
**104**	MMV665948	B	C05	19618	18380	18999	9002	53.8
**105**	MMV006188	B	C06	16644	16771	16708	6710	65.5
**106**	MMV001230	B	C07	19296	18186	18741	8744	55.1
**107**	MMV665918	B	C08	17541	15258	16400	6402	67.1

**108**	MMV665799	B	C09	18512	16494	17503	7506	61.5
**109**	MMV665826	B	C10	17013	14141	15577	5580	71.3
**110**	MMV665807	B	C11	18390	17839	18115	8117	58.3
**111**	MMV665946	B	D02	19215	18985	19100	9103	53.3
**112**	MMV665935	B	D03	18898	16943	17921	7923	59.3
**113**	MMV666102	B	D04	18485	16311	17398	7401	62.0
**114**	MMV666061	B	D05	20094	18598	19346	9349	52.0
**115**	MMV008149	B	D06	19133	17383	18258	8261	57.6
**116**	MMV019074	B	D07	20822	19872	20347	10350	46.9
**117**	MMV665914	B	D08	21123	18427	19775	9778	49.8
**118**	MMV665798	B	D09	21125	18632	19879	9881	49.3
**119**	MMV665902	B	D10	15318	12054	13686	3689	81.1
**120**	MMV665888	B	D11	19625	20122	19874	9876	49.3
**121**	MMV666067	B	E02	19348	18761	19055	9057	53.5
**122**	MMV665939	B	E03	19960	18960	19460	9463	51.4
**123**	MMV009060	B	E04	19065	16300	17683	7685	60.5
**124**	MMV666110	B	E05	10971	18489	14730	4733	75.7
**125**	MMV019758	B	E06	21129	18678	19904	9906	49.1
**126**	MMV000498	B	E07	18088	17481	17785	7787	60.0
**127**	MMV665913	B	E08	18184	17103	17644	7646	60.7
**128**	MMV665789	B	E09	18118	17669	17894	7896	59.5
**129**	MMV665901	B	E10	18530	16797	17664	7666	60.6
**130**	MMV666069	B	E11	14174	13121	13648	3650	81.3
**131**	MMV666080	B	F02	19926	16985	18456	8458	56.6
**132**	MMV666081	B	F03	17712	16716	17214	7217	62.9
**133**	MMV666009	B	F04	19366	18102	18734	8737	55.1
**134**	MMV019313	B	F05	19545	17678	18612	8614	55.8
**135**	MMV019746	B	F06	19593	17793	18693	8696	55.3
**136**	MMV019064	B	F07	20580	19883	20232	10234	47.4
**137**	MMV011944	B	F08	20102	16229	18166	8168	58.1
**138**	MMV665803	B	F09	16647	13326	14987	4989	74.4
**139**	MMV665857	B	F10	17340	17323	17332	7334	62.3
**140**	MMV666071	B	F11	19757	19333	19545	9548	51.0
**141**	MMV019780	B	G02	10119	10603	10361	364	98.1
**142**	MMV666093	B	G03	21157	20604	20881	10883	44.1
**143**	MMV665953	B	G04	24744	17138	20941	10944	43.8

**144**	MMV000648	B	G05	19889	18299		19094	9097	53.3
**145**	MMV019662	B	G06	22472	18895		20684	10686	45.1
**146**	MMV007571	B	G07	16572	15887		16230	6232	68.0
**147**	MMV007617	B	G08	14035	12695		13365	3368	82.7
**148**	MMV665796	B	G09	14499	13976		14238	4240	78.2
**149**	MMV665906	B	G10	20325	18130		19228	9230	52.6
**150**	MMV665954	B	G11	19706	18564		19135	9138	53.1
**151**	MMV019738	B	H02	19321	20063		19692	9695	50.2
**152**	MMV666075	B	H03	20136	19904		20020	10023	48.5
**153**	MMV666070	B	H04	20939	19785		20362	10365	46.8
**154**	MMV142383	B	H05	21559	19414		20487	10489	46.1
**155**	MMV000788	B	H06	16126	15322		15724	5727	70.6
**156**	MMV000561	B	H07	19058	19142		19100	9103	53.3
**157**	MMV000248	B	H08	11435	10411		10923	926	95.2
**158**	MMV665879	B	H09	19894	18630		19262	9265	52.4
**159**	MMV665890	B	H10	17872	15888		16880	6883	64.7
**160**	MMV666116	B	H11	19413	18850		19132	9134	53.1
**161**	MMV006913	C	A02	12140	15627	9806	13884	6018	64.2
**162**	MMV008127	C	A03	13700	17777	9823	15739	7873	53.1
**163**	MMV403679	C	A04	11441	14741	9518	13091	5225	68.9
**164**	MMV006545	C	A05	13828	17364	8932	15596	7730	54.0
**165**	MMV019700	C	A06	11906	17126	10409	14516	6650	60.4
**166**	MMV019670	C	A07	12282	17040	10822	14661	6795	59.5
**167**	MMV001344	C	A08	12930	20256	10223	16593	8727	48.0
**168**	MMV011795	C	A09	14869	19246	10149	17058	9192	45.3
**169**	MMV019124	C	A10	12228	17667	10100	14948	7082	57.8
**170**	MMV006767	C	A11	12232	16932	9681	14582	6716	60.0
**171**	MMV007808	C	B02	14451	19075	10205	16763	8897	47.0
**172**	MMV019017	C	B03	9748	13151	9187	11450	3584	78.7
**173**	MMV396681	C	B04	12480	18853	10439	15667	7801	53.5
**174**	MMV006587	C	B05	12521	13026	9597	12774	4908	70.8
**175**	MMV019202	C	B06	12918	14089	10320	13504	5638	66.4
**176**	MMV000848	C	B07	9951	11761	7048	10856	2990	82.2
**177**	MMV020275	C	B08	13129	14609	10368	13869	6003	64.2
**178**	MMV019918	C	B09	8495	9304	6581	8900	1034	93.8
**179**	MMV075490	C	B10	12167	14988	10522	13578	5712	66.0

**180**	MMV396633	C	B11	13634	16271	10063	14953	7087	57.8
**181**	MMV007374	C	C02	13690	17287	10593	15489	7623	54.6
**182**	MMV396719	C	C03	10324	9304	10241	9814	1948	88.4
**183**	MMV396744	C	C04	14068	9160	10385	11614	3748	77.7
**184**	MMV006706	C	C05	13537	19612	10416	16575	8709	48.1
**185**	MMV009108	C	C06	13624	14548	10285	14086	6220	63.0
**186**	MMV020700	C	C07	12759	17573	10174	15166	7300	56.5
**187**	MMV007906	C	C08	11887	16895	10427	14391	6525	61.1
**188**	MMV008270	C	C09	13532	14058	10584	13795	5929	64.7
**189**	MMV019127	C	C10	15177	17828	9841	16503	8637	48.6
**190**	MMV396794	C	C11	10296	10799	7502	10548	2682	84.0
**191**	MMV396736	C	D02	11257	15573	10068	13415	5549	67.0
**192**	MMV306025	C	D03	13039	16779	10749	14909	7043	58.1
**193**	MMV056726	C	D04	13550	17786	9747	15668	7802	53.5
**194**	MMV274073	C	D05	13719	18973	10558	16346	8480	49.5
**195**	MMV018984	C	D06	10970	15401	11015	13186	5320	68.3
**196**	MMV000911	C	D07	14333	19288	10082	16811	8945	46.7
**197**	MMV007430	C	D08	12274	17041	10121	14658	6792	59.6
**198**	MMV007977	C	D09	8529	13863	10182	11196	3330	80.2
**199**	MMV020654	C	D10	17206	20132	10616	18669	10803	35.7
**200**	MMV665883	C	D11	19086	14952	10500	17019	9153	45.5
**201**	MMV084940	C	E02	12934	16102	11228	14518	6652	60.4
**202**	MMV396715	C	E03	11904	13082	9796	12493	4627	72.4
**203**	MMV000963	C	E04	11379	10866	10867	11123	3257	80.6
**204**	MMV006319	C	E05	11895	16889	10474	14392	6526	61.1
**205**	MMV000972	C	E06	13350	12848	11022	13099	5233	68.8
**206**	MMV020490	C	E07	12727	19139	10266	15933	8067	52.0
**207**	MMV001318	C	E08	12791	19761	10774	16276	8410	49.9
**208**	MMV007978	C	E09	11435	14990	10393	13213	5347	68.2
**209**	MMV020660	C	E10	14594	13735	10007	14165	6299	62.5
**210**	MMV665904	C	E11	12309	14621	10857	13465	5599	66.7
**211**	MMV396632	C	F02	14889	18693	9916	16791	8925	46.8
**212**	MMV007875	C	F03	12096	16855	9762	14476	6610	60.6
**213**	MMV006820	C	F04	11170	14932	10464	13051	5185	69.1
**214**	MMV396749	C	F05	9558	12550	10288	11054	3188	81.0
**215**	MMV011576	C	F06	16298	17854	10395	17076	9210	45.2

**216**	MMV020651	C	F07	16963	18813	10618	17888	10022	40.3
**217**	MMV000483	C	F08	16454	19055	9792	17755	9889	41.1
**218**	MMV019266	C	F09	9841	12314	9627	11078	3212	80.9
**219**	MMV001049	C	F10	13358	14759	9487	14059	6193	63.1
**220**	MMV665806	C	F11	12626	15037	10347	13832	5966	64.5
**221**	MMV667487	C	G02	15433	18903	10673	17168	9302	44.6
**222**	MMV000356	C	G03	14356	17620	10777	15988	8122	51.6
**223**	MMV396705	C	G04	16681	19541	10926	18111	10245	39.0
**224**	MMV006704	C	G05	14550	18255	11935	16403	8537	49.2
**225**	MMV000760	C	G06	11849	14574	9294	13212	5346	68.2
**226**	MMV007881	C	G07	15389	18255	11055	16822	8956	46.7
**227**	MMV008212	C	G08	18188	19907	10442	19048	11182	33.4
**228**	MMV007363	C	G09	14143	19042	9637	16593	8727	48.0
**229**	MMV007791	C	G10	14802	15461	10827	15132	7266	56.7
**230**	MMV665843	C	G11	10944	15255	9917	13100	5234	68.8
**231**	MMV396595	C	H02	13492	18133	11061	15813	7947	52.7
**232**	MMV396669	C	H03	16472	18270	10266	17371	9505	43.4
**233**	MMV396704	C	H04	15482	19593	10332	17538	9672	42.4
**234**	MMV019762	C	H05	15311	14786	11255	15049	7183	57.2
**235**	MMV020505	C	H06	10362	12094	7154	11228	3362	80.0
**236**	MMV020942	C	H07	16111	14396	10407	15254	7388	56.0
**237**	MMV000839	C	H08	13532	19304	11230	16418	8552	49.1
**238**	MMV666599	C	H09	15674	20003	9614	17839	9973	40.6
**239**	MMV000481	C	H10	16009	19306	10887	17658	9792	41.7
**240**	MMV665897	C	H11	12002	14707	10290	13355	5489	67.3
**241**	MMV665908	D	A02	15088	13901		14495	6232	61.3
**242**	MMV665924	D	A03	13447	12323		12885	4623	71.3
**243**	MMV665944	D	A04	15450	14534		14992	6730	58.3
**244**	MMV666054	D	A05	14049	14395		14222	5960	63.0
**245**	MMV008474	D	A06	17267	17029		17148	8886	44.9
**246**	MMV000445	D	A07	10681	9962		10322	2059	87.2
**247**	MMV006882	D	A08	14878	14205		14542	6279	61.1
**248**	MMV007127	D	A09	14750	13029		13890	5627	65.1
**249**	MMV666123	D	A10	15237	14953		15095	6833	57.6
**250**	MMV006389	D	A11	15569	13291		14430	6168	61.7
**251**	MMV665949	D	B02	11080	10875		10978	2715	83.2

**252**	MMV665934	D	B03	14133	11860	12997	4734	70.6
**253**	MMV665994	D	B04	12694	10872	11783	3521	78.2
**254**	MMV665980	D	B05	12564	11741	12153	3890	75.9
**255**	MMV007577	D	B06	14760	12836	13798	5536	65.7
**256**	MMV019995	D	B07	11955	11059	11507	3245	79.9
**257**	MMV007208	D	B08	14692	12659	13676	5413	66.4
**258**	MMV000442	D	B09	15521	14249	14885	6623	58.9
**259**	MMV666124	D	B10	13754	12467	13111	4848	69.9
**260**	MMV000444	D	B11	12648	15123	13886	5623	65.1
**261**	MMV666109	D	C02	14843	15246	15045	6782	57.9
**262**	MMV665936	D	C03	14708	14413	14561	6298	60.9
**263**	MMV666020	D	C04	13118	11401	12260	3997	75.2
**264**	MMV665971	D	C05	15311	13890	14601	6338	60.7
**265**	MMV001241	D	C06	16040	13508	14774	6512	59.6
**266**	MMV000720	D	C07	12217	10975	11596	3334	79.3
**267**	MMV000619	D	C08	15065	14323	14694	6432	60.1
**268**	MMV006753	D	C09	15152	14116	14634	6372	60.5
**269**	MMV006787	D	C10	15020	15610	15315	7053	56.3
**270**	MMV665794	D	C11	14773	14471	14622	6360	60.6
**271**	MMV000917	D	D02	12083	12099	12091	3829	76.3
**272**	MMV666125	D	D03	12477	12512	12495	4232	73.7
**273**	MMV665987	D	D04	14191	12978	13585	5322	67.0
**274**	MMV007574	D	D05	15092	11562	13327	5065	68.6
**275**	MMV000326	D	D06	13521	12270	12896	4633	71.3
**276**	MMV000604	D	D07	16199	15186	15693	7430	53.9
**277**	MMV007557	D	D08	16936	15246	16091	7829	51.4
**278**	MMV000699	D	D09	14054	13876	13965	5703	64.6
**279**	MMV009127	D	D10	28936	25840	27388	19126	-18.6
**280**	MMV665786	D	D11	16659	14960	15810	7547	53.2
**281**	MMV006250	D	E02	13844	13596	13720	5458	66.1
**282**	MMV666079	D	E03	14214	11365	12790	4527	71.9
**283**	MMV665969	D	E04	11910	12260	12085	3823	76.3
**284**	MMV000304	D	E05	12381	10766	11574	3311	79.5
**285**	MMV000443	D	E06	16545	14134	15340	7077	56.1
**286**	MMV666604	D	E07	16827	14574	15701	7438	53.9
**287**	MMV008455	D	E08	12556	12417	12487	4224	73.8

**288**	MMV007199	D	E09	13719	11750	12735	4472	72.3
**289**	MMV085471	D	E10	17189	13613	15401	7139	55.7
**290**	MMV665797	D	E11	16657	12998	14828	6565	59.3
**291**	MMV006513	D	F02	13073	11911	12492	4230	73.8
**292**	MMV666095	D	F03	13519	11719	12619	4357	73.0
**293**	MMV665943	D	F04	11302	10122	10712	2450	84.8
**294**	MMV019555	D	F05	8290	8960	8625	363	97.7
**295**	MMV019741	D	F06	14903	11426	13165	4902	69.6
**296**	MMV019690	D	F07	10104	9154	9629	1367	91.5
**297**	MMV000621	D	F08	15866	15414	15640	7378	54.2
**298**	MMV008173	D	F09	17265	16225	16745	8483	47.4
**299**	MMV019241	D	F10	15580	15541	15561	7298	54.7
**300**	MMV665783	D	F11	16108	15453	15781	7518	53.4
**301**	MMV000787	D	G02	13081	12896	12989	4726	70.7
**302**	MMV666106	D	G03	16113	14763	15438	7176	55.5
**303**	MMV666022	D	G04	12597	13016	12807	4544	71.8
**304**	MMV498479	D	G05	13022	11724	12373	4111	74.5
**305**	MMV007396	D	G06	14335	13388	13862	5599	65.3
**306**	MMV000617	D	G07	15408	15492	15450	7188	55.4
**307**	MMV006764	D	G08	11258	12537	11898	3635	77.5
**308**	MMV007275	D	G09	12736	11152	11944	3682	77.2
**309**	MMV007273	D	G10	15769	14514	15142	6879	57.3
**310**	MMV666026	D	G11	15440	14772	15106	6844	57.6
**311**	MMV665923	D	H02	14496	13507	14002	5739	64.4
**312**	MMV666025	D	H03	14511	14141	14326	6064	62.4
**313**	MMV666060	D	H04	15849	14474	15162	6899	57.2
**314**	MMV666597	D	H05	16734	18254	17494	9232	42.7
**315**	MMV007224	D	H06	16064	14472	15268	7006	56.5
**316**	MMV020912	D	H07	21759	18397	20078	11816	26.7
**317**	MMV007181	D	H08	15972	16322	16147	7885	51.1
**318**	MMV007113	D	H09	13078	12101	12590	4327	73.2
**319**	MMV007228	D	H10	15603	15753	15678	7416	54.0
**320**	MMV665972	D	H11	18475	19958	19217	10954	32.1
**321**	MMV073843	E	A02	12312	12265	12289	5839	55.2
**322**	MMV006303	E	A03	9103	8501	8802	2352	81.9
**323**	MMV667490	E	A04	9851	9251	9551	3101	76.2

**324**	MMV667492	E	A05	11571	11867	11719	5269	59.5
**325**	MMV019881	E	A06	7229	6914	7072	622	95.2
**326**	MMV000478	E	A07	11027	11175	11101	4651	64.3
**327**	MMV020403	E	A08	11875	12113	11994	5544	57.4
**328**	MMV007764	E	A09	11903	12280	12092	5642	56.7
**329**	MMV665830	E	A10	6765	6869	6817	367	97.2
**330**	MMV665886	E	A11	9555	12625	11090	4640	64.4
**331**	MMV396664	E	B02	11875	11652	11764	5314	59.2
**332**	MMV001239	E	B03	11667	12051	11859	5409	58.4
**333**	MMV667491	E	B04	6802	6459	6631	181	98.6
**334**	MMV006169	E	B05	10122	10459	10291	3841	70.5
**335**	MMV086103	E	B06	11488	12059	11774	5324	59.1
**336**	MMV084434	E	B07	11049	11406	11228	4778	63.3
**337**	MMV666692	E	B08	11828	11988	11908	5458	58.1
**338**	MMV665836	E	B09	11426	11458	11442	4992	61.7
**339**	MMV665881	E	B10	11510	12155	11833	5383	58.7
**340**	MMV665875	E	B11	9575	9709	9642	3192	75.5
**341**	MMV007020	E	C02	11373	11943	11658	5208	60.0
**342**	MMV006656	E	C03	9494	9502	9498	3048	76.6
**343**	MMV396726	E	C04	11789	11971	11880	5430	58.3
**344**	MMV006522	E	C05	11571	11313	11442	4992	61.7
**345**	MMV666686	E	C06	11564	11326	11445	4995	61.6
**346**	MMV007474	E	C07	9228	10011	9620	3170	75.7
**347**	MMV006962	E	C08	9618	9794	9706	3256	75.0
**348**	MMV665813	E	C09	11198	11951	11575	5125	60.6
**349**	MMV080034	E	C10	11188	12051	11620	5170	60.3
**350**	MMV019199	E	C11	10987	11136	11062	4612	64.6
**351**	MMV396652	E	D02	12266	12601	12434	5984	54.0
**352**	MMV000704	E	D03	11263	11136	11200	4750	63.5
**353**	MMV396635	E	D04	11076	11561	11319	4869	62.6
**354**	MMV000986	E	D05	10436	10694	10565	4115	68.4
**355**	MMV008160	E	D06	11718	12181	11950	5500	57.8
**356**	MMV000753	E	D07	11199	11389	11294	4844	62.8
**357**	MMV011436	E	D08	11862	12063	11963	5513	57.7
**358**	MMV665882	E	D09	8935	10564	9750	3300	74.7
**359**	MMV665809	E	D10	10592	11136	10864	4414	66.1

**360**	MMV665810	E	D11	11352	12276	11814	5364	58.8
**361**	MMV665927	E	E02	11628	12102	11865	5415	58.4
**362**	MMV667486	E	E03	11725	12074	11900	5450	58.1
**363**	MMV667488	E	E04	11551	12142	11847	5397	58.5
**364**	MMV006825	E	E05	9981	10567	10274	3824	70.6
**365**	MMV009085	E	E06	10755	10877	10816	4366	66.5
**366**	MMV007591	E	E07	8706	8206	8456	2006	84.6
**367**	MMV128432	E	E08	11030	11616	11323	4873	62.6
**368**	MMV665852	E	E09	12753	15030	13892	7442	42.8
**369**	MMV396717	E	E10	11229	12072	11651	5201	60.1
**370**	MMV638723	E	E11	11418	12029	11724	5274	59.5
**371**	MMV396594	E	F02	12324	12622	12473	6023	53.7
**372**	MMV396665	E	F03	11669	12397	12033	5583	57.1
**373**	MMV396723	E	F04	9359	9836	9598	3148	75.8
**374**	MMV007654	E	F05	11810	12516	12163	5713	56.1
**375**	MMV011832	E	F06	11270	11477	11374	4924	62.2
**376**	MMV020750	E	F07	10885	11738	11312	4862	62.7
**377**	MMV007092	E	F08	9428	9693	9561	3111	76.1
**378**	MMV665898	E	F09	11622	11760	11691	5241	59.7
**379**	MMV665864	E	F10	9369	10896	10133	3683	71.7
**380**	MMV665814	E	F11	11197	12096	11647	5197	60.1
**381**	MMV007285	E	G02	11913	10916	11415	4965	61.9
**382**	MMV396663	E	G03	12176	12751	12464	6014	53.8
**383**	MMV007041	E	G04	11236	11837	11537	5087	60.9
**384**	MMV000340	E	G05	11738	11430	11584	5134	60.6
**385**	MMV666687	E	G06	10588	10157	10373	3923	69.9
**386**	MMV645672	E	G07	11557	11687	11622	5172	60.3
**387**	MMV011438	E	G08	10031	10929	10480	4030	69.0
**388**	MMV665840	E	G09	11825	12073	11949	5499	57.8
**389**	MMV665894	E	G10	12651	12665	12658	6208	52.3
**390**	MMV011522	E	G11	12171	11884	12028	5578	57.2
**391**	MMV020243	E	H02	10778	11193	10986	4536	65.2
**392**	MMV667489	E	H03	11862	11752	11807	5357	58.8
**393**	MMV396770	E	H04	12027	12056	12042	5592	57.0
**394**	MMV009015	E	H05	10410	11589	11000	4550	65.1
**395**	MMV008829	E	H06	11279	11522	11401	4951	62.0

**396**	MMV011895	E	H07	10855	10841	10848	4398	66.2
**397**	MMV666689	E	H08	11850	11199	11525	5075	61.0
**398**	MMV665824	E	H09	11220	12121	11671	5221	59.9
**399**	MMV665812	E	H10	10815	11181	10998	4548	65.1
**400**	MMV001041	E	H11	11290	12376	11833	5383	58.6
