# Supplementary material for: Gametocytocidal Screen Identifies Novel Chemical Classes with Plasmodium falciparum Transmission Blocking Activity
Source: PLoS One. 2014 Aug 26;9(8):e105817. doi: 10.1371/journal.pone.0105817 (PMC4144897; doi:10.1371/journal.pone.0105817)
Supplement: Table S5 — Compilation of top MMV malaria box hits from four gametocyte assays with three different reporters compared to the SYBR Green assay hits. (PDF) [file pone.0105817.s005.pdf]

Table S5. Compilation of top MMV Malaria Box hits from four gametocyte assays with three different reporters compared to the SYBR Green assay hits.

	SYBR Green I (SD)	Confocal Fluorescence Microsopy		Alamar Blue	Luciferase
Gametocyte stage	Late	Early	Late	Early/Late	Early
MMV#	IC$_{50}$ nM				
MMV000448	1083 (575)	1439	703	1000 (1866)	
MMV006172	2590 (320)	1113	1364	420 (1482)	
MMV007591	5370 (1030)	to test	1091	1150 (2635)	
MMV019555	3390 (480)	1428	100% at 5 µM	470 (4686)	
MMV019918	890 (330)	824	692	320 (1866)	
MMV000445	50% at 10 µM	75% at 5 µM	1611	7427	
MMV006303	2100 (650)	1710	N.D.	2635	
MMV019690	30% at 10 µM	100% at 5 µM	1166	6619	
MMV665830	3330 (640)	100% at 5 µM	541	1633	
MMV665941	1780 (40)	769	315	5899	
MMV667491	4460 (1520)	N.D.	1060	2635	
MMV396794	8190 (940)			870 (2957)	
MMV000848	3570 (3160)			2093	
MMV019780	3820 (660)			4176	
MMV019881	5510 (940)			2957	
MMV020505	6640 (1090)			3317	
MMV396797	8830 (1160)			6619	
MMV665878	5420 (1400)			2635	
MMV000248		1444	1091	310 (1482)	
MMV007224		100% at 5 µM	948	1980 (20931)	
MMV007384		949	1114	870 (37221)	
MMV011438		80% at 5 µM	1129.5	820 (5258)	
MMV019266		1142	324	520 (11770)	
MMV665794		1306	416	680 (18655)	
MMV665943		100% at 5 µM	1099	570 (29566)	

MMV666125		1157	745	480 (833)	
MMV666693		NA	438	730 (NA)	
MMV666079				1460 (16626)	
MMV666686				2060 (NA)	
MMV666687				1660 (14818)	
MMV006087		1020	N.D.	372	249.3 (10.7)
MMV007695		NA	826.5	NA	>5 µM
MMV020500		274	310	235	252.4 (5.0)
MMV020788		891	415	105	197.0 (7.0)
MMV665782		823	276	209	213.7 (7.9)
MMV006457				NA	< 5 µM
MMV006706				NA	220.0 (9.9)
MMV008138				NA	>5 µM
MMV020492				NA	>5 µM
MMV665805				4176	80% at 5 µM
MMV665874				NA	>5 µM
MMV000442		100% at 5 µM	920	332	
MMV000570		100% at 5 µM	1270	469	
MMV000634		1790	35% at 5 µM	10490	
MMV000642		100% at 5 µM	596	10490	
MMV000753		699	70% at 5 µM	18655	
MMV000787		100% at 5 µM	584	8333	
MMV000963		1476	90% at 5 µM	9350	
MMV001041		N.D.	1350	14818	
MMV001246		100% at 5 µM	781	11770	
MMV006309		NA	936	NA	
MMV006389		NA	733	NA	
MMV006787		100% at 5 µM	1170	2635	
MMV007041		N.D.	673	NA	

MMV007116		N.D.	253	NA	
MMV007127		NA	875	NA	
MMV007160		1185	208	NA	
MMV007181		100% at 5 μM	784	590	
MMV007907		461	231	7427	
MMV009063		100% at 5 μM	897	2349	
MMV011795		100% at 5 μM	1321	5899	
MMV019406		1390	676	418	
MMV084434		NA	892	NA	
MMV084940		100% at 5 μM	1040	1321	
MMV396594		N.D.	1290	NA	
MMV396595		N.D.	1590	NA	
MMV403679		N.D.	1090	18655	
MMV665785		712	382	148	
MMV665786		1308	70% at 5 μM	14818	
MMV665820		1214	75% at 5 μM	3722	
MMV665827		NA	337	NA	
MMV665831		810	371	7427	
MMV665841		100% at 5 μM	591	662	
MMV665875		588	25% at 5 μM	372	
MMV665882		252	63	2957	
MMV665971		359	228	2957	
MMV665977		NA	574	NA	
MMV665987		NA	759	NA	
MMV666021		NA	603	NA	
MMV666023		100% at 5 μM	184	10490	
MMV666054		100% at 5 μM	1075	16626	
MMV666061		100% at 5 μM	899	1663	
MMV666101		NA	333	NA	

MMV666116		1360	673	662	
MMV666596		NA	592	NA	
MMV666597		819	465	2093	
MMV666604		80% at 5 µM	1083	7427	
MMV666692		830	594	29566	

NA = not active

Duffy S, Avery VM (2013) Identification of inhibitors of *Plasmodium falciparum* gametocyte development. Malar J 12: 408.

Bowman JD, Merino EF, Brooks CF, Striepen B, Carlier PR, et al. (2013) Anti-apicoplast and gametocytocidal screening to identify the mechanisms of action of compounds within the Malaria Box.

Sun W, Tanaka TQ, Magle CT, Huang W, Southall N, et al. (2014) Chemical signatures and new drug targets for gametocytocidal drug development. Sci Rep 4: 3743.

Identification of MMV Malaria Box Inhibitors of *Plasmodium falciparum* Early-Stage Gametocytes Using a Luciferase-Based High-Throughput Assay. Antimicrob Agents Chemother 57: 6050–6062.
