# Supplementary material for: Gametocytocidal Screen Identifies Novel Chemical Classes with Plasmodium falciparum Transmission Blocking Activity
Source: PLoS One. 2014 Aug 26;9(8):e105817. doi: 10.1371/journal.pone.0105817 (PMC4144897; doi:10.1371/journal.pone.0105817)
Supplement: Table S6 — FDA drug library SYBR Green I fluorescence data and analysis. (PDF) [file pone.0105817.s006.pdf]

Table S6. FDA Drug Library SYBR Green I fluorescence data and analysis					
**name**	**indication**	**sybr 1**	**sybr 1-μc**	**% inh**	**% inh cutoff**
melphalan	antineoplastic	6477	-2778	150.8	100.0
gentian violet	antiseptic	5010	-3724	148.2	100.0
homidium bromide, homidium bromide (ethidium bromide)	anthelminithic	3484	-4591	148.1	100.0
ifosfamide	antineoplastic	7305	-1950	135.7	100.0
pentamidine	antiprotozoal	6386	-1750	129.1	100.0
thonzonium, thonzonium bromide	antiseptic	7416	-840	113.2	100.0
cetalkonium chloride, cetalkonium chloride (benzyldimethylhexadecyl ammonium chloride)	antibacterial	7414	-722	112.0	100.0
benzethonium chloride	antiseptic	7812	-922	111.9	100.0
cetylpyridinium bromide monohydrate (no monohydrate), cetylpyridinium bromide monohydrate	antiseptic	7604	-652	110.3	100.0
benzalkonium chloride	antiseptic	8058	-676	108.7	100.0
methylbenzethonium chloride	antiseptic	7753	-503	107.9	100.0
parthenolide		7449	-520	106.7	100.0
piperine	antibacterial	7733	-342	103.6	100.0
pyrvinium pamoate	anthelminithic	7942	-194	103.2	100.0
maprotiline, maprotiline hydrochloride	antidepressant	9726	-101	101.9	100.0
anastrozole	antineoplastic	9155	-100	101.8	100.0
cetylpyridinium, cetylpyridinium chloride, cetylpyridinium (pyrisept)	antiseptic	8307	51	99.2	99.2
benzododecinium chloride, benzododecinium chloride (benzyl-dimethyl-dodecylammonium-chloride)	antiseptic	7808	165	97.7	97.7

tilorone, tilorone hydrochloride, tilorone dihydrochloride	antiviral	7813	170	97.6	97.6
dithiazanine iodide, dithiazanine iodide (3,3 diethylthiadicarbocyanine iodide)	anthelminithic	8441	305	94.9	94.9
olsalazine, olsalazine sodium	anti-inflammatory (gastrointestinal), antiinflammatory	10042	314	93.2	93.2
pyrithione, pyrithione zinc, pyrithione zinc (1-hydroxypyridine-2-thione zinc salt), 2-mercaptopyridine n-oxide	antibacterial	8554	418	93.0	93.0
aminopyrine, amidopyrine, aminopyrine (4 dimethylamino antipyrene)	antiinflammatory	10163	435	90.6	90.6
antimony potassium tartrate, potassium antimonyl tartrate trihydrate, 99+%	anthelminithic	8353	710	90.2	90.2
hydroxypropyl cellulose (hyprolose)		10594	866	81.4	81.4
protriptyline, protriptyline hydrochloride	antidepressant	10845	1018	81.2	81.2
chloroxine, chloroxine (5,7-dichloro-8-hydroxyquinoline)	dermatologic	10646	918	80.2	80.2
piperacetazine	antipsychotic	10995	1168	78.4	78.4
medroxyprogesterone acetate	progestogen	9695	1443	78.1	78.1
beclomethasone	glucocorticoid	10765	1037	77.7	77.7
disulfiram, disulfiram (tetraethylthiuram disulfide)	alcohol deterrant	9527	1098	77.1	77.1
ammonium bromide, ammonium bromide 40gr/fl oz	sedative	10833	1060	76.5	76.5

ethylamine salicylate 0.5%, ethylamine	dermatologic	10834	1106	76.2	76.2
esculin unk, esculin, esculin hydrate, aesculin, esculin monohydrate	dermatologic	10845	1117	75.9	75.9
primaquine, primaquine phosphate, primaquine diphosphate	antimalarial	9582	1446	75.9	75.9
betamethasone acetate, betamethasone	glucocorticoid	10870	1142	75.4	75.4
leflunomide	antirheumatic	10877	1149	75.3	75.3
phenformin, phenformin hydrochloride	antidiabetic	9927	1675	74.6	74.6
sodium fluoride	bone resorption inhibitor	9927	1675	74.6	74.6
prazosin, prazosin hydrochloride	antihypertensive	9837	1540	74.5	74.5
phentolamine, phentolamine hydrochloride, phentolamine methane-sulfonate	antihypertensive	9879	1582	73.8	73.8
gallium, gallium nitrate, gallium (iii) nitrate hydrate	antineoplastic	10704	1450	73.5	73.5
roxithromycin	antibiotic	9587	1944	73.1	73.1
methylthiouracil, methylthiouracil (6-methyl-2-thiouracil)	thyroid	10058	1806	72.6	72.6
anazolene sodium, anazolene sodium (acid blue 92)	diagnostic aid	9524	1893	72.4	72.4
dacarbazine	antineoplastic	10776	1522	72.2	72.2
meloxicam, meloxicam sodium	antiinflammatory	11024	1296	72.1	72.1
mebutamate, mebutamate (2-methyl-2-(1-methylpropyl)-1,3-propanediol dicarbamate)	antihypertensive	9989	1692	72.0	72.0
flutamide	antineoplastic	10810	1556	71.5	71.5

methyldopa, methyldopa (l,-), methyl-dopa (alpha-methyl-l-beta-3,4-dihydroxyphenylalanine)	antihypertensive	10041	1744	71.1	71.1
risedronate, risedronic acid, risedronate sodium	bone resorption inhibitor., bone resorption inhibitor	10224	1972	70.1	70.1
vasopressin, vasopressin ([arg8]-vasopressin)	antihypotensive	10140	1843	69.5	69.5
picotamide, picotamide monohydrate	antithrombotic	9745	2114	69.1	69.1
metformin, metformin hydrochloride, metformin (1,1-dimethylbiguanide hydrochloride)	antidiabetic	10166	1869	69.0	69.0
climbazole	antifungal	9893	2250	68.8	68.8
terfenadine	antihistaminic	9818	2057	68.7	68.7
megestrol acetate	progestogen	10321	2069	68.7	68.7
aminosalicylic acid, 5 aminosalicylic acid, 4-aminosalicylic acid, p-aminosalicylic acid	antibacterial	9819	1341	68.6	68.6
physostigmineserineserine sulfate, physostigmine sulfate, physostigmine salicylate, physostigmine sulfateserine (physostigmine)	cholinergic	10461	2385	67.8	67.8
phenylphenol, o- 1%, phenylphenol, 2-phenylphenol	antiseptic	10307	2051	67.6	67.6
ergoloid mesylates, ergoloid mesylate	antihypertensive	10252	1955	67.6	67.6
medrysone, medrysone (6-alpha-methyl-11-beta-hydroxyprogesterone)	glucocorticoid	11236	1508	67.5	67.5
salicylic acid	analgesic, dermatologic	9868	1390	67.4	67.4

acarbose	cardiotonic, antidiabetic	10178	2176	67.4	67.4
raloxifene, raloxifene hydrochloride	bone resorption inhibitor	10460	2208	66.5	66.5
alitretinoin, isotretinoin, neovitamin a, retinoic acid, tretinoin, 9-cis-retinoic acid, 13-cis-retinoic acid, isotretinon, tretinon, alitretinoin (9-cis-retinoic acid), tretinoin (retinoic acid, all trans), neovitamin a (13-cis-retinal)	dermatologic, antineoplastic, vitamin	11282	1554	66.5	66.5
acetomenaphthone	phamaceutic aid	10694	2841	66.2	66.2
guanfacine, guanfacine hydrochloride, guanidine hydrochloride	antihypertensive	10338	2041	66.2	66.2
xanthinol niacinate, xanthinol niacinate (xanthinol nicotinate)	vasodilator	10014	2383	65.2	65.2
carteolol, carteolol hydrochloride, carvedilol	antihypertensive	10424	2127	64.7	64.7
bismuth sodium triglycollamate, bismuth sodium triglycollamate (nitrilotriacetic acid)	immunomodulator	10057	2426	64.6	64.6
phosphocreatine	cardotonic	10068	2437	64.4	64.4
21-acetoxypregnenolone potency not given, 21-acetoxypregnenolone	antiinflammatory	11389	1661	64.2	64.2
pantethine, pantethine (d-pantethine)	antihyperlipidemic	10142	2511	63.3	63.3
azaribine, azaribine (6-azauridine 2',3',5'-triacetate)	dermatologic	11445	1717	63.0	63.0
salicylamide	analgesic	10062	1584	62.9	62.9
carvedilol	antihypertensive	10555	2258	62.6	62.6

cabufocon (cellulose acetate butyrate)		10214	2583	62.3	62.3
trolamine, triethanolamine, trolamine (triethanolamine hydrochloride)	analgesic, dermatologic	10092	1614	62.2	62.2
guanethidine, guanethidine monosulfate, guanethidine sulfate	antihypertensive	10604	2307	61.8	61.8
verapamil, dexverapamil, verapamyl hydrochloride, verapamil hydrochloride	antihypertensive, antianginal	10628	2331	61.4	61.4
monensin, monensin sodium salt, monensin sodium (monensin a is shown), monensin sodium	antibiotic	11764	3690	61.4	61.4
polyoxyl 10 oleyl ether, polyoxyl 10 oleyl ether brij 92	phamaceutic aid	10283	2652	61.3	61.3
puromycin, puromycin dihydrochloride, puromycin hydrochloride	antineoplastic	12069	3601	61.0	61.0
lomerizine, lomerizine hcl	antimigraine	10320	2689	60.7	60.7
1-pentanol	dermatologic	10338	2707	60.5	60.5
methoxsalen, methoxy-8-psoralen, methoxsalen (8-methoxypsoralen)	dermatologic	11569	1841	60.3	60.3
lindane, lindane (1,2,3,4,5,6-hexachlorocyclohexane,_-isomer), b-hch pestanal	dermatologic	11574	1846	60.2	60.2

propranolol, dexpropranolol, (s)-propranolol, dexpropranolol hydrochloride, propranolol hydrochloride, propranolol hydrochloride'(r)-, propranolol hydrochloride'(s)-, propranolol hydrochloride (+/-)	antihypertensive, antiarrhythmic	10722	2425	59.8	59.8
tenatoprazole	antihypertensive	10394	2763	59.7	59.7
tripelennamine, tripelennamine citrate	antihistaminic	10412	2651	59.6	59.6
oxiniacate olamine, oxiniacic acid, oxiniacic acid (nicotinic acid n-oxide)	antihyperlipidemic	10405	2774	59.5	59.5
buflomedil, buflomedil hydrochloride	vasodilator	10464	2833	58.6	58.6
oxedrine, synephrine'(+,-)-, oxedrine (synephrine)	antihypotensive	10499	2868	58.1	58.1
bamethan, bamethan sulfate, bamethan sulfate (bamethane)	vasodilator	10537	2906	57.6	57.6
phenyl salicylate	analgesic	10307	1829	57.1	57.1
spiramycin	antibiotic	12200	4126	56.8	56.8
benoxinate, benoxinate hydrochloride	anesthetic	10327	1849	56.6	56.6
triprolidine, triprolidine hydrochloride, triprolidine (trans-triprolidine hydrochloride)	antihistaminic	10623	2862	56.4	56.4
fendiline, fendiline hydrochloride, fendiline (3,3-diphenyl-n-1-propanamine)	vasodilator	10619	2988	56.4	56.4

bucladesine, bucladesine sodium salt, bucladesine sodium (n6, o-2-dibutyryladenosine-3',5'-cyclic-monophosphate sodium salt)	cardiotonic	10627	2996	56.3	56.3
methdilazine, methdilazine hydrochloride	dermatologic	11770	2042	56.0	56.0
monoethanolamine, monoethanolamine (ethanolamine)	phamaceutic aid	10676	3045	55.5	55.5
clotrimazole	antifungal	10870	2680	55.4	55.4
camphor water qs, camphor, camphor (1r), 1s-camphor, camphor, (1r)-(+)-, camphor white oil	analgesic, therapeutic plant extract	10385	1907	55.3	55.3
propofol, propofol (2,6-diisopropylphenol)	anesthetic	10399	1921	55.0	55.0
nitroxoline, nitroxoline (8-hydroxy 5-nitroquinoline)	antibiotic	10917	3274	54.7	54.7
malathion	ectoparasiticide, dermatologic	11840	2112	54.5	54.5
desipramine, desipramine hydrochloride	antidepressant	12332	2505	53.7	53.7
g-aminobutyric acid, piperidic acid, gamma-aminobutyric acid, aminobutyricacid(gaba), gaba (gamma-amino-n-butyric acid), 4-aminobutyric acid (gaba)	antihypertensive, nootropic	10804	3173	53.7	53.7
magnesium peroxide 3/16gr, magnesium peroxide	antiseptic	11965	2411	53.6	53.6
chromocarb, chromocarb (chromone-2-carboxylic acid 4-oxo-4h-1-benzopyran-2-carboxylic acid)	capillary protectant	12816	4348	52.9	52.9
acetaminophen	analgesic	10493	2015	52.8	52.8

ketaminesketamine, ketamine hydrochloride, ketamine/xylazine	anesthetic, sedative	10519	2041	52.1	52.1
ditiocarb, diethyldithiocarbamate, ditiocarb sodium, ditiocarb (diethyldithiocarbamic acid sodium, thiocarb)	immunomodulator	12886	4418	52.1	52.1
pempidine, pempidine (1,2,2,6,6-pentamethylpiperidine)	antihypertensive	10909	3278	52.1	52.1
aspartame	pharmaceutical aid, phamaceutic aid	10909	3278	52.1	52.1
camphor water qs, camphor, camphor (1r), 1s-camphor, camphor, (1r)-(+)-, camphor white oil	analgesic, therapeutic plant extract	10531	2053	51.9	51.9
antimycin antimycin a (a1 shown)		11753	3784	51.5	51.5
pentetate calcium trisodium, pentifylline	antiamebic, vasodilator	10982	3351	51.1	51.1
hydrochlorothiazide	diuretic	12768	4694	50.8	50.8
storax		12815	4447	50.3	50.3
nifuroxazide	antibiotic	11922	3953	49.4	49.4
procodazole, procodazole (2-benzimidazolepropionic acid)	misc-immunomodulator, immunomodulator	13151	4683	49.3	49.3
tramadol, tramadol hydrochloride	analgesic	10667	2189	48.7	48.7
podophyllum resin	dermatologic	11638	4097	48.5	48.5
clobetasone butyrate	glucocorticoid	13223	4755	48.5	48.5
aminacrine, aminacrine hydrochloride, aminacrine (9-aminoacridine)	antiseptic	11533	3277	48.3	48.3
ranolazine, ranolazine dihydrochloride	antianginal	11284	3653	46.7	46.7

tioxolone, tioxolone (6-hydroxy-1,3-benzoxathiol-2-one)	dermatologic	13144	4776	46.6	46.6
phloridzin	n/a	12228	3046	45.8	45.8
isoxsuprine, isoxsuprine hydrochloride	vasodilator	13250	5176	45.8	45.8
metyrapone, metyrapone (2-methyl-1,2-di-3-pyridyl 1-propanone)	diagnostic aid	11352	3888	45.7	45.7
eserolineseroline fumarate salt'(-)-	n/a	12247	3065	45.5	45.5
piracetam	nootropic	13317	5243	45.1	45.1
ferricgluconate, ferric gluconate	hematinic, vitamin	11957	4416	44.5	44.5
vitamin k3, menadione, aminosidine	vitamin	12068	4527	43.1	43.1
cinchophen, cinchophen (2-phenyl 4-quinoline carboxylic acid)	analgesic	12326	4250	42.7	42.7
propafenone, propafenone hydrochloride	antiarrhythmic	11823	3821	42.7	42.7
azelastine, azelastine hydrochloride, azelastine hcl	antihistaminic	11578	3817	41.9	41.9
mequinol, mequinol (4-methoxyphenol, 99%)	dermatologic	13595	5227	41.5	41.5
samarium, samarium iii acetate, samarium(iii) acetate hydrate	antineoplastic	12532	3278	40.0	40.0
desloratadine	antihistaminic	11704	3943	40.0	40.0
tricaprilin, tricaprilin (glyceryl trioctanoate)	nutrient, unclassified	13784	5416	39.4	39.4
bucetin	analgesic	12575	4499	39.3	39.3
drofenine, drofenine hydrochloride	antispasmodic	13958	5884	38.4	38.4
alfadex	erectile dysfunction	13137	5284	37.2	37.2
menadiol sodium diphosphate	vitamin (prothrombogenic) ., vitamin	12544	5003	37.1	37.1

carotenoids, vitamin a, retinol, retinol all-trans, retinyl acetate, vitamin a (acetate), vitamin a acetate (all trans-retinol acetate)	vitamin	12584	5043	36.6	36.6
chlorquinaldol, chlorquinaldol (5,7-dichloro-2-methyl-8-quinolinol)	antibacterial	11954	3818	36.5	36.5
olive oil		12868	3314	36.2	36.2
menadione sodium bisulfite, menadione sodium bisulfate	vitamin	12640	5099	35.9	35.9
clioquinol	antibacterial	12008	3872	35.6	35.6
resorcinol	diagnostic aid	14227	6153	35.6	35.6
ammonium magnesium phosphate hydrate	vitamin	12694	5153	35.3	35.3
potassium hydroxide	dermatologic	12697	5156	35.2	35.2
paromomycin, paromomycin sulfate	antibiotic	13736	5003	35.2	35.2
amaranth	therapeutic plant extract	13309	5456	35.2	35.2
cholecalciferol, cholecalciferol (vitamin d3)	vitamin (antirachitic), vitamin	12703	5162	35.1	35.1
epirizolepirizole (mepirizole)	analgesic	12922	4846	34.6	34.6
feredetate, sodium feredetate	hematinic	14229	5861	34.4	34.4
naringenine, naringenin		13121	5152	34.0	34.0
ouabain	cardiotonic	14383	6309	33.9	33.9
dimethyl sulfoxide	urologic	11620	3191	33.5	33.5
chlorthalidone	diuretic	12456	4454	33.2	33.2
ferric sulfate heptahydrate		11639	3210	33.1	33.1
myo inositol, inositol, myo-inositol, scyllo inositol	vitamin, vitamin b complex, n/a	12869	5328	33.1	33.1

etofylline, hydroxyethyltheophyllineto fylline (7-(_-hydroxyethyl)theophylline) , 7-hydroxyethyltheophylline	bronchodilator	14385	6017	32.7	32.7
bergenin, bergenin monohydrate	antiulcerative	13519	5666	32.7	32.7
arginine glutamate, l-arginine l-glutamate salt	antidote	12924	5383	32.4	32.4
acetic acid	nutrient, dermatologic	12942	5401	32.1	32.1
benfotiamine, benfotiamine (s-benzoylthiamine o-monophosphate)	vitamin (enzyme cofactor), vitamin	14462	6094	31.8	31.8
alitretinoin, isotretinoin, neovitamin a, retinoic acid, tretinoin, 9-cis-retinoic acid, 13-cis-retinoic acid, isotretinon, tretinon, alitretinoin (9-cis-retinoic acid), tretinoin (retinoic acid, all trans), neovitamin a (13-cis-retinal)	dermatologic, antineoplastic, vitamin	12973	5432	31.8	31.8
allantoic acid	nutrient	12981	5440	31.7	31.7
carotenoids, vitamin a, retinol, retinol all-trans, retinyl acetate, vitamin a (acetate), vitamin a acetate (all trans-retinol acetate)	vitamin	12993	5452	31.5	31.5
gallic acid	antiseptic	13116	3562	31.4	31.4
penimepicycline	antibiotic	12605	4962	31.3	31.3
nimesulide	antiinflammatory	14812	6344	31.3	31.3
rifamixin, rifaximin	antibiotic, antibacterial	12620	4977	31.1	31.1

tracazolate, tracazolate hydrochloride	sedative	13205	5129	30.8	30.8
dibutyl phthalate, butyl phthalate	insecticide	11753	3324	30.8	30.8
glafenine, glafenine hydrochloride	analgesic	13230	5154	30.5	30.5
indium, indium chlorides in 113m	vitamin, diagnostic aid	13082	5541	30.4	30.4
bromofos	antineoplastic	13717	5864	30.3	30.3
methazolamide	diuretic	12651	4649	30.3	30.3
dexindoprofen, indoprofen	analgesic	13249	5173	30.2	30.2
bisacodyl	laxative	14752	6678	30.1	30.1
hexestrol	estrogen	14763	6689	30.0	30.0
butaclamol, butaclamol hydrochloride	antipsychotic	13757	5904	29.9	29.9
monobenzone, monobenzone (4-(benzyloxy)phenol)	dermatologic	13124	5583	29.9	29.9
alanine, alanine, d, l (dl-alpha-alanine), l-alanine also 785	nutrient	13134	5593	29.7	29.7
thalidomide	immunomodulator	11804	3375	29.7	29.7
acetylcholine, acetylcholine chloride	cholinergic	14822	6748	29.3	29.3
amoxicillin	antibiotic	14230	5497	28.8	28.8
butylated hydroxytoluene, 2,6-di-t-butyl-4-methylphenol (bht)	phamaceutic aid	13853	6000	28.7	28.7
bromodiphenhydramine, bromodiphenhydramine hydrochloride	antihistaminic	12444	4683	28.7	28.7
crotamiton, crotamiton (n-ethyl-o-crotonotoluidine)	dermatologic	13051	3323	28.4	28.4
pyrethrins	insecticide	11885	3456	28.0	28.0
bile salts		13276	5735	27.9	27.9
penicillin g sodium	antibiotic	12826	4596	27.9	27.9
moexipril, moexipril hydrochloride	antihypertensive	12653	4356	27.8	27.8
ammonium lactate	nutrient	13289	5748	27.8	27.8

benserazide		13433	5357	27.7	27.7
carbocysteine, carbocysteine (s-carboxymethyl-l-cysteine)	mucolytic	15143	6675	27.7	27.7
bromoform	sedative	13441	5365	27.6	27.6
meprobamate	sedative	13746	3919	27.6	27.6
lactose monohydrate, lactose, alpha-d-lactose monohydrate, lactose (4-o-beta-galactopyranosyl-d-glucose)	nutrient	13311	5770	27.5	27.5
ferric phosphate	vitamin	13319	5778	27.4	27.4
copper, copper (ii) sulfate, copper (ii) acetate	antidote to phosphorus	12674	5210	27.3	27.3
dodecylamine, dodecylamine lactate 0.40%	antiseptic	11935	3506	27.0	27.0
alfafosfalin		14022	6169	26.7	26.7
digoxin	cardiotonic	14401	5668	26.6	26.6
aminopyrine, amidopyrine, aminopyrine (4 dimethylamino antipyrene)	antiinflammatory	14042	6189	26.5	26.5
thiostrepton	antibiotic	12953	5310	26.5	26.5
carbaril, carbaryl (carbaril)	ectoparasiticide	15264	6796	26.4	26.4
ornithine, l-ornithine, ornithine a-ketoglutarate	antidote, nutrient	14965	6597	26.2	26.2
sodium glucuronate, sodium glucuronate (d-glucuronic acid)	nutrient	14974	6606	26.1	26.1
propidium iodide		13746	5777	26.0	26.0
tioconazole	antifungal	12633	4443	26.0	26.0
cod liver oil		13457	5916	25.7	25.7
magnesium phosphate dibasic trihydrate	laxative	13419	3865	25.6	25.6
ethoxzolamidethoxzolamide (6-ethoxy-2-benzothiazolesulfonamide)	diuretic	12967	4965	25.5	25.5
choline, choline chloride	cholinergic	15202	7128	25.4	25.4

creatinine, creatine, creatine phosphate disodium salt tetrahydrate	nutrient	13803	5834	25.3	25.3
nicotinic acid, niacin, nicotinic acid (niacin)	dermatologic, vitamin	13501	5960	25.1	25.1
ibuprofen, dexibuprofen, ibuprofen's-(+)-		13503	5962	25.1	25.1
phenolsulfonphthalein, phenolsulfonphthalein (phenol red free acid)	diagnostic aid	12840	5376	25.0	25.0
tetracycline, tetracycline hydrochloride	antibiotic	13012	4756	25.0	25.0
mecobalamin, mecobalamin (methylcobalamin)	vitamin	15096	6728	24.7	24.7
chlorophenothane, dichlorodiphenyltrichloreth ane	misc-insecticidectopara siticide	12044	3615	24.7	24.7
camylofin, calcium phosphate	antispasmodic	14191	6338	24.7	24.7
metaproterenol, metaproterenol polistirex, metaproterenol sulfate, metaproterenol sulfate, orciprenaline sulfate	bronchodilator	14550	5817	24.7	24.7
abamectin	anthelminithic	14200	6347	24.6	24.6
clofoctol	antibiotic	13094	5451	24.5	24.5
butopyronoxyl	insecticide	12070	3641	24.2	24.2
vitamin e acetate 10mg, alpha-tocopherol acetate, vitamin e acetate, alpha-tocopherol acetate 25mg, alpha-tochopheryl acetate, alpha-tochopheryl acetate (vitamin e), vitamin e acetate (±)-_-tocopherol acetate	vitamin	13583	6042	24.1	24.1
sparfloxacin	antibiotic	13081	4851	23.9	23.9

bacto yeast extract		13606	6065	23.8	23.8
bile bovine		13606	6065	23.8	23.8
ethacrynic acid	diuretic	15353	7279	23.8	23.8
chlorazanil, chlorazanil hydrochloride, chlorazanil (l-mimosine)	antifungal	13921	5952	23.7	23.7
emetinemetine hydrochloridemetine dihydrochloride	antiamebic	12091	3662	23.7	23.7
alitretinoin, isotretinoin, neovitamin a, retinoic acid, tretinoin, 9-cis-retinoic acid, 13-cis-retinoic acid, isotretinon, tretinon, alitretinoin (9-cis-retinoic acid), tretinoin (retinoic acid, all trans), neovitamin a (13-cis-retinal)	dermatologic, antineoplastic, vitamin	13632	6091	23.5	23.5
phosphate dodecahydrate, phosphatedodecahydrate, sodium phosphate dodecahydrate	laxative	12951	5487	23.4	23.4
beef extract powder		13644	6103	23.3	23.3
butetamate, butetamate (butethamate)	antitussive	15224	6856	23.3	23.3
cycloheximide	antineoplastic	15549	7081	23.3	23.3
protamine chloride, grade v, protamine sulfate		12975	5511	23.1	23.1
nitrofurantoin	antibiotic	13135	4905	23.0	23.0
hexadimethrine bromide, hexadimethrine bormide	antidote	12987	5523	22.9	22.9
picric acid 0.057%, trinitrophenol, picric acid	antiseptic, dermatologic	13682	6141	22.8	22.8
bromociclen	unclassified	14351	6498	22.8	22.8
methyl salicylate	dermatologic	13702	6161	22.6	22.6
rotenone, rotenone 2%	antibacterial	15491	7417	22.3	22.3
carbidopa	antiparkinsonian	14736	6003	22.3	22.3
dihydroergotamine tartrate		14054	6085	22.0	22.0
mepartricin	antifungal	13273	5630	22.0	22.0

vitamin k3, menadione, aminosidine	vitamin	13760	6219	21.9	21.9
haloperidol metabolite ii		13873	5797	21.8	21.8
hycanthone	anthelminithic	13320	5677	21.4	21.4
doxapram, doxapram hydrochloride	respiratory stimulant	12204	3775	21.4	21.4
cefotaxime, cefotaxime sodium, cefotaxime sodium salt	antibiotic	14820	6087	21.2	21.2
cerous oxalate 0.5gr, cerium oxalate, cerous oxalate	antiemetic	15425	7057	21.1	21.1
amsacrine	antineoplastic	15755	7287	21.1	21.1
edetate sodium, edetatedta, ethylenediaminetetraacetic acid, calcium disodium salt hydratethylenediaminetetraacetic acidisodium salt dihydrate	antidote	13126	5662	21.0	21.0
oxytetracycline, oxytetracycline dihydrate, oxytetracycline hemicalcium salt	antibiotic	13272	5042	20.9	20.9
acetazolamide	diuretic, antiglaucoma	13278	5276	20.8	20.8
dibekacin	antibiotic	13366	5723	20.8	20.8
denatonium benzoate	phamaceutic aid	14155	6186	20.8	20.8
oxantel, oxantel pamoate	anthelminithic	13395	5752	20.4	20.4
butacetin, bupropion	analgesic	14587	6734	20.0	20.0
tetradecylsulfate, sodium tetradecyl sulfate, tetradecyl sulfate, sodium salt, 95%	antiseptic	13330	5074	19.9	19.9
astemizole	antihistaminic	13022	5261	19.9	19.9
pentoxifylline 400mg, pentoxifylline, pentixifylline 400mg	anticoagulant, bronchodilator	15740	7666	19.7	19.7

butoxyphenylacethydroxamate, bufexamac, phenylacetohydroxamic acid (bufexamac)	antiinflammatory	15882	7414	19.7	19.7
vitamin a palmitate, vitamin a palmitate eq 25,000 units base, retinyl palmitate, vitamin a palmitate (retinol palmitate)	vitamin	13937	6396	19.6	19.6
oxychlorosene	antiseptic	13349	5093	19.6	19.6
aluminum sulfate	antiseptic	14619	6766	19.6	19.6
myo inositol, inositol, myo-inositol, scyllo inositol	vitamin, vitamin b complex, n/a	13939	6398	19.6	19.6
streptomycin sulfate	antibacterial, antibiotic	12969	4833	19.6	19.6
histamine, histamine dihydrochloride	antineoplastic	13662	4408	19.3	19.3
closantel	anthelminithic	13480	5837	19.2	19.2
nalidixic acid, nalidixic acid sodium salt hydrate, nalidixic acidl	antibiotic	14986	6253	19.0	19.0
sodium lauryl sulfate, sodium lauryl sulfate (sodium dodecyl sulfate), sodium lauryl sulfate (sds)	antiseptic	13395	5139	18.9	18.9
promethazine, promethazine hydrochloride	antihistaminic	15006	6273	18.8	18.8
pramoxine, pramoxine hydrochloride	anesthetic	15007	6274	18.8	18.8
bromperidol	antipsychotic	14696	6843	18.7	18.7
terguride, terguride hydrogen maleate's(-)-, terfenadine	antiparkinsonian	13755	4573	18.6	18.6

aminobenzoate potassium, potassium p-aminobenzoate 0.32gm, paminobenzoate, potassium p-aminobenzoate, p-aminobenzoic acid potassium salt	dermatologic, phamaceutic aid	14027	6486	18.5	18.5
hydroxyprogesterone caproate, hydroxyprogesterone caproate (17-alpha-hydroxyprogesterone caproate)	progestogen	13640	5388	18.3	18.3
magnesium phosphate hydrate	vitamin	14043	6502	18.3	18.3
stanozolol	steroid	13521	3793	18.3	18.3
bromophenol blue	antiseptic	14758	6905	18.0	18.0
vitamin k5 1mg/ml, vitamin k5, vitamin k5 hydrochloride	vitamin	14071	6530	18.0	18.0
tiopronin, dextiopronin, tiopronin (n-2-mercaptopropionyl glycine)	antidote	13358	5894	17.7	17.7
lomefloxacin, lomefloxacin hydrochloride	antibiotic	13480	5250	17.6	17.6
neomycin, neomycin sulfate	antibiotic	13484	5254	17.5	17.5
sulfathiazole	antibiotic	13487	5257	17.5	17.5
minocycline, minocycline hydrochloride	antibiotic	13496	5266	17.3	17.3
bendroflumethiazide	diuretic	13513	5511	17.3	17.3
formylrifamycin, 3-formyl rifamycin	antibacterial (tuberculostatic)	13624	5981	17.2	17.2
pyridoxine, pyridoxine hydrochloride	vitamin	14136	6595	17.1	17.1
chamomile, apigenin, chamomile oil blue, chamomile oil, roman	therapeutic plant extract	13844	4662	17.1	17.1

phthalylsulfathiazole, phthalysulfathiazole	antibiotic	13516	5286	17.0	17.0
pantothenic acid, pantothenic acid calcium salt monohydrate, d-pantothenic acid calcium salt	vitamin	14147	6606	17.0	17.0
enoximone	cardiotonic	14841	6988	17.0	17.0
zirconium iv oxide, zirconium(iv) oxide	dermatologic	14150	6609	17.0	17.0
methyl nicotinate	dermatologic	13587	3859	16.9	16.9
moxifloxacin, moxifloxacin hydrochloride, moxifloxacin hcl	antibiotic	13527	5297	16.9	16.9
diosmin	capillary protectant	16018	7944	16.8	16.8
ipecac syrup		13880	4326	16.7	16.7
gluconolactone	antidote	16028	7954	16.7	16.7
cobalamin, cobalamin concentrate, cobalamine concentrate	vitamin	14173	6632	16.7	16.7
sertraline, sertraline hydrochloride	antidepressant	14340	4513	16.7	16.7
bentonite	phamaceutic aid	14877	7024	16.6	16.6
glucose, agar, galactose, l-galactose, d-(+)-mannose, glucose (d)	diagnostic aid, nutrient- fluid and nutrient replenisher, laxative	13445	5981	16.5	16.5
fluoxetine, fluoxetine hydrochloride	antidepressant	14347	4520	16.5	16.5
butoxamine, butoxamine hydrochloride	antidiabetic	14880	7027	16.5	16.5
artemisinin, artemesinin, artemisin	antimalarial	13673	6030	16.5	16.5
quinine hydrobromide	antimalarial	13158	5022	16.5	16.5
ketoprofen, dexketoprofen, s (+) -ketoprofen	analgesic, antiinflammatory	13608	3880	16.4	16.4

methimazole	thyroid	13775	5523	16.3	16.3
meclocycline, meclocycline sulfosalicylate, meclocycline sulfosalicylate salt	antibacterial	13566	5336	16.2	16.2
bithionoloxide	anthelminithic	14905	7052	16.2	16.2
zinc sulfate	antiseptic	13569	5313	16.2	16.2
sodium chromate cr 51, sodium chromate	diagnostic aid	13486	6022	16.0	16.0
hydroxyzine, hydroxyzine hydrochloride, hydroxyzine dihydrochloride, hydroxyzine (dihydrochloride)	anxiolytic	14380	4553	15.9	15.9
phosphatemonobasic, potassium phosphate monobasic, sodium phosphate monobasic	laxative	13489	6025	15.9	15.9
tubocurarine chloride, tubocurarine, tubocurarine chloride pentahydrate (+)	neuromuscular blocking agent	16105	8031	15.9	15.9
oxolinic acid	antibiotic	13591	5361	15.9	15.9
idebenone	nootropic., nootropic	14318	6242	15.8	15.8
glipizide	antidiabetic, antidepressant	13815	5563	15.7	15.7
carglumic acid, carbamylglutamic acid	nutrient	14951	7098	15.7	15.7
bioallethrin	insecticide	14955	7102	15.6	15.6
trioxsalen	dermatologic	16133	8059	15.6	15.6
butylated hydroxyanisole	phamaceutic aid	14958	7105	15.6	15.6
methylcellulose, methyl-cellulose	laxative	13940	4386	15.6	15.6
a-casein dephosphorylated		14269	6728	15.5	15.5
moxalactam, moxalactam disodium, moxalactam disodium salt	antibiotic	13618	5388	15.4	15.4
phenyltoloxamine	antihistaminic	13319	5558	15.4	15.4
flumequine	antibacterial	16157	8083	15.4	15.4

bifonazole	antifungal	13762	6119	15.3	15.3
bisoctrizole	dermatologic	14986	7133	15.3	15.3
psyllium husk (metamucil)		13542	6078	15.2	15.2
mezlocillin, mezlocillin sodium	antibiotic	13634	5404	15.2	15.2
zinc acetate	hemostatic	13663	5661	15.1	15.1
clorsulon	anthelminithic	13780	6137	15.0	15.0
ethotoin	anticonvulsant., anticonvulsant	14430	4603	15.0	15.0
sulindac, sulindac sulfide, sulindac sulfone	antineoplastic	16323	7855	14.9	14.9
enoxolone, 18alpha-glycyrrhetinic acid, enoxolone (18beta-glycyrrhetinic acid)	antiinflammatory	16201	8127	14.9	14.9
fleroxacin	antibiotic	13796	6153	14.8	14.8
vitamin e, alpha-tocopherol, alpha-tocopherol 0.003mg, alpha-tochopherol	vitamin	14324	6783	14.8	14.8
naltrexone, naltrexone hydrochloride dihydrate, naltrexone hydrochloride	antidote	16212	8138	14.8	14.8
cephalosporin cephalosporin c zinc salt	antibiotic	13801	6158	14.7	14.7
carbamide peroxide	antiseptic	15033	7180	14.7	14.7
aluminum phosphate	antacid	15043	7190	14.6	14.6
bipenamol, bipenamol hydrochloride	antidepressant	15043	7190	14.6	14.6
levofloxacin, ofloxacin, levofloxacin hcl	antibiotic	13678	5448	14.5	14.5
n desmethylclozapine, n-desmethylclozapine	antipsychotic	14418	6342	14.5	14.5
sucrose, inulin, sucralfate	diagnostic aid, antiulcerative, nutrient	13596	6132	14.4	14.4
docosanol	antiviral	13833	6190	14.3	14.3
carsalam	analgesic	15074	7221	14.2	14.2
pipemidic acid	antibiotic	13839	6196	14.2	14.2

quinine urea hydrochloride	antimalarial	13293	5157	14.2	14.2
calcium ascorbate, calcium l-ascorbate dihydrate	vitamin	14377	6836	14.1	14.1
quinine bisulfate	antimalarial	13299	5163	14.1	14.1
edetate sodium, edetatedta, ethylenediaminetetraacetic acid, calcium disodium salt hydratethylenediaminetetraacetic acidisodium salt dihydrate	antidote	13623	6159	14.0	14.0
ergosterol, ergosterol 1.25mg	vitamin	14389	6848	14.0	14.0
clemastine, clemastine fumarate	antihistaminic	13417	5656	13.9	13.9
pimagedine, pimagedine hydrochloride, pimagedine aminoguanidine bicarbonate (1-aminoguanidinium hydrogen carbonate), aminoguanidine bicarbonate (1-aminoguanidinium hydrogen carbonate)	antidiabetic	16068	7700	13.9	13.9
thioridazine, thioridazine hydrochloride	antipsychotic	14491	4664	13.9	13.9
edetate calcium disodium, edetate calcium disodium (calcium disodium versenate), sodium edetate (trisodium ethylenediamine tetracetate)	antidote	13638	6174	13.8	13.8
carvacrol 0.5%, carvacrol	anthelminithic	13315	5179	13.8	13.8
cinchonine sulfate, cinchonine	antimalarial	13315	5179	13.8	13.8

proflavine, proflavine dihydrochloride, proflavine hemisulfate salt hydrate, powder	antiseptic	13717	5461	13.8	13.8
rifamycin, rifamycin sv	antibiotic	13867	6224	13.8	13.8
pazufloxacin	antibiotic	13870	6227	13.8	13.8
oleic acid, oleic acid (cis-9-octadecenoic acid), oleic acid, potassium salt	diagnostic aid	13643	6179	13.8	13.8
amoxapine	antidepressant	14498	4671	13.7	13.7
clinafloxacin, clinafloxacin hydrochloride, clinafloxacin hcl	antibiotic	13874	6231	13.7	13.7
creosote		13322	5186	13.7	13.7
penicillin g, penicillin g potassium, penicillin g potassium salt, penicillin g potassium (benzyl penicillin potassium)	antibacterial	13729	5499	13.7	13.7
arsambide	antiamebic	15121	7268	13.7	13.7
polystyrene sulfonate, sodium polystyrene sulfonate, sodium polystyrene sulfonate suspension (15g/60ml)	antidote	13652	6188	13.6	13.6
progesterone caproate, progesterone caproate (17alpha-hydroxyprogesterone)	progestogen	13951	5699	13.6	13.6
eticloprideticlopride hydrochloride's(-)	gastroprokinetic	15126	7273	13.6	13.6
chloralose, chloralose (alpha-chloralose)	sedative	14488	6412	13.5	13.5
sodium phosphate, dibasic, phosphate dibasic, potassium phosphate dibasic, sodium phosphate dibasic	laxative	13666	6202	13.4	13.4
ferrous bromide	vitamin	14433	6892	13.4	13.4

clomipramine, clomipramine hydrochloride	antidepressant	14518	4691	13.4	13.4
aminothiazole, aminothiazole (2-aminothiazole)	thyroid	16115	7747	13.4	13.4
tosufloxacin	antibiotic	13902	6259	13.3	13.3
arginine, arginine hydrochloride, l-arginine, l-argininie hydrochloride	antidote	13678	6214	13.3	13.3
peruvian balsam		14446	6905	13.2	13.2
acetarsone, acetarsol	antiprotozoal	13353	5217	13.2	13.2
citrate monohydrate, citric acid, citric acid trisodium salt dihydrate, potassium citrate monohydrate	anticoagulant, misc- antiurolithic	13789	5787	13.2	13.2
succimer, succimer (meso-2,3-dimercaptosuccinic acid)	antidote	13687	6223	13.2	13.2
ethacridinethacridine lactatethacridine lactate (acrinol (6,9-diamino-2-ethoxyacrinide lactate monohydrate))	antiseptic	13915	6272	13.2	13.2
tartrazine	n/a	14065	4883	13.1	13.1
ethenzamidethenzamide (2-ethoxybenzamide)	analgesic	14523	6447	13.0	13.0
etidronic acid, etidronatetidronate disodium, etidronic acidisodium salt, etidronic acid (1-hydroxyethane-1,1-diphosphonic acid)	bone resorption inhibitor	13994	5742	13.0	13.0
niacinamide, nicotinamide niacinamide, niacinamide hydrochloride, niacinamide (nicotinamide)	dermatologic	14470	6929	12.9	12.9

vitamin d2, ergocalciferol, oleovitamin a and d, calciferol	vitamin	14472	6931	12.9	12.9
cianidanol, epicatechin, epicatechin-(-), catechin-(+,-) hydrate, cianidanol (catechin), epicatechin (-)-	antidiarrheal, n/a	16507	8039	12.9	12.9
ferrous lactate, formate, sodium formate	dermatologic	14473	6932	12.9	12.9
pyrimethamine	antimalarial	13373	5237	12.9	12.9
ipratropium, ipratropium bromide, ipratroprium bromide	bronchodilator	13489	5728	12.8	12.8
betahistine, betahistine hydrochloride	vasodilator	16405	8331	12.8	12.8
furosemide	diuretic	13818	5816	12.7	12.7
enprofylline	bronchodilator	15200	7347	12.7	12.7
magnesium acetate	tocolytic	13722	6258	12.7	12.7
doxycycline	antibiotic	13800	5570	12.6	12.6
indapamide	antihypertensive	13573	5276	12.5	12.5
androstenedione, androsterone sodium sulfate	steroid	15225	7372	12.4	12.4
aluminum clofibrate, clofibric acid, clofibric acid (2-(4-chlorophenoxy)-2-methylpropionic acid)	antihyperlipidemic	15226	7373	12.4	12.4
ribavirin	antiviral	13450	5260	12.4	12.4
fosfosal, fosfosal (o-carboxyphenyl phosphate)	analgesic	14575	6499	12.3	12.3
methylephedrine, methylphedrine, methylphedrine ((1r,2s)-(-)-n-methylephedrine)	nootropic	14575	6499	12.3	12.3
phthalylsulfacetamide	antibiotic	13815	5585	12.3	12.3

acriflavine, acriflavinium hydrochloride, acriflavine hydrochloride, acriflavine (acriflavinium hydrochloride)	antibacterial	13410	5274	12.3	12.3
colestipol, colestipol hydrochloride, tetraethylenepentamine	antihyperlipidemic	13852	5850	12.2	12.2
benmoxin	antidepressant	15242	7389	12.2	12.2
selenium sulfide	dermatologic, antifungal	14529	6988	12.2	12.2
betaine, betaine hydrochloride, betahistine hydrochloride	antidote	13758	6294	12.2	12.2
rifampin, rifampicin, rifampicin (rifampin)	antibacterial	13416	5280	12.2	12.2
bumetrizole	dermatologic	15253	7400	12.1	12.1
bronopol	antiseptic	15257	7404	12.0	12.0
penicillin g procaine, penicillin g (procaine salt)	antibiotic	13845	5615	11.9	11.9
iodine, iodine tincture unk, iodine resublimed, p.a., iodine tincture, iodine resublimed p.a., iodine 0.1 mo ici/i	antiseptic, thyroid	14073	5821	11.8	11.8
sulfamerazine	antibiotic	13851	5621	11.8	11.8
sulbactam, sulbactam sodium	antibiotic	13852	5622	11.8	11.8
bromthymol blue 0.21mg, bromthymol blue, bromthymol blue, sodium salt	antiseptic	13849	5593	11.7	11.7
domperidone, domperidone maleate	antiemetic	16506	8432	11.7	11.7
cyclacillin	antibiotic	13856	5626	11.7	11.7

nitrofurazone, nitrofural, nitrofurazone (5-nitro-2-furaldehyde semicarbazone)	antibiotic, antiseptic	13859	5629	11.6	11.6
kanamycin, kanamycin acid, kanamycin sulfate, kanamycin a sulfate	antibiotic	13863	5633	11.6	11.6
furaltadone, levofuraltadone, furaltadone hydrochloride	antibiotic, antibacterial	13865	5635	11.6	11.6
nithiamide, nithiamide (2-acetamido-5-nitrothiazole)	antiprotozoal	13455	5319	11.5	11.5
perchlorate monohydrate, perchloratemonohydrate, sodium perchlorate monohydrate	thyroid	14093	5841	11.5	11.5
brilliant green, brilliant green 0.1gm	antiseptic	13872	5616	11.4	11.4
zaprinast	antihistaminic, bronchodilator	16293	7925	11.4	11.4
sulfapyridine	antibiotic	13878	5648	11.3	11.3
calcium propionate 10%, calcium propionate	antifungal	13513	5323	11.3	11.3
karaya powder		14163	4609	11.3	11.3
alitretinoin, isotretinoin, neovitamin a, retinoic acid, tretinoin, 9-cis-retinoic acid, 13-cis-retinoic acid, isotretinon, tretinon, alitretinoin (9-cis-retinoic acid), tretinoin (retinoic acid, all trans), neovitamin a (13-cis-retinal)	dermatologic, antineoplastic, vitamin	14606	7065	11.2	11.2
cloxyquin, cloxyquin (5-chloro-8-hydroxy-quinoline)	antibacterial	14056	6413	11.2	11.2
minocycline, minocycline hydrochloride	antibiotic	13888	5658	11.2	11.2
human insulin isophane nph	antidiabetic	14115	5863	11.2	11.2

apramycin	antibiotic	16559	8485	11.1	11.1
chloroquine phosphate, chloroquine, chloroquine diphosphate, chloroquine diphosphate salt	antimalarial	13479	5343	11.1	11.1
acyclovir, acyclovir (acycloguanosine)	antiviral	13480	5344	11.1	11.1
deferoxamine, deferoxamine mesylate	antidote	13837	6373	11.1	11.1
chromic chloride, chromic chloride cr 51	phamaceutic aidiagnostic aid	14622	7081	11.0	11.0
sodium salicylate	analgesic	16574	8500	11.0	11.0
propantheline, propantheline bromide	antispasmodic	15607	6874	11.0	11.0
benztropine, benztropine mesylate, benztropine methane-sulfonate	antiparkinsonian	14649	4822	11.0	11.0
cloxacillin, cloxacillin sodium, cloxacillin sodium salt	antibiotic	15610	6877	10.9	10.9
erythromycin estolaterythromycin estolate (erythromycin propionate lauryl sulfate)	antibiotic	13905	5675	10.9	10.9
amodiaquine, amodiaquine hydrochloride, amodiaquin dihydrochloride dihydrate, amodiaquine dihydrochloride, amodiaquin	antimalarial	13491	5355	10.9	10.9
metronidazole	antiprotozoal	13493	5357	10.9	10.9
sulfamethizole	antibiotic	15619	6886	10.8	10.8
polymyxin b, polymyxin b sulfate	antibiotic	13913	5683	10.8	10.8
sucralfate		13857	6393	10.8	10.8
alovudine	antiviral	15364	7511	10.8	10.8

miltefosine, miltefosine (hexadecyl phosphocholine)	antineoplastic	16713	8245	10.7	10.7
vitamin b4, vitamin b4 (adenine)	vitamin	14653	7112	10.6	10.6
ascorbic acid	vitamin	14655	7114	10.6	10.6
sulfameter	antibiotic	13934	5704	10.5	10.5
phenoxyacetic acid 40%, phenylacetate, phenoxyacetic acid	dermatologic	13973	5971	10.4	10.4
apomorphine, apomorphine hydrochloride, r(-) apomorphine hydrochloride hemihydrate	antiparkinsonian	16629	8555	10.4	10.4
norepinephrine, norepinephrine bitartrate, arterenol (norepinephrine)	antihypotensive	13704	5407	10.4	10.4
riboflavin, riboflavine	vitamin	14683	7142	10.3	10.3
meglumine, meglumine (n-methyl-d-glucamine)	antiprotozoal	13533	5397	10.2	10.2
benzbromarone	misc- uricosuric, uricosuric	16758	8290	10.2	10.2
moricizine, moricizine hydrochloride	antiarrhythmic	13988	5986	10.2	10.2
rolitetracycline	antibiotic	13953	5723	10.2	10.2
sodium sulfate, sulfate, potassium sulfate, fine crystal	laxative	13902	6438	10.2	10.2
dexecadotril, ecadotril, racecadotril	antihypotensive, antidiarrheal	16762	8294	10.2	10.2
thyroxine i 125, dextrothyroxine, l-thyroxine, thyroxine, dextrothyroxine sodium, thyroxine (l), l-thyroxine [(3-[4-(4-hydroxy-3,5-diiodophenoxy)-3,5-diiodophenyl]-l-alanine] = t4	antihyperlipidemic, thyroid	14181	5929	10.2	10.2

pridinol, pridinol methanesulfonate salt, pridinol methanesulfonate	antiparkinsonian	16664	8590	10.0	10.0
secnidazole	antiamebic	16773	8305	10.0	10.0
casanthranol		14230	4676	10.0	10.0
clomiphenenclomiphene, zuclomiphene, clomiphene citrate, clomiphene citrate (z,e)	estrogen, steroid, pituitary	16672	8598	10.0	10.0
calcium phosphate tribasic	vitamin	14709	7168	9.9	9.9
anthranilic acid, anthranilic acid (2-aminobenzoic acid)	antibacterial	13550	5414	9.9	9.9
kanamycin, kanamycin acid, kanamycin sulfate, kanamycin a sulfate	antibiotic	13971	5741	9.9	9.9
sorbitol, liniment 60%, mannitol, liniment, d-mannitol, d-sorbitol, dulcitol	diuretic, phamaceutic aidermatologic, diagnostic aid	14011	6009	9.8	9.8
clofazimine	antibacterial	13560	5424	9.8	9.8
zinc sulfide	vitamin	14724	7183	9.8	9.8
sulfaguanidine	antibiotic	13983	5753	9.7	9.7
benzthiazide	diuretic	14024	6022	9.7	9.7
sodium thiosulfate, thiosulfate	antidote	13938	6474	9.7	9.7
medroxyprogesterone, 6_-methyl-17_-hydroxy-progesterone (medroxyprogesterone)	progestogen	14216	5964	9.6	9.6

ephedrine, pseudoephedrinephedrinen methyl, pseudoephedrine hydrochloridephedrine (1r,2s) hydrochloridephedrine hydrochloride, (1r,2s)-(-)-ephedrine, pseudoephedrine hcl (+), pseudoephedrine, (1s,2s)-(+)-, pseudoephedrine, (1r,2r)-(-)-, n-methyl(-)ephedrine	decongestant, bronchodilator	13699	5938	9.6	9.6
naphthol	anthelminthic	13571	5435	9.6	9.6
glimepiride	antidiabetic	14219	5967	9.6	9.6
pidolic acid, pyroglutamic acidl (dl-2-pyrrolidone-5-carboxylic acid), pidolic acid, pyroglutamic acidl (dl-2-pyrrolidone-5-carboxylic acid)	antiseptic, unclassified	14177	6534	9.5	9.5
coumarin	anticoagulant	14033	6031	9.5	9.5
nonivamide, desmethyldihydrocapsaicin, nonivamide (pelargonic acid vanillylamide), nonivamide (n-vanillylnonanamide)	nootropic	14786	6710	9.5	9.5
gatifloxacin	antibiotic	13999	5769	9.4	9.4
amiloride, amiloride hydrochloride, amiloride hydrochloride dihydrate	diuretic	16725	8651	9.4	9.4
guanadrel, guanadrel sulfate, guanethidine sulfate	antihypertensive	13765	5468	9.4	9.4
isosorbide	antianginal, diuretic	14044	6042	9.4	9.4
methyclothiazide	diuretic	14044	6042	9.4	9.4

nedocromil, nedocromil sodium	antihistaminic	13717	5956	9.3	9.3
atovaquone	antimalarial	13590	5454	9.3	9.3
miglitol	antidiabetic	14241	5989	9.2	9.2
albendazole	anthelminithic	13592	5456	9.2	9.2
nafcillin, nafcillin sodium, nafcillin sodium salt monohydrate	antibiotic	14021	5791	9.1	9.1
strophanthin k, strophanthin k 1/120gr, strophanthidin	cardiotonic	14062	6060	9.1	9.1
permanganate, potassium permanganate	antibacterial	13601	5465	9.1	9.1
zafirlukast	antiasthmatic., bronchodilator	13734	5973	9.1	9.1
vitamin b12 with intrinsic factor concentrate 6 ugm, vitamin b12, vitamin b12 (cyanocobalamin)	vitamin	14788	7247	9.0	9.0
norfloxacin	antibiotic	14033	5803	8.9	8.9
spironolactone	diuretic	16773	8699	8.9	8.9
cefpodoxime proxetil	antibiotic	14037	5807	8.9	8.9
pyridoxamine, pyridoxamine phosphate, pyridoxamine, dihydrochloride	vitamin	14306	5124	8.8	8.8
fenofibrate	antihyperlipidemic	14079	6077	8.8	8.8
chlortetracycline, chlortetracycline hydrochloride	antibiotic	15774	7041	8.8	8.8
spiroxatrine	vasodilator	14310	5128	8.8	8.8
cycloserine, levcycloserine, l-cycloserine, d-cycloserine, cycloserine'(s)-(-)-	antibacterial	13620	5484	8.8	8.8

perfluoropolymethylisopropyl ether, rauwolfia serpentina, safflower oil, soybean oil, veratrum viride, perfluoropolymethylisopropyl ether 50%, veratrum viride eq 75mgwholero	antihypertensive, antihyperlipidemic, diagnostic aid, therapeutic plant extract	14087	6085	8.7	8.7
sennosides, sennoside a, senna, sennoside b, senna syrup (sennosides 8.8mg/5ml)	laxative	14006	6542	8.7	8.7
sulfabenzamide	antibiotic, antibacterial	14050	5820	8.6	8.6
rubidium iodide	nutrient- iodine source	14012	6548	8.6	8.6
isoxicam	antiinflammatory	16807	8733	8.5	8.5
antimony trisulfide colloid	phamaceutic aid	15553	7700	8.5	8.5
cirazoline	antidepressant	15553	7700	8.5	8.5
nitrendipine	antihypertensive	16816	8742	8.5	8.5
ticlopidine, ticlopidine hydrochloride	antithrombotic	14105	6103	8.4	8.4
sodium iodide	nutrient	14028	6564	8.4	8.4
glycocyamine 100mglycocyamine	cardiotonic	14109	6107	8.4	8.4
epiestriol, estriol	estrogen, steroid	15568	7715	8.3	8.3
antazoline, atazoline, antazoline hydrochloride	antihistaminic, antihypertensive	13781	6020	8.3	8.3
chlormadinone acetate	progestogen	14301	6049	8.3	8.3
oxacillin, oxacillin sodium	antibiotic	14072	5842	8.3	8.3
ethinyl estradiol, ethinyl estradiol (17_-ethynylestradiol)	estrogen	14306	6054	8.3	8.3
harmine, harmine hydrochloride	n/a	14341	5159	8.2	8.2
levofloxacin, ofloxacin, levofloxacin hcl	antibiotic	14079	5849	8.2	8.2

bambuterol, bambuterol hydrochloride	bronchodilator	15593	7740	8.1	8.1
stannous fluoride	antiseptic	14866	7325	8.0	8.0
azathioprine	immunosuppressa nt	16878	8804	7.8	7.8
antazoline phosphate salt, antazoline phosphate	antihistaminic	13818	6057	7.8	7.8
amiodarone, amiodarone hydrochloride	antiarrhythmic	14150	6148	7.8	7.8
sarafloxacin, sarafloxacin hydrochloride, sarafloxacin hcl	antibiotic	14308	6665	7.7	7.7
adenosine	antiarrhythmic	14154	6152	7.7	7.7
mecamylamine, mecamylamine hydrochloride	antihypertensive	13867	5570	7.7	7.7
sodium nitroprusside, nitroprusside	antihypertensive	13868	5571	7.7	7.7
tamoxifen, tamoxifen citrate	antineoplastic	14300	5046	7.7	7.7
nifenazone	antiinflammatory	16894	8820	7.6	7.6
lithium benzoate	antipsychotic	14831	5004	7.6	7.6
berberine, berberine chloride, berberine bisulfate	antiprotozoal	13694	5558	7.5	7.5
dantrolene, dantrolene sodium, dantrolene sodium salt	muscle relaxant (skeletal)	16906	8832	7.5	7.5
azatadine, azatadine maleate	antihistaminic	13837	6076	7.5	7.5
terconazole	antifungal	13744	5554	7.5	7.5
clopidol	antibacterial	14326	6683	7.5	7.5
saccharin	pharmaceutic aid, phamaceutic aid	16912	8838	7.4	7.4
nialamide	antidepressant	14839	5012	7.4	7.4
rose bengal, rose bengal sodium i 125	diagnostic aid	14101	6637	7.4	7.4

ammonium citrate, ammonium citrate (ammonium hydrogencitrate), ammonium citrate tribasic	antiseptic	14128	5872	7.3	7.3
pravastatin, pravastatin sodium	antihyperlipidemic	14181	6179	7.3	7.3
norepinephrine, norepinephrine bitartrate, arterenol (norepinephrine)	antihypotensive	13890	5593	7.3	7.3
gentamicin sulfate, micronomicin, gentamicine sulfate	antibiotic	15895	7162	7.3	7.3
chlorzoxazone	muscle relaxant (skeletal)	16934	8860	7.2	7.2
estradiol benzoate, _-estradiol 3-benzoate	estrogen	14375	6123	7.2	7.2
estrogens, esterified, estradiol, epiestradiol, 17alpha-estradiol, estrogens, estradiol-17 beta, _-estradiol	estrogen	14378	6126	7.2	7.2
ritodrine, ritodrine hydrochloride	tocolytic	14117	6653	7.2	7.2
warfarin, warfarin potassium, warfarin (3-3-alpha-acetonylbenzyl-4-hydroxycoumarin)	anticoagulant	14192	6190	7.1	7.1
triacetin	antifungal	13770	5580	7.0	7.0
crospovidone, povidone iodine, povidone, povidone-iodine, povidone iodine (polyvinylpyrrolidone-iodine complex)	antibacterial, phamaceutic aid, antiseptic	13727	5591	7.0	7.0
vitamin d2, ergocalciferol, oleovitamin a and d, calciferol	vitamin	14950	7409	6.9	6.9
diloxanide furoate	antiamebic	17063	8595	6.9	6.9

penicillin g, penicillin g potassium, penicillin g potassium salt, penicillin g potassium (benzyl penicillin potassium)	antibacterial	14169	5939	6.8	6.8
quinethazone	diuretic	14217	6215	6.8	6.8
tolbutamide	antidiabetic	14406	6154	6.7	6.7
bendazol	vasodilator	15705	7852	6.7	6.7
magnesium, magnesium chloride, magnesium chloride 6-hydrate	laxative	14401	4847	6.7	6.7
riboflavin 5'-phosphate, riboflavin phosphate, riboflavin phosphate sodium (flavin monomucleotide)	vitamin, vitamin (enzyme cofactor)	14968	7427	6.7	6.7
chlorpheniramine polistirex, chlorpheniramine, chlorpheniramine maleate, chlorpheniramine gluconate 3mg/ml, chlorpheniramine (s) maleate	antihistaminic	13891	6130	6.7	6.7
insulin injectionusp purified pork, regular iletin ii (insulin injection, usp purified pork)	antidiabetic	14415	6163	6.6	6.6
amylene hydrate		15001	6925	6.6	6.6
dihydroxysuccinic acid 0.740gr, tartaric acid, tartaric acid l-(+)	antibacterial, unclassified	13752	5616	6.6	6.6
benzonatate	antitussive	13898	6137	6.6	6.6
nifuroxime	antibacterial (topical), antibiotic	13754	5618	6.5	6.5
flubendazole	anthelminithic	14394	6751	6.5	6.5
cypermethrin, alpha cypermethrin, alpha-cypermethrin	insecticidectopara siticide	15723	7870	6.5	6.5

methenamine hippurate, methenamine mandelate, methenamine mandelate (hexamethylenetetramine mandelate)	antibacterial	13756	5620	6.5	6.5
meclocycline, meclocycline sulfosalicylate, meclocycline sulfosalicylate salt	antibacterial	14191	5961	6.4	6.4
nordefrin, nordefrin hydrochloride, nordefrin hydrochloride (+/-)	antihypotensive	17014	8940	6.4	6.4
sulpiride, levosulpiride, sulpiride (+/-)-(levosulpiride)	antipsychotic, antidepressant	15020	6944	6.3	6.3
mitotane, mitotane (1-2-chlorophenyl-1-4-chlorophenyl-2,2-dichloroethane)	antineoplastic	14372	5118	6.3	6.3
pyrogallol, pyrogallin	neuromuscular blocking agent	15022	6946	6.3	6.3
carnitine, levocarnitine, levocarnitine propionate hydrochloride, dl-carnitine hydrochloride (levocarnitine), carnitine chloride	antihyperlipidemic	14250	6248	6.3	6.3
lorglumide, lorglumide sodium salt	diagnostic aid	14453	5271	6.2	6.2
butoconazole, butoconazole nitrate	antifungal	13819	5629	6.2	6.2
oxiracetam	nootropic	15031	6955	6.2	6.2
levodopa, levodopa (l-beta-3,4-dihydroxyphenylananine)	antiparkinsonian	14907	5080	6.2	6.2

tranexamic acid, tranylcypromine hydrochloride, tranylcypromine sulfate	hemostatic	14255	6253	6.2	6.2
anagrelide, anagrelide hydrochloride	antithrombotic	14256	6254	6.2	6.2
glutamine, l-glutamine	nutrient	16767	8399	6.1	6.1
amithiozone, thiacetazone, thiacetazone (amithiozone)	antibacterial	14429	6786	6.0	6.0
clemizole, clemizole hydrochloride	antihistaminic	13932	6171	6.0	6.0
estronestriol	estrogen	14454	6202	6.0	6.0
phenothiazine	anthelminithic	14433	6790	6.0	6.0
sulfanilate zinc, sulfanilate zinc (sulfanilic acid)	antibiotic	14434	6791	6.0	6.0
inosine	nutrient	17056	8982	5.9	5.9
carbazochrome, carbazochrome (adrenochrome semicarbazone)	hemostatic	14272	6270	5.9	5.9
octodrine, octodrine (1,5 dimethyhexylamine)	decongestant	13939	6178	5.9	5.9
daptomycin	antibiotic	14224	5994	5.9	5.9
ciprofloxacin, ciprofloxacin hydrochloride	antibiotic	15999	7266	5.9	5.9
cefmenoxime, cefmenoxime hydrochloride	antibiotic	13844	5654	5.8	5.8
myristic acid	phamaceutic aid	14482	5300	5.7	5.7
pidolic acid, pyroglutamic acidl (dl-2-pyrrolidone-5-carboxylic acid), pidolic acid, pyroglutamic acidl (dl-2-pyrrolidone-5-carboxylic acid)	antiseptic, unclassified	14453	6810	5.7	5.7
nisoldipine	antihypertensive	13986	5689	5.7	5.7
oxamniquine	anthelminithic	13805	5669	5.7	5.7
voriconazole	antifungal	13851	5661	5.7	5.7

chloramine-t, chloramine-t hydrate	antibacterial	13809	5673	5.6	5.6
hexylresorcinol, 4 hexylresorcinol	anthelminithic	13813	5677	5.6	5.6
dextran 70 0.1%, hypromellose 2910 0.3%		13996	5699	5.5	5.5
enrofloxacin	antibiotic	14467	6824	5.5	5.5
florfenicol	antibiotic	14470	6827	5.5	5.5
diethylstilbestrol, diethylstilbesterol	antineoplastic	14420	5166	5.5	5.5
oleic acid, oleic acid (cis-9-octadecenoic acid), oleic acid, potassium salt	diagnostic aid	14240	6776	5.4	5.4
xylometazoline, xylometazoline hydrochloride	decongestant	13974	6213	5.4	5.4
strophanthin k, strophanthin k 1/120gr, strophanthidin	cardiotonic	14308	6306	5.4	5.4
fluconazole	antifungal	13872	5682	5.3	5.3
prasterone, dehydroepiandrosterone, prasterone (dehydroisoandrosterone), prasterone (dhea, dehydroepiandrosterone)	steroid	14504	5322	5.3	5.3
penicillin v, penicillin v (phenoxymethyl penicillinic acid)	antibiotic	14262	6032	5.3	5.3
linezolid	antibiotic	14263	6033	5.3	5.3
bretylium, bretylium tosylate	antiarrhythmic	14314	6312	5.3	5.3
sulfadiazine	antibiotic	14265	6035	5.3	5.3
gemfibrozil	antihyperlipidemic	14321	6319	5.2	5.2
dichlorodiphenylmethane 0.5fl oz/gal, dichlorodiphenylmethane, dichlorodiphenyl-methane	antiseptic	14265	6009	5.2	5.2
penicillinase		14260	6796	5.2	5.2

metrifudil	antiseizure	14515	5333	5.1	5.1
irbesartan	antihypertensive	14022	5725	5.1	5.1
carzenide, carzenide (4-carboxybenzenesulfonamide, 97%)	unclassified	14500	6857	5.1	5.1
methenamine, methenamine (hexamethylenetetramine)	antibacterial	13845	5709	5.0	5.0
gold sodium thiomalate, sodium aurothiomalate	antirheumatic	17237	8769	5.0	5.0
carbadox	antibacterial	14506	6863	5.0	5.0
protionamide, prothionamide (protionamide)	antibacterial	14507	6864	5.0	5.0
prochlorperazine, prochlorperazine dimaleate, prochlorperazine dimaleate salt	antiemetic	14493	4939	5.0	5.0
metformin, metformin hydrochloride, metformin (1,1-dimethylbiguanide hydrochloride)	antidiabetic	14530	6278	4.9	4.9
mephentermine, mephentermine sulfate, mephentermine hemisulfate	antihypotensive	14038	5741	4.8	4.8
thymol	antibacterial	13856	5720	4.8	4.8
idazoxan, idazoxan hydrochloride	antiparkinsonian	15133	7057	4.8	4.8
chlorhexidine gluconate, chlorhexidine gluconate mouthrinse	antiseptic	14290	6034	4.8	4.8
procainamide, procainamide hydrochloride, procainamide hcl	antiarrhythmic	14353	6351	4.7	4.7
triamterene, tretinoin	diuretic	14355	6353	4.7	4.7
strontium bromide hexahydrate	anticonvulsant	14991	5164	4.6	4.6

ephedrine, pseudoephedrinephedrinen methyl, pseudoephedrine hydrochloridephedrine (1r,2s) hydrochloridephedrine hydrochloride, (1r,2s)-(-)-ephedrine, pseudoephedrine hcl (+), pseudoephedrine, (1s,2s)-(+)-, pseudoephedrine, (1r,2r)-(-)-, n-methyl(-)ephedrine	decongestant, bronchodilator	14027	6266	4.6	4.6
sulfanilamide, aminobenzenesulfonamide'2-	antibiotic	16104	7371	4.5	4.5
betazole, betazole hydrochloride	diagnostic aid	14306	6842	4.5	4.5
suloctidil	vasodilator	17193	9119	4.5	4.5
erythromycin stearate	antibiotic	14315	6085	4.5	4.5
acetohexamide	antidiabetic	14562	6310	4.4	4.4
benzoic acid, (benzylsulfonamido)benzoic acid, p- 20%, benzoic-acid, benzoic acid sodium salt	diagnostic aid, phamaceutic aid	14317	6853	4.4	4.4
bicarbonate, bicarbonate granular, potassium bicarbonate, granular	nutrient-potassium supplement	14317	6853	4.4	4.4
allopurinol, allopurinol sodium	misc- antiurolithic, antiurolithic	17208	9134	4.3	4.3
hesperetin, hesperetin (vitamin p)	vitamin	17208	9134	4.3	4.3

sodium dodecylbenzenesulfonateng, dodecylbenzenesulfonic acid, sodium salt, tech, dodecylbenzenesulfonateng, dodecylbenzenesulfonic acid sodium salt tech	antiseptic	14320	6064	4.3	4.3
fosfomycin, phosphomycin, fosfomycin calcium (phosphomycin calcium salt)	antibiotic	14326	6096	4.3	4.3
epinephrine, (-)-epinephrine, racepinephrine	bronchodilator	14045	6284	4.3	4.3
alprenolol, alprenolol hydrochloride	antihypertensive	17214	9140	4.3	4.3
noscapine, noscapine hydrochloride	antitussive	16124	7391	4.3	4.3
vinorelbine, vinorelbine tartrate	antineoplastic	14487	5233	4.2	4.2
nitrophenol, p- 4.2%, nitrophenol, 4-nitrophenol	antiseptic	14326	6070	4.2	4.2
fipexide, fipexide hydrochloride	nootropic	17222	9148	4.2	4.2
loracarbef	antibiotic	14335	6105	4.2	4.2
malachite green	antiseptic	14329	6073	4.2	4.2
propylthiouracil, propylthiouracil (6-n-propyl-2-thiouracil)	thyroid	14578	6326	4.1	4.1
brompheniramine, brompheniramine maleate, brompheniramine (+/-)-	antihistaminic	14061	6300	4.1	4.1
cefsulodin sodium, cefsulodin sodium salt, cefsulodin	antibiotic	14572	6929	4.1	4.1
lamivudine	antiviral	13949	5759	4.1	4.1
griseofulvin	antifungal	13951	5761	4.0	4.0
enoxacin	antibiotic	14346	6116	4.0	4.0

amlodipine, amlodipine besylate	antianginal	14090	5793	4.0	4.0
betamipron, betamipron (n-benzoyl-b-alanine)	antibacterial	14578	6935	4.0	4.0
cephalosporin cephalosporin c zinc salt	antibiotic	14578	6935	4.0	4.0
cetrizine hydrochloride 5mg, cetirizine, cetrizine, levocetirizine, cetirizine hydrochloride, cetirizine dihydrochloride	antihistaminic	15465	7496	4.0	4.0
letrozole	antineoplastic	14503	5249	3.9	3.9
azulene, azulene (sodium gualenate)	antacid	16958	8590	3.9	3.9
econazoleconazole nitrate	antifungal	13959	5769	3.9	3.9
flucytosine, flucytosine (5-fluorocytosine)	antifungal	13963	5773	3.8	3.8
piperacillin tazobactam, piperacillin-tazobactam	antibiotic	14363	6133	3.7	3.7
bumetanide	diuretic	14419	6417	3.7	3.7
racemethionine, methionine, l-methionine	hepatoprotectant, hepatic protectant, nutrient	17357	8889	3.7	3.7
xylitol, adonitol (ribitol)	pharmaceutic aid, phamaceutic aid	14595	5413	3.7	3.7
trifluridine	antiviral	13970	5780	3.7	3.7
acrivastine	antihistaminic., antihistaminic	14090	6329	3.6	3.6
nitrate, potassium nitrate	diuretic	14426	6424	3.6	3.6
leucine, d-leucine	nutrient	15492	7523	3.6	3.6
quinidine, quinine, quinidine sulfate, quinine sulfate, quinidine hydrochloride monohydrate, quinidine sulfate dihydrate	antimalarial	13930	5794	3.6	3.6

doxycycline hyclate, doxycycline hydrochloride	antibiotic	14374	6144	3.6	3.6
bephenium hydroxynaphthoate, bephenium hydroxy-naphthoate	anthelminithic	13936	5800	3.5	3.5
sulfadoxine	antibiotic	14380	6150	3.5	3.5
sulconazole, sulconazole nitrate	antifungal	13986	5796	3.4	3.4
iopanoic acid, iopanic acid	diagnostic aid	14384	6920	3.4	3.4
carbinoxamine, rotoxamine, carbinoxamine maleate, carbinoxamine maleate salt	antihistaminic	14110	6349	3.3	3.3
salicylanilide	antifungal	13993	5803	3.3	3.3
penicillin g benzathine	antibiotic	14390	6160	3.3	3.3
toluene, toluene diisocyanate, toluene diisocyanate (tolylene 2,4-diisocyanate, tech)	antiseptic	14386	6130	3.3	3.3
sulfasalazine	antiinflammatory	14222	4494	3.2	3.2
plasma protein fraction human		14459	6457	3.1	3.1
piperazine	anthelminithic	13961	5825	3.1	3.1
pregnenolone 50mg/ml, pregnenolone, prednisolone, pregnenolone sulfate, sodium salt	progestogen	14630	5448	3.1	3.1
clindamycin, clindamycin hydrochloride	antibiotic	15536	7567	3.1	3.1
terbutaline, terbutaline sulfate, terbutaline hemisulfate	bronchodilator	16230	7497	2.9	2.9
imipramine, prazepine, imipramine hydrochloride	antidepressant, anticonvulsant	15085	5258	2.9	2.9

guaiacolsulfonate, potassium guaiacolsulfonate, guaiacolsulfonic acid potassium salt	expectorant	14139	6378	2.9	2.9
sodium hydroxide	dermatologic	15273	7732	2.9	2.9
phenacemide, phenacemide (phenylacetylurea)	anticonvulsant	15088	5261	2.8	2.8
irsogladine, irsogladine maleate	antiulcerative	17056	8688	2.8	2.8
bismuth tribromophenate 4%, bismuth tribromophenate	antibacterial	13979	5843	2.8	2.8
cianidanol, epicatechin, epicatechin-(-), catechin-(+,-) hydrate, cianidanol (catechin), epicatechin (-)-	antidiarrheal, n/a	14650	5468	2.7	2.7
tomoxetine, atomoxetine, atomoxetine hydrochloride	nootropic, antidepressant	14156	4383	2.7	2.7
barium sulfate	diagnostic aid (radiopaque medium), diagnostic aid	14437	6973	2.7	2.7
phensuximide	anticonvulsant	15097	5270	2.7	2.7
sulfosalicylic acid	antiinflammatory	14653	5471	2.7	2.7
debrisoquin, debrisoquin sulfate	antihypertensive	14297	6666	2.7	2.7
norgestrel, norgestrel-(-)-d, norgestrel d(-) (levonorgestrel)	progestogen	14676	6424	2.6	2.6
miconazole, miconazole nitrate	antifungal	14034	5844	2.6	2.6
urethane, polyurethane, polyurethane foam	phamaceutic aid, antineoplastic	16252	7519	2.6	2.6
ramipril	antihypertensive	14172	5875	2.6	2.6

pyrilamine maleate, pyrilamine, pyrilamine maleate (mepyramine maleate)	antihistaminic	14157	6396	2.6	2.6
nicotinyl alcohol, nicotinyl alcohol tartrate, nicotinyl alcohol (3-pyridylcarbinol)	vasodilator	14175	5878	2.6	2.6
oxtriphylline	bronchodilator	14161	6400	2.5	2.5
thiamphenicol-glycinate, thiamphenicol, thiamphenicol glycinate hcl	antibiotic	14683	7040	2.5	2.5
lobeline, lobeline alpha (-) hydrochoride, lobeline (-) hydrochloride, lobeline sulfate	misc- respiratory stimulant, respiratory stimulant	17472	9004	2.5	2.5
ketotifen, ketotifen fumarate	antihistaminic	14167	6406	2.5	2.5
methylergonovine, propisergide, methylergonovine maleate	oxytocic, antimigraine	17389	9315	2.5	2.5
zanamivir		14688	7045	2.5	2.5
metampicillin, metampicillin sodium salt	antibiotic	14691	7048	2.4	2.4
glycerin, glucalox, glycerin (glycerol)	diagnostic aid, unclassified	14464	7000	2.3	2.3
albendazole oxide, ricobendazole, ricobendazole (albendazole oxide)	anthelminithic, anthelmintic (cestodes)	14704	7061	2.2	2.2
penicillin v potassium, penicillin v potassium (phenoxymethylpenicillinic acid potassium salt)	antibiotic	14461	6231	2.2	2.2

glucosamine, glucosamine hydrochloride, d-galactosamine hydrochloride, d-glucosamine 2-sulfate, d-glucosamine sulfate	antiarthritic, antirheumatic	17497	9029	2.2	2.2
mebhydrolin	antihistaminic	17114	8746	2.2	2.2
telenzepine, telenzepine dihydrochloride	antiulcerative	17121	8753	2.1	2.1
methoxamine, methoxamine hydrochloride	antihypotensive	14206	5909	2.1	2.1
spermidine, spermidine trihydrochloride	n/a	14688	5506	2.1	2.1
iproniazid, iproniazide phosphate, iproniazid (isonicotinic acid hydrazide), iproniazid phosphate salt	antidepressant	15132	5305	2.0	2.0
flufenamic acid	antiinflammatory	17512	9044	2.0	2.0
nonivamide, desmethyldihydrocapsaicin, nonivamide (pelargonic acid vanillylamide), nonivamide (n-vanillylnonanamide)	nootropic	15341	7265	2.0	2.0
fosfomycin tromethamine	antibacterial	14474	6244	2.0	2.0
kawain, kavain	n/a	14696	5514	1.9	1.9
isoproterenol, levisoprenaline, isoprenaline hydrochloride, isoproterenol hydrochloride, isoproterenol hydrochloride'(-)-, isoproterenol also 123	bronchodilator	14208	6447	1.8	1.8
phenylpropanol, phenylpropanol (1-phenyl 1-propanol)	choleretic	17146	8778	1.8	1.8

sodium propionate, propionate	phamaceutic aid	14083	5893	1.8	1.8
iodipamide	diagnostic aid	17452	9378	1.8	1.8
tolfenamic acid	antiinflammatory	17534	9066	1.8	1.8
rimantadine, flumadine, rimantadine hydrochloride, rimantadine (1-(1-adamantyl)ethylamine hydrochloride)	antiviral	14085	5895	1.8	1.8
carbomycin, carbomycin (magnamycin a)	antibiotic	14088	5898	1.7	1.7
sulfamethazine, sulfamethazine sodium salt	antibiotic	16323	7590	1.7	1.7
chloramphenicol	antibiotic	14492	6262	1.7	1.7
congo-red, congo red	diagnostic aid	14364	6733	1.7	1.7
dapsone, dds, dapsone (4-aminophenyl sulfone, 4,4'-diaminodiphenyl sulfone)	antibiotic, antibacterial	14496	6266	1.6	1.6
trichlormethiazide	diuretic	14561	6559	1.6	1.6
lidocaine n ethyl bromide, lidocaine n-ethyl bromide	anesthetic (local)	15372	7296	1.6	1.6
dapsone, dds, dapsone (4-aminophenyl sulfone, 4,4'-diaminodiphenyl sulfone)	antibiotic, antibacterial	14051	5915	1.6	1.6
levallorphan, levallorphan tartrate, levallorphan tartrate salt	antidote	14521	7057	1.5	1.5
ganciclovir, gancyclovir, ganciclovir sodium	antiviral	14102	5912	1.5	1.5
allylthioureallylthiourea (1-allyl-2-thiourea)	phamaceutic aid	14379	6748	1.5	1.5
cefuroxime, cefuroxime sodium salt, cefuroxime sodium	antibiotic	14510	6280	1.4	1.4
amyl nitrite, amyl nitrate inhalant	antihypertensive	14244	5947	1.4	1.4
acepromazine, acepromazine maleate	sedative	15386	7310	1.4	1.4
ronidazole	antiprotozoal	17491	9417	1.4	1.4
brucine, brucine sulfate	nootropic	17491	9417	1.4	1.4

anthracene 0.17%, anthracene	antineoplastic	14643	5389	1.4	1.4
ceftazidime, ceftazidime pentahydrate	antibiotic	14517	6287	1.3	1.3
albumin from human serum		14251	5954	1.3	1.3
xylose, l-xylose, d-xylose	diagnostic aid	14538	7074	1.3	1.3
silibinin, milk thistle, silymarin, silibinin (silybin)	hepatic protectant, therapeutic plant extract, misc-hepatoprotectant	17582	9114	1.3	1.3
valacyclovir, valacyclovir hydrochloride	antiviral	14117	5927	1.3	1.3
oxalic acid unk, oxalic acid, oxalic acid dyhydrate	hemostatic	14587	6585	1.2	1.2
sulfacetamide, sulfacetamide sodic hydrate	antibiotic	16363	7630	1.2	1.2
cyacetacide, cyacetacide (cyanoacetohydrazide)	antibacterial	14780	7137	1.2	1.2
aminosalicylic acid, 5 aminosalicylic acid, 4-aminosalicylic acid, p-aminosalicylic acid	antibacterial	14078	5942	1.1	1.1
methyldopate, methyldopate hydrochloride	antiseptic	14521	6265	1.1	1.1
tolmetin, tolmetin sodium, tolmetin sodium salt dihydrate	antiinflammatory	16368	7635	1.1	1.1
terazosin, terazosin hydrochloride	antihypertensive	14264	5967	1.1	1.1
ancitabine, ancitabine hydrochloride	antineoplastic	17522	9448	1.1	1.1
itopride, itopride hcl	gastroprokinetic	17602	9134	1.1	1.1
cefdinir	antibiotic	14129	5939	1.1	1.1
dyphylline, diprophylline, dyphylline (diprophylline (7-(2,3-dihydroxypropyl)-theophylline))	bronchodilator	14262	6501	1.0	1.0

spermine	n/a	14747	5565	1.0	1.0
sulfamoxole, sulfamoxole (n-(4,5-dimethyl-2-oxazolyl)-sulfanilamide)	antibiotic, antibacterial	14793	7150	1.0	1.0
ethoxyquin		15698	7729	1.0	1.0
clindamycin phosphate	antibiotic	14539	6309	1.0	1.0
hetastarchetastarch (hydroxyethyl starch)	plasma volume expander	14604	6602	1.0	1.0
hydroxyprogesterone, hydroxyprogesterone acetate 25mg, hydroxyprogesterone acetate (17alpha-acetoxyprogesterone)	progestogen	14788	6536	1.0	1.0
paraformaldehyde, formaldehyde solution, poly oxymethylene, formaldehyde	antiseptic	14534	6278	0.9	0.9
ephedrine, pseudoephedrinephedrine n methyl, pseudoephedrine hydrochloridephedrine (1r,2s) hydrochloridephedrine hydrochloride, (1r,2s)-(-)-ephedrine, pseudoephedrine hcl (+), pseudoephedrine, (1s,2s)-(+)-, pseudoephedrine, (1r,2r)-(-)-, n-methyl(-)ephedrine	decongestant, bronchodilator	14267	6506	0.9	0.9
aminosalicylic acid, 5 aminosalicylic acid, 4-aminosalicylic acid, p-aminosalicylic acid	antibacterial	14798	7155	0.9	0.9

arginine, arginine hydrochloride, l-arginine, l-argininie hydrochloride	antidote	14565	7101	0.9	0.9
tuaminoheptane, tuaminoheptane sulfate, tuaminoheptane (2-aminoheptane)	decongestant	14269	6508	0.9	0.9
stibogluconate, sodium stibogluconate	antiprotozoal	14753	5571	0.9	0.9
dopamine, dopamine hydrochloride, 3-hydroxytyramine (dopamine), dopamine (3-hydroxytyramine hydrochloride)	cardiotonic	14608	6606	0.9	0.9
cetyl alcohol, cetyl alcohol (1-hexadecanol)	antiseptic	14539	6283	0.9	0.9
guanabenz, guanabenz acetate	antihypertensive	14279	5982	0.8	0.8
thioguanine, thioguanosine, thioguanine (2-amino,6-mercaptopurine)	antineoplastic	14673	5419	0.8	0.8
cyproheptadine, cyproheptadine hydrochloride	antihistaminic	14276	6515	0.8	0.8
ephedrine, pseudoephedrinephedrine n methyl, pseudoephedrine hydrochloridephedrine (1r,2s) hydrochloridephedrine hydrochloride, (1r,2s)-(-)-ephedrine, pseudoephedrine hcl (+), pseudoephedrine, (1s,2s)-(+)-, pseudoephedrine, (1r,2r)-(-)-, n-methyl(-)ephedrine	decongestant, bronchodilator	14276	6515	0.8	0.8

flunarizine, flunarizine hydrochloride, flunarizine dihydrochloride	vasodilator	17550	9476	0.8	0.8
lidofenin, lidofenin (n-(2,6-dimethylphenyl-carbamoylmethyl)iminodiacetic acid)	diagnostic aid	14575	7111	0.8	0.8
hymecromone, methylumbelliferone, methyllumberriferone, hymecromone (4-methylumbelliferone free acid), 4-methyl lumberriferone	choleretic, n/a	17244	8876	0.7	0.7
docusate sodium, docusate sodium (dioctyl sulfosuccinate, sodium salt)	laxative	14713	5159	0.7	0.7
undecylenic acid, zinc undecylenate	antifungal, antifungal (topical)	14153	5963	0.7	0.7
amitriptyline, amitriptyline hydrochloride, amitryptiline hydrochloride	antidepressant	15207	5380	0.7	0.7
mafenide, mafenide hydrochloride, mafenide (p-aminomethylbenzenesulfonamide)	antibiotic	14560	6330	0.6	0.6
sulfaquinoxaline, sulfaquinoxaline sodium salt, sulfaquinoxaline sodium	antiprotozoal	17566	9492	0.6	0.6
arbutin 0.25gr, arbutin	dermatologic	14344	4616	0.6	0.6
althiazide	diuretic	14442	6811	0.5	0.5
clodronic acid, clodronate disodium, clodronate (dichloromethylenediphosphonic acid disodium salt)	bone resorption inhibitor	17650	9182	0.5	0.5

cysteamine, mercaptamine, cysteamine hydrochloride, mercaptamine hydrochloride (cysteamine), cysteamine (beta-mercaptoethylamine hydrochloride), cysteamine s-phosphate	antidote	14593	7129	0.5	0.5
stavudine, stavudine (2',3'-didehydro-3'-deoxythymidine)	antiviral	14165	5975	0.5	0.5
etidronic acid, etidronatetidronate disodium, etidronic acidisodium salt, etidronic acid (1-hydroxyethane-1,1-diphosphonic acid)	bone resorption inhibitor	14823	6571	0.4	0.4
colistin sulfate	antibiotic, antibacterial	14575	6345	0.4	0.4
iodine, iodine tincture unk, iodine resublimed, p.a., iodine tincture, iodine resublimed p.a., iodine 0.1 mo ici/i	antiseptic, thyroid	14569	6313	0.4	0.4
diphenhydramine, diphenhydramine hydrochloride	antihistaminic	14303	6542	0.4	0.4
aluminum lactate, aluminum lactate, 97%	antiseptic	14838	7195	0.4	0.4
mechlorethamine, mechlorethamine hydrochloride, mechlorethamine hcl	antineoplastic	14698	5444	0.4	0.4
beclamide, beclamide (n-benzyl 3 chloropropionamide)	anticonvulsant	15224	5397	0.3	0.3

arsenic, arsenic trioxide, arsenic trioxide (sodium meta-arsenite)	antineoplastic	14700	5446	0.3	0.3
cefonicid, cefonicid sodium	antibiotic	14173	5983	0.3	0.3
guaiacol cocodylate pnguaiacol	expectorant	14311	6550	0.3	0.3
clofibrate, clofibrate (2-2-methyl-propionic acid ethyl ester)	antihyperlipidemic	14654	6652	0.2	0.2
lithium citrate, tri lithium citrate, tri-lithium citrate	antipsychotic, antidepressant	15232	5405	0.2	0.2
paramethadione	anticonvulsant	15241	5414	0.0	0.0
zoxazolamine, zoxazolamine (2-amino-5-chlorobenzoxazole)	uricosuric	14631	7167	0.0	0.0
n,1-dimethylhexylamine 50mg/ml, n,1-dimethylhexylamine, n,n-dimethylhexylamine	decongestant	14330	6569	0.0	0.0
fomepizole, fomepizole hydrochloride, fomepizole (4-methylpyrazole)	antidote	14635	7171	-0.1	0.0
sodium phosphate, dibasic, phosphate dibasic, potassium phosphate dibasic, sodium phosphate dibasic	laxative	14755	5201	-0.1	0.0
undecylenic acid, zinc undecylenate	antifungal, antifungal (topical)	14203	6013	-0.2	0.0
cefprozil	antibiotic	14613	6383	-0.2	0.0
berberine, berberine chloride, berberine bisulfate	antiprotozoal	14159	6023	-0.2	0.0
fluvastatin, fluvastatin sodium, fluvastatin sodium salt	antihyperlipidemic	14681	6679	-0.2	0.0

hydroxyethyl cellulose (hyetellose)		14682	6680	-0.2	0.0
benzamil hydrochloride		15792	7823	-0.2	0.0
aminophylline	bronchodilator	14343	6582	-0.2	0.0
azaguanine-8		15796	7827	-0.3	0.0
procarbazine, procarbazine hydrochloride, procarbazine hcl	antineoplastic	14734	5480	-0.3	0.0
symclosene, symclosene (trichloroisocyanuric acid, 97%)	antibacterial	14887	7244	-0.3	0.0
tinidazole	antiprotozoal	14888	7245	-0.3	0.0
quinaldine blue, quinaldine blue (pinacyanol chloride)	antineoplastic	14737	5483	-0.3	0.0
pregnenolone acetate	progestogen	14874	6622	-0.4	0.0
molsidomine	antianginal	14504	6873	-0.4	0.0
benzoyl-peroxide, benzoyl peroxide	dermatologic	14390	4662	-0.4	0.0
allyl isothiocyanate, mustard oil, mustard oil, expressed unk"	dermatologic, therapeutic plant extract	14391	4663	-0.5	0.0
melatonin		15817	7848	-0.5	0.0
nimodipine	vasodilator	17686	9612	-0.7	0.0
imiquimod	antiviral	14234	6044	-0.7	0.0
idoxuridine, idoxuridine ((+)-5-iodo-2'-deoxyuridine)	antiviral	14236	6046	-0.7	0.0
canrenone	diuretic	14536	6905	-0.8	0.0
aurosodium thiomalate		17783	9315	-0.9	0.0
chloramphenicol succinate, chloramphenicol pantothenate complex, chloramphenicol succinate sodium salt	antibiotic	14659	6429	-0.9	0.0
difluprednate	antiinflammatory	17785	9317	-0.9	0.0
aztreonam	antibiotic	14252	6062	-1.0	0.0

dichlorophen, dichlorophene, dichlorophen (2,2 methylene bis 4-chlorophenol)	anthelminithic	14207	6071	-1.0	0.0
tenoxicam	antiinflammatory	17793	9325	-1.0	0.0
oxfendazole	anthelminithic	14939	7296	-1.0	0.0
disodium adenosine triphosphate, adenosine triphosphate disodium, adenosine 5'-(_,_-imido)triphosphate tetralithium, adenosine triphosphate disodium (atp)	vitamin	17406	9038	-1.1	0.0
hydrocodone, hydrocodone polistirex, hydrocodone polistirex/chlorpheniramine polistirex	antitussive	14402	6641	-1.1	0.0
fexofenadine, fexofenadine hydrochloride	antihistaminic	14406	6645	-1.2	0.0
hexetidine	antiseptic	14670	6414	-1.2	0.0
sulfisomidine	antibiotic	14680	6450	-1.2	0.0
carbenicillin, carbenicillin disodium	antibiotic	14268	6078	-1.3	0.0
fenspiride, fenspiride hydrochloride	bronchodilator	17427	9059	-1.3	0.0
g-aminobutyric acid, piperidic acid, gamma-aminobutyric acid, aminobutyricacid(gaba), gaba (gamma-amino-n-butyric acid), 4-aminobutyric acid (gaba)	antihypertensive, nootropic	14572	6941	-1.4	0.0
hydroflumethiazide	antihypertensive	14414	6117	-1.4	0.0
acemetacin	antiinflammatory	17828	9360	-1.4	0.0
chlorphenoxamine, chlorphenoxamine hydrochloride	antihistaminic	14422	6661	-1.4	0.0

adrenalone, adrenalone hydrochloride	hemostatic	14579	6948	-1.5	0.0
dobutamine, dobutamine hydrochloride	cardiotonic	16571	7838	-1.5	0.0
caffeic acid		15893	7924	-1.5	0.0
aminohippurate, aminohippuric acid, aminohippuric acid (p-aminohippuritic acid)	diagnostic aid	14742	7278	-1.6	0.0
buspirone, buspirone hydrochloride	anxiolytic	16578	7845	-1.6	0.0
doxylamine, doxylamine succinate, doxylamine succinate salt	antihistaminic	14433	6672	-1.6	0.0
chloramphenicol palmitate	antibiotic	14708	6478	-1.7	0.0
bemesetron	antiemetic	16416	8563	-1.7	0.0
ezetimibe	antihyperlipidemic	14784	6782	-1.7	0.0
ursolic acid	n/a	14904	5722	-1.8	0.0
dopamine, dopamine hydrochloride, 3-hydroxytyramine (dopamine), dopamine (3-hydroxytyramine hydrochloride)	cardiotonic	14789	6787	-1.8	0.0
santonin, santonin'alpha-	anthelminithic	14259	6123	-1.9	0.0
esmolol, esmolol hydrochloride	antiarrhythmic	14796	6794	-1.9	0.0
oxandrolone	steroid, androgen	14461	4733	-2.0	0.0
ciclopirox olamine, ciclopirox ethanolamine	antifungal	14310	6120	-2.0	0.0
mebrofenin, mebrofenin ((3-bromo-2,4,6-trimethylphenylcarbamoyl) methyliminodiacetic acid)	diagnostic aid	14771	7307	-2.0	0.0
propionic acid, propionic acid sodium salt	antifungal	14315	6125	-2.0	0.0

decitabine, 5-aza-2'-deoxycytidine, decitabine (5-aza-2-deoxycytidine)	antineoplastic	17896	9428	-2.1	0.0
norephedrine, phenylpropanolamine, cathine, phenylpropanolamine hydrochloride, 1s,2r-phenylpropanolamine hydrochloride, norephedrine hydrochloride (phenylpropanolamine), 1r,2s norphedrine	decongestant, anorexic, decongestant (nasal)	14473	6712	-2.2	0.0
pargyline, pargyline hydrochloride, pargyline (n-methyl-n-propargylbenzylamine)	antihypertensive	14463	6166	-2.2	0.0
zinc peroxide, medicinal, zinc peroxide	antiseptic	14734	6478	-2.2	0.0
caramiphen edisylate 10mg, caramiphen, caramiphen edisylate, caramiphen hydrochloride	antitussive	14476	6715	-2.2	0.0
domiphen bromide	antibacterial	14284	6148	-2.3	0.0
cefpiramide, cefpiramide sodium	antibiotic	14749	6519	-2.3	0.0
diphenidol, diphenidol hydrochloride, difenidol hydrochloride (diphenidol)	antiemetic	14876	5322	-2.4	0.0
gamma-terpineol 1gm/100ml, terpineol, terpineol mixed isomers	therapeutic plant extract	14748	6492	-2.4	0.0
enalaprilat	antihypertensive	14484	6187	-2.6	0.0
aminopyridine	nootropic	15681	7605	-2.6	0.0
iodate, sodium iodate 0.0472%, potassium iodate	antiseptic	14761	6505	-2.6	0.0
chicago sky blue 6b		15982	8013	-2.7	0.0
cefixime	antibiotic	14354	6164	-2.7	0.0

carbenoxolone, carbenoxolone sodium, carbenoxolone disodium salt	antiulcerative	17882	9808	-2.7	0.0
carbon tetrachloride, carbon tetrachloride, chromasolv for hplc	anthelminthic, anthelminithic	14310	6174	-2.7	0.0
potash, sulfurated, potash sulfurated	antiseptic	14310	6174	-2.7	0.0
niflumic-acid, niflumic acid	antiinflammatory	17952	9484	-2.7	0.0
triple dye (brilliant green fw 482.64 gentian violate fw 408 proflavine hemisulfate fw 258.29), triple dye (brilliant green fw 482.64, gentian violate fw 408, proflavine hemisulfate fw 258.29)	antiseptic	14767	6511	-2.7	0.0
ajmaline	antihypertensive	14667	7036	-2.7	0.0
isoniazid, isoniazid (isonicotinic acid hydrazide)	antibacterial, antibiotic	14312	6176	-2.7	0.0
diclazuril	antibacterial	15067	7424	-2.8	0.0
fenticlor, fenticlor (bis(2-hydroxy-5-chlorophenyl) sulfide)	antibacterial	14317	6181	-2.8	0.0
alcohol, ethanol	antidote, antidote (methanol poisoning)	14833	7369	-2.8	0.0
triclosan, triclosan (5-chloro-2-(2,4-dichloro-phenoxy)phenol)	antiseptic	14780	6524	-2.9	0.0
benznidazole, benznidazole (n-benzyl-2-nitro-1h-imidazole-1-acetamide)	antiprotozoal	15078	7435	-2.9	0.0
lithium salicylate	antipsychotic	15402	5575	-2.9	0.0
gramine		16007	8038	-3.0	0.0

metrifonate, trichlorfon, metrifonate ((2,2,2-trichloro-1-hydroxyethyk)phosphonic acid dimethyl ester)	anthelminithic	15081	7438	-3.0	0.0
torsemide	diuretic	14869	6867	-3.0	0.0
oxaceprol, oxaceprol (trans-1-acetyl-4-hydroxy l-proline)	antiinflammatory	17984	9516	-3.1	0.0
perflubron, perflubron (perfluorooctyl bromide)	diagnostic aid	14852	7388	-3.1	0.0
theophylline, theophylline monohydrate	bronchodilator	14534	6773	-3.1	0.0
methoxyphenamine, methoxyphenamine hydrochloride	bronchodilator	14535	6774	-3.1	0.0
disopyramide	antiarrhythmic	14878	6876	-3.2	0.0
oxeladin, oxeladin citrate salt	antitussive	17599	9231	-3.2	0.0
iodoform	antibacterial	14345	6209	-3.3	0.0
atenolol, s(-)-atenolol, esatenolol, atenolol'(r)-(+)-, atenolol'(s)-(-)-	antihypertensive, adrenergic	14529	6232	-3.3	0.0
mephentermine, mephentermine sulfate, mephentermine hemisulfate	antihypotensive	14533	6236	-3.4	0.0
xylazine	anesthetic, sedative	17947	9873	-3.4	0.0
dienestrol	estrogen	17949	9875	-3.4	0.0
metaraminol, metaraminol bitartrate	antihypotensive	14536	6239	-3.4	0.0
asparaginase		14905	5651	-3.4	0.0
canrenoate, canrenoate potassium, canrenoic acid potassium salt, canrenoic acid, potassium salt, potassium canrenoate	diuretic	14715	7084	-3.4	0.0

aluminum potassium sulfate		14932	5378	-3.5	0.0
sulfisoxazole	antibiotic	14824	6594	-3.5	0.0
tocainide, tocainide hydrochloride	antiarrhythmic	14903	6901	-3.5	0.0
oxprenolol, oxprenolol hydrochloride	antihypertensive	14547	6250	-3.6	0.0
nifursol	antiprotozoal	15126	7483	-3.6	0.0
fluspirilene, fluspirilen, flurandrenolide	antipsychotic	15761	7685	-3.7	0.0
cefmetazole, cefmetazole sodium salt, cefmetazole sodium	antibiotic	14414	6224	-3.7	0.0
cholestyramine, colestyramine, cholestyramine resin	antihyperlipidemic	14918	6916	-3.8	0.0
naphthalene	anthelminithic	14377	6241	-3.8	0.0
candesartan cilexetil, candesartan cilextil	antihypertensive., antihypertensive	14568	6271	-3.9	0.0
quinpirole, quinpirole hydrochloride, quinpirole hydrochloride'(-)-	antihypertensive	15026	5844	-4.0	0.0
nevirapine	antiviral	14433	6243	-4.0	0.0
isopropyl alcohol	antiseptic	14850	6594	-4.0	0.0
oltipraz	antiviral	15159	7516	-4.1	0.0
mercaptopurine	antineoplastic	18017	9943	-4.1	0.0
labetalol, dilevalol, dilevalol hydrochloride, labetalol hydrochloride, labetalol also 149 (2 hydroxy 5 (1 hydroxy-2((1 methyl-3phenylpropyl)), labetalol (2 hydroxy 5 (1 hydroxy-2((1 methyl-3phenylpropyl))	antihypertensive	14581	6284	-4.2	0.0
hydroxyalprazolam, 4-hydroxy-alprazolam, a-hydroxyalprazolam	anxiolytic	15805	7729	-4.2	0.0
glycocholic acid		16106	8137	-4.2	0.0
amifostine	antineoplastic	14953	5699	-4.3	0.0

mebendazole	anthelminithic	14408	6272	-4.3	0.0
chondroitin sulfate a, chondroitin sulfate sodium	antihyperlipidemic	14778	7147	-4.4	0.0
echinacea juice		14410	6274	-4.4	0.0
amikacin, amikacin hydrate	antibiotic	14456	6266	-4.4	0.0
isocarboxazid	antidepressant	15484	5657	-4.5	0.0
norfenefrine, norfenefrine (norphenylephrine hydrochloride)	antihypotensive	14785	7154	-4.5	0.0
pyrilamine maleate, pyrilamine, pyrilamine maleate (mepyramine maleate)	antihistaminic	14626	6865	-4.5	0.0
terbinafine, terbinafine hydrochloride	antifungal	14467	6277	-4.6	0.0
oxiconazole, oxiconazole nitrate	antifungal	14468	6278	-4.6	0.0
butyl chloride, butyl chloride (1-chlorobutane)	anthelminithic	15200	7557	-4.6	0.0
mefexamide, mefexamide hydrochloride	nootropic	18075	10001	-4.7	0.0
captan 0.1%w/v, captan	phamaceutic aid	14901	6645	-4.9	0.0
levopropoxyphene, levopropoxyphene napsylate	antitussive	14650	6889	-4.9	0.0
thallium	diagnostic aid	14982	7518	-4.9	0.0
silibinin, milk thistle, silymarin, silibinin (silybin)	hepatic protectant, therapeutic plant extract, misc-hepatoprotectant	18154	9686	-4.9	0.0
diethylcarbamazine, diethylcarbamazine citrate	anthelminithic	16836	8103	-4.9	0.0
quinapril, quinapril hydrochloride	antihypertensive	14630	6333	-5.0	0.0
ferric chloride, ferric chloride anhydrous, iron(iii) chloride	antiseptic	14913	6657	-5.0	0.0
alpha-ketoglutaric acid		15090	5908	-5.1	0.0

phenylalanine, d-phenylalanine	nutrient	16177	8208	-5.2	0.0
nocodazole	antineoplastic	16185	8216	-5.3	0.0
pralidoxime, pralidoxime chloride, pralidoxime (pyridine 2-aldoximemethochloride), pralidoxime (2-pyridinealdoxime methiodide)	antidote	15009	7545	-5.3	0.0
tolnaftate	antifungal	14511	6321	-5.3	0.0
bacitracin, bacitracin zinc salt	antibiotic, antibacterial	14511	6321	-5.3	0.0
folic acid	vitamin	18132	10058	-5.3	0.0
ceftibuten	antibiotic	14944	6714	-5.4	0.0
gefitinib, gefitinib (iressa)	antineoplastic	15016	5762	-5.4	0.0
famotidine	antiulcerative	16877	8144	-5.5	0.0
vitamin e nicotinate		15112	5930	-5.5	0.0
dropropizine, levodropropizine, dropropizine (r,s)	antitussive	17806	9438	-5.6	0.0
veratrole 2.40gm, veratrole	antiseptic, pharmaceutic aid	14947	6691	-5.6	0.0
acetophenazine	antipsychotic	15546	5719	-5.6	0.0
mercaptopurinemonohydrate, 6-mercaptopurine monohydrate	antineoplastic	15030	5776	-5.7	0.0
basic fuchsin, basic fuchsin hydrochloride	diagnostic aid	15040	7576	-5.7	0.0
methotrimeprazine, levomepromazine, methotrimeprazine maleat salt	analgesic	12987	4509	-5.7	0.0
perchlorate	antiseptic	14958	6702	-5.8	0.0
ampicillin, ampicillin sodium, ampicillin trihydrate, ampicillin sodium salt	antibiotic	14539	6349	-5.8	0.0

cefamandole, cefamandole sodium salt, cefamandole sodium	antibiotic	14542	6352	-5.8	0.0
dihydrostreptomycin, dihydrostreptomycin sulfate	antibacterial	16906	8173	-5.8	0.0
pyridoxal calcium phosphate, pyridoxal phosphate, pyridoxal-5-phosphate	vitamin	17831	9463	-5.8	0.0
strontium	antineoplastic	15038	5784	-5.9	0.0
levalbuterol hydrochloride, levalbuterol	bronchodilator	14714	6953	-5.9	0.0
isoetharine, isoetharine mesylate, isoetharine mesylate salt	bronchodilator	14714	6953	-5.9	0.0
dexamethasone sodium phosphate	glucocorticoid	14646	4918	-5.9	0.0
zileuton	antiinflammatory	14719	6958	-5.9	0.0
ampicillin, ampicillin sodium, ampicillin trihydrate, ampicillin sodium salt	antibiotic	14553	6363	-6.0	0.0
lopinavir, lopinavir/ritonavir	antiviral	14554	6364	-6.0	0.0
cefazolin, cefazolin sodium salt, cefazolin sodium	antibiotic	14554	6364	-6.0	0.0
cephalothin, cephalothin sodium, cephalothin sodium salt	antibiotic	14985	6755	-6.0	0.0
thyropropic acid, thyropropic acid (3,3',5-triiodothyropropionic acid)	antihyperlipidemic	14893	7262	-6.0	0.0
nicergoline	vasodilator	14893	7262	-6.0	0.0
bismuth subcarbonate, bismuth subcarbonate (bismuth carbonate oxide)	antacid	15066	5512	-6.1	0.0
semustine	antineoplastic	18261	9793	-6.1	0.0
magnesium sulfate	anticonvulsant	15572	5745	-6.1	0.0

tetracaine, tetraca•ne hydrochloride, tetracaine hydrochloride	anesthetic	16926	8193	-6.1	0.0
aluminum clofibrate, clofibric acid, clofibric acid (2-(4-chlorophenoxy)-2-methylpropionic acid)	antihyperlipidemic	14900	7269	-6.1	0.0
eucatropineucatropine hydrochloride	mydriatic	14902	7271	-6.2	0.0
iohexol, iohexol (histodenz (5-(n-2-3-dihydroxypropyl-acetimido)-2,4,6-triiodo-n,n'-bis-(2,3-dihydroxypropyl)-isophthalamide))	diagnostic aid	15073	7609	-6.2	0.0
pralidoxime, pralidoxime chloride, pralidoxime (pyridine 2-aldoximemethochloride), pralidoxime (2-pyridinealdoxime methiodide)	antidote	15076	7612	-6.2	0.0
dexefaroxan, efaroxan, efaroxan (2-ethyl-2-[imidazolin-2-yl]-2,3-dihyrdobenzofuran) hydrochloride	antihypertensive	14909	7278	-6.3	0.0
lofexidine hydrochloride, lofexidine	antihypertensive	14710	6413	-6.3	0.0
ceftizoxime, ceftizoxime sodium	antibiotic	15004	6774	-6.3	0.0
moxisylyte, thymoxamine hcl, moxisylyte hydrochoride, moxisylyte hydrochloride	vasodilator	14915	7284	-6.4	0.0
dimethicone 350, dimethicone, dimethicone (dimethylpolysiloxane)	antiflatulent	18293	9825	-6.4	0.0
pyrantel, pyrantel pamoate	anthelminithic	14535	6399	-6.5	0.0

cefoperazone, cefoperazone sodium, cefoperazone dihydrate, cefoperazone sodium salt	antibiotic	14581	6391	-6.5	0.0
diclofenac, diclofenac sodium, diclofenac sodium salt, sodium dichlofenac	antiinflammatory	14671	4943	-6.5	0.0
pyridoxamine, pyridoxamine phosphate, pyridoxamine, dihydrochloride	vitamin	17889	9521	-6.5	0.0
diethylstilbestrol dipropionate	antineoplastic	15074	5820	-6.5	0.0
tetraethylammonium chloride, tetraethylammonium chloride hydrate	antiseptic	15007	6751	-6.5	0.0
clonidine, clonidine hydrochloride	antihypertensive	14730	6433	-6.6	0.0
myricetin		16296	8327	-6.7	0.0
foscarnet, foscarnet sodium	antiviral	16980	8247	-6.8	0.0
tegafur, tegafur (ftorafur)	antineoplastic	18328	9860	-6.8	0.0
roxarsone, roxarsone (alginic acid (4-hydroxy-3-nitrobenzenearsonic acid))	antibacterial	14558	6422	-6.8	0.0
monophosphothiamine, monophosphothiamine (thiamine monophosphate chloride)	vitamin	17929	9561	-6.9	0.0
pentetic acid, pentetate trisodium	diagnostic aid	15196	6014	-7.0	0.0
merbromin	antibacterial	17000	8267	-7.1	0.0
meglumine diatrizoate, diatrizoate meglumine, diatrizoic acid	diagnostic aidiagnostic aid (radiopaque medium).	15140	7676	-7.1	0.0
carbetapentane citrate	antitussive	17005	8272	-7.1	0.0

penciclovir	antiviral	14622	6432	-7.2	0.0
escitalopram oxalate	antidepressant	15631	5804	-7.2	0.0
aceglutamide, aceglutamide aluminum, aceglutamide (n-acetyl-l-glutamine)	antiulcerative, nootropic	17952	9584	-7.2	0.0
sodium butyrate, sodium butyrate (n-butyric acid)	nutrient	17952	9584	-7.2	0.0
sodium borate	antiseptic	15050	6794	-7.2	0.0
diatrizoate, diatrizoate sodium, sodium diatrizoate	diagnostic aid	15150	7686	-7.3	0.0
sodium hypochlorite, sodium hypochlorite solution	antiseptic	15056	6800	-7.3	0.0
uracil	antineoplastic	17969	9601	-7.4	0.0
losartan, losartan potassium	antihypertensive	14777	6480	-7.4	0.0
trientine, trientine hydrochloride, trientine (triethylenetetramine)	antidote	15161	7697	-7.4	0.0
thiodiglycol	antineoplastic	18334	10260	-7.4	0.0
mizoribine	immunosuppressant	18393	9925	-7.5	0.0
desoxycorticosterone acetate, desoxycorticosterone acetate (11-deoxycorticosterone acetate)	steroid	14719	4991	-7.5	0.0
harmaline hydrochloride dihydrate		16362	8393	-7.5	0.0
toremifene, toremifene citrate, toremiphene citrate	antineoplastic	15134	5880	-7.6	0.0
hesperidin	therapeutic plant extract	16370	8401	-7.6	0.0
trans anethole, anethole, trans-anethole	antitussive	14831	7070	-7.7	0.0
ascorbic acid 6-palmitate		18001	9633	-7.7	0.0

nicotine, nicotine ditartrate	nootropic	15662	5835	-7.8	0.0
halcinonide	antiinflammatory	14730	5002	-7.8	0.0
fluphenazine, flufenazine, fluphenazine hydrochloride, fluphenazine dihydrochloride	antipsychotic	15663	5836	-7.8	0.0
iodide, potassium iodide	antifungal	14659	6469	-7.8	0.0
pheniramine, pheniramine maleate	antihistaminic	14841	7080	-7.8	0.0
fusaric acid		16387	8418	-7.8	0.0
roxatidine acetate, roxatidine acetate hydrochloride, roxatidine acetate hcl	antiulcerative	18011	9643	-7.9	0.0
disofenin, disofenin (n-(2,6-diisopropylphenyl-carbamoylmethyl)-iminodiacetic acid)	diagnostic aid	15195	7731	-7.9	0.0
sisomicin, pentisomicin, sisomicin sulfate	antibiotic, antibacterial	17068	8335	-7.9	0.0
toltrazuril	antibacterial	15449	7806	-8.1	0.0
clenbuterol, clenbuterol hydrochloride	bronchodilator	18398	10324	-8.1	0.0
yohimbine hydrochloride, yohimbine	erectile dysfunction	18403	10329	-8.2	0.0
arginine glutamate, l-arginine l-glutamate salt	antidote	15215	7751	-8.2	0.0
octabenzone, octabenzone (2-hydroxy-4-(octyloxy)-benzophenone) (octabenzone)	dermatologic	18041	9673	-8.2	0.0
giberellic acid		16417	8448	-8.2	0.0
phenothrin	dermatologic	18413	10339	-8.3	0.0
croton oil		15183	5629	-8.3	0.0
felbinac, felbinac (4-biphenylacetic acid)	antiinflammatory	18469	10001	-8.3	0.0
bezafibrate	antihyperlipidemic	15051	7420	-8.4	0.0

timolol, timolol maleate, timolol maleate salt	antihypertensive	14835	6538	-8.4	0.0
nitroglycerin, nitroglycerine	antianginal	14837	6540	-8.4	0.0
cinnarizine, cinnarazine	antihistaminic	18433	10359	-8.5	0.0
simvastatin	antihyperlipidemic	17110	8377	-8.5	0.0
nifedipine	antianginal	14843	6546	-8.5	0.0
benzetimide, dexetimide, benzetimide hydrochloride	antiparkinsonian, antihypertensive	16121	8045	-8.5	0.0
methionine sulfoximine (l)		16442	8473	-8.5	0.0
cromolyn, cromolyn sodium, cromolyn disodium salt	antiasthmatic, bronchodilator	14890	7129	-8.6	0.0
dicloxacillin, dicloxacillin sodium, dicloxacillin sodium salt	antibiotic	17117	8384	-8.6	0.0
aloe, aloe vera oil		16446	8477	-8.6	0.0
norethindrone acetate	progestogen	15288	6106	-8.6	0.0
felbamate	anticonvulsant	15712	5885	-8.7	0.0
shikimic acid	n/a	15291	6109	-8.7	0.0
octopamine, octopamine hydrochloride'(+,-)-, octopamine hydrochloride	antihypotensive	18456	10382	-8.7	0.0
chloropyramine, chloropyramine hydrochloride	antihistaminic	18091	9723	-8.7	0.0
pirenperone	antidote	18459	10385	-8.8	0.0
zinc carbonate	astringent	18093	9725	-8.8	0.0
hydroxyzine, hydroxyzine hydrochloride, hydroxyzine dihydrochloride, hydroxyzine (dihydrochloride)	anxiolytic	15719	5892	-8.8	0.0
antipyrine, antipyrine, 4-hydroxy	antiinflammatory	17135	8402	-8.8	0.0
chlorpropamide	antidiabetic	17137	8404	-8.8	0.0
suxibuzone	antiinflammatory	18518	10050	-8.9	0.0

folinic acid, folinic acid calcium salt, folinic acid calcium salt pentahydrate		16468	8499	-8.9	0.0
sulfachlorpyridazine, sulfachloropyridazine	antibiotic	15508	7865	-8.9	0.0
betamethasone sodium phosphate, betamethazone sodium phosphate	glucocorticoid	14784	5056	-8.9	0.0
caffeine citrate, caffeine, citrated	nootropic	14682	4909	-9.0	0.0
cytarabine, cytarabine (cytosine 1-b-d-arabinofuranoside), cytidine	antineoplastic	15213	5959	-9.1	0.0
dextroamphetamine		16482	8513	-9.1	0.0
chlorate, potassium chlorate unk	sedative	15172	6916	-9.1	0.0
tolonidine, tolonium chloride, tolonium chloride (toluidine blue o)	hemostatic, antihypertensive	15280	7278	-9.2	0.0
benzoylpas calcium, benzoylpas	antibacterial	14746	6556	-9.2	0.0
sulfamethoxypyridazine	antibiotic	17178	8445	-9.4	0.0
ethionamide	antibacterial	17185	8452	-9.5	0.0
meglutol, meglutol (3-hydroxy-3-methylglutaric acid)	antihyperlipidemic	15128	7497	-9.5	0.0
beclomethasone dipropionate	antiinflammatory, glucocorticoid	16519	8550	-9.5	0.0
cefotiam, cefotiam hydrochloride	antibiotic	14766	6576	-9.6	0.0
metoclopramide, metoclopramide hydrochloride, metoclopramide monohydrochloride, metoclopromide	antiemetic	15248	5694	-9.6	0.0
aluminum hydroxide	antacid	15253	5699	-9.7	0.0

ceforanide	antibiotic	14778	6588	-9.8	0.0
salicylanilide	antifungal	14781	6591	-9.8	0.0
terpin, terpin hydrate, terpin (p-menthane-1,8-diol monohydrate)	expectorant	14975	7214	-9.8	0.0
gabob, aminohydroxybutyric acidl-gamma-amino-beta-hydroxybutyric acid	anticonvulsant	16223	8147	-9.9	0.0
selenomethionine, seleno-d, l-methionine	diagnostic aid	15338	7874	-9.9	0.0
fosphenytoin, fosphenytoin sodium	anticonvulsant	15778	5951	-9.9	0.0
chloranil 0.5%, chloranil	antifungal	17222	8489	-9.9	0.0
carbocloral	sedative	14725	4952	-9.9	0.0
crospovidone, povidone iodine, povidone, povidone-iodine, povidone iodine (polyvinylpyrrolidone-iodine complex)	antibacterial, phamaceutic aid, antiseptic	15225	6969	-10.0	0.0
azithromycin, azithromycin dihydrate	antibiotic	14793	6603	-10.0	0.0
guaifenesin, guaifenesen (guaiacol glyceryl ether)	expectorant	14987	7226	-10.0	0.0
praziquantel	anthelminithic	14753	6617	-10.1	0.0
etoposide	antineoplastic	17235	8502	-10.1	0.0
bupropion, bupropion hydrochloride	antidepressant	15793	5966	-10.2	0.0
lactitol, maltitol, lactitol monohydrate d-, lactitol monohydrate	laxative, phamaceutic aid	18220	9852	-10.2	0.0
prasterone, dehydroepiandrosterone, prasterone (dehydroisoandrosterone), prasterone (dhea, dehydroepiandrosterone)	steroid	18643	10175	-10.2	0.0
papaverine, papaverine hydrochloride	vasodilator	18604	10530	-10.3	0.0

proxyphylline, proxyphylline (17-beta-hydroxypropyltheophylline)	bronchodilator	18235	9867	-10.4	0.0
carmustine	antineoplastic	15291	6037	-10.5	0.0
quillaja (saponin), saponin	therapeutic plant extract	15292	6038	-10.5	0.0
cephradine	antibiotic	15273	7043	-10.6	0.0
alginic acid, alfuzocin	hemostatic	15371	7369	-10.6	0.0
bismuth iii oxide, bismuth(iii) oxide	astringent	15299	5745	-10.6	0.0
xylose, l-xylose, d-xylose	diagnostic aid	15387	7923	-10.6	0.0
rebamipide	antiulcerative	18259	9891	-10.6	0.0
chlorambucil, phenylbutyric acid (chlorambucil), phenylbutyrate sodium	antineoplastic	15302	6048	-10.7	0.0
phenylbutazone	antiinflammatory	14867	5139	-10.7	0.0
trimebutine	antispasmodic	16286	8210	-10.7	0.0
topiramate	anticonvulsant	15825	5998	-10.8	0.0
ibuprofen, dexibuprofen, ibuprofen's-(+)-	antiinflammatory, analgesic	14879	5151	-11.0	0.0
tetrahydrozoline, tetrahydrozoline hydrochloride	decongestant	17302	8569	-11.0	0.0
metrizoic acid, metrizoic acid 610.4mg	diagnostic aid (radiopaque medium)., diagnostic aid	15419	7955	-11.0	0.0
hydralazine, hydralazine hydrochloride	antihypertensive	14995	6698	-11.0	0.0
lithium bromide	antipsychotic	15842	6015	-11.1	0.0
diazoxide	antihypertensive	14999	6702	-11.1	0.0
tetramisole, tetramisole hydrochloride	anthelminithic	15672	8029	-11.2	0.0
cefadroxil	antibiotic	17322	8589	-11.2	0.0
colchicine	anticholelithogenic	13771	5342	-11.3	0.0
oxolamine	antiinflammatory	18740	10272	-11.3	0.0
tetrachloroethylene	anthelminithic	15695	8052	-11.5	0.0

amantadine, amantadine hydrochloride, amantadine hydrochloride (1-adamantanamine hydrochloride)	antiviral	14883	6693	-11.5	0.0
amphotericin, amphotericin b	antifungal	14883	6693	-11.5	0.0
orphenadrine citrate, orphenadrine	muscle relaxant (skeletal)	13234	4756	-11.5	0.0
2,4-dinitroanisole	antiseptic	15328	7072	-11.6	0.0
capsicum oleoresin, zucapsaicin, capsaicin, civamide, oleoresins capsicum	analgesic, dermatologic	16353	8277	-11.6	0.0
chlorogenic acid		16684	8715	-11.7	0.0
carbocloral	sedative	14803	5030	-11.7	0.0
bismuth aluminate	antacid	18357	9989	-11.7	0.0
indomethacin	antiinflammatory	17362	8629	-11.7	0.0
silver nitrate	antibacterial	14853	6717	-11.7	0.0
hydroxyurea	antineoplastic	15362	6108	-11.8	0.0
pirenzepine, pirenzepine hydrochloride, pirenzepine dihydrochloride	antiulcerative	18752	10678	-11.8	0.0
erythromycin ethylsuccinate	antibiotic	17369	8636	-11.8	0.0
serotonin, serotonin hydrochloride, serotonin (hydroxytryptamine)		16712	8743	-12.0	0.0
citrate dextrose solution (acd), citrate-dextrose solution (acd)	anticoagulant	15302	7671	-12.0	0.0
flavin adenin dinucleotide	vitamin	18395	10027	-12.1	0.0
fomocaine	anesthetic	16395	8319	-12.2	0.0
tartrate tetrahydrate, tartratetetrahydrate	laxative	15385	5831	-12.2	0.0
taurine	nutrient	18401	10033	-12.2	0.0
acefylline piperazine, theophylline 7 acetic acid, theophylline-7-acetic acid	bronchodilator	18402	10034	-12.2	0.0

proadifen, proadifen hydrochloride, proadifen hydrochloride [skf-525	antidiabetic	18797	10723	-12.3	0.0
quipazine maleate, quipazine dimaleate salt	antidepressant	18800	10726	-12.3	0.0
baclofen, l-baclofen, baclofen (r,s)	muscle relaxant (skeletal)	18808	10734	-12.4	0.0
benzaldehyde	pharmaceutic aid, phamaceutic aid	15338	7707	-12.5	0.0
carbofenotion, carbophenothion	dermatologic	18432	10064	-12.6	0.0
bepridil, bepridil hydrochloride	antianginal	15088	6791	-12.6	0.0
aluminum ammonium sulfate dodecahydrate		15407	5853	-12.6	0.0
metoprolol, metoprolol-(+,-) (+)-tartrate salt, metoprolol tartrate	antihypertensive	17431	8698	-12.6	0.0
aminocaproic acid, 6-aminocaproic acid	hemostatic	15514	7512	-12.7	0.0
durapatite, durapatite (hydroxyapatite)	nutrient	18445	10077	-12.7	0.0
bitoscanate, bitoscanate (1,4 phenylene disothiocyanate)	anthelminithic	15789	8146	-12.8	0.0
verteporfin	antineoplastic	14854	5081	-12.8	0.0
triflupromazine, triflupromazine hydrochloride	antipsychotic	17444	8711	-12.8	0.0
hydrastinine, hydrastinine hydrochloride	cardiotonic	15357	7726	-12.8	0.0
hexamethonium chloride	antihypertensive	15105	6808	-12.8	0.0
prednisolone acetate, prednisone acetate, prednisolone acetate opthalmic solution	glucocorticoid., glucocorticoid	14974	5246	-13.0	0.0
ethyl vanillate ng, ethyl vanillate	antiseptic	15420	7164	-13.0	0.0

guaiacolsulfonate, potassium guaiacolsulfonate, guaiacolsulfonic acid potassium salt	expectorant	15187	7426	-13.1	0.0
cyclophosphamide, cyclophosphamide hydrate	antineoplastic	17465	8732	-13.1	0.0
cladribine	antineoplastic	15440	6186	-13.2	0.0
ursodiol, chenodiol, urosiol, ursodiol (ursodeoxycholic acid)	anticholelithogenic	13865	5436	-13.2	0.0
nandrolone decanoate	steroid	14985	5257	-13.2	0.0
zinc chloride	antiseptic	15441	5887	-13.3	0.0
procyclidine, procyclidine hydrochloride	antiparkinsonian	15965	6138	-13.3	0.0
benzoquinonium chloride, benzoquinonium dibromide	antiseptic	15444	7188	-13.4	0.0
mitobronitol, mitolactol, mitobronitol (1,6-dibromo-1,6-dideoxy-d-mannitol)	antineoplastic	18946	10478	-13.5	0.0
calcium bromide	sedative	14887	5114	-13.5	0.0
itraconazole, itraconazole also 1974	antifungal	15008	6818	-13.6	0.0
hydrocortisone hemisuccinate, hydrocortisone hemisuccinate monohydrate	glucocorticoid	15001	5273	-13.6	0.0
tulobuterol	bronchodilator	18533	10165	-13.7	0.0
tiapride, tiapride hydrochloride	antispasmodic	16506	8430	-13.7	0.0
canavanine sulfate monohydrate (l,+)		16844	8875	-13.7	0.0
muramic acid, n-acetyl		16846	8877	-13.7	0.0
castile soap		15470	7214	-13.8	0.0
prednisolone sodium phosphate	glucocorticoid., glucocorticoid	15012	5284	-13.8	0.0

prilocaine, prilocaine hydrochloride	anesthetic	17523	8790	-13.8	0.0
triethylenemelamine	antineoplastic	15479	6225	-13.9	0.0
doxepin, doxepin hydrochloride	antidepressant	15997	6170	-13.9	0.0
benzyl nicotinate	dermatologic	18555	10187	-13.9	0.0
clopamide	antihypertensive	15436	7805	-14.0	0.0
castor oil		15481	5927	-14.1	0.0
gemcitabine	antineoplastic	15489	6235	-14.1	0.0
selegiline, selegiline hcl, selegiline hydrochloride, selegiline (deprenyl r-(-))	antiparkinsonian	16007	6180	-14.1	0.0
furfurylaminopurine'6-, kinetin		16891	8922	-14.3	0.0
midodrine, midodrine hydrochloride	antihypotensive	17564	8831	-14.4	0.0
sitosterol, sitosterols, beta-sitosterol	steroid	15038	5310	-14.4	0.0
dextran sulfate		15466	7835	-14.4	0.0
eflornithineflornithine hydrochlorideflornithine (dl-alpha-difluoromethylornithine hydrochloride), difluoromethylornithine	antineoplastic	15515	6261	-14.6	0.0
didanosine, dideoxyinosine, didanosine (2'-3'-dideoxyinosine)	antiviral	15077	6887	-14.7	0.0
hexamethonium iodide		16929	8960	-14.8	0.0
acebutolol, acebutolol hydrochloride	antihypertensive	17603	8870	-14.9	0.0
fenoterol	bronchodilator	18642	10274	-14.9	0.0
dextrorphan tartrate	analgesic (narcotic)	16597	8521	-14.9	0.0
aluminum chlorohydrate		15064	5336	-15.0	0.0
bilirubin	n/a	15645	6463	-15.0	0.0

pentylenetetrazol, pentylenetetrazole, pentetrazol, pentylenetetrazol (pentetrazole), pentylenetetrazol (pentetrazol (1,5-pentamethylenetetrazole))	nootropic	14955	5182	-15.0	0.0
sulfadimethoxine	antibiotic	17628	8895	-15.2	0.0
calcipotriene	dermatologic	15079	5351	-15.3	0.0
selegiline, selegiline hcl, selegiline hydrochloride, selegiline (deprenyl r-(-))	antiparkinsonian	16070	6243	-15.3	0.0
digoxigenin		16970	9001	-15.3	0.0
amantadine, amantadine hydrochloride, amantadine hydrochloride (1-adamantanamine hydrochloride)	antiviral	15112	6922	-15.3	0.0
magnesium silicate	phamaceutic aid	15547	5993	-15.3	0.0
theobromine	diuretic	19091	11017	-15.4	0.0
nizatidine	antiulcerative	15562	6008	-15.6	0.0
phytate persodium, phytic acid, phytic acidodecasodium salt hydrate	phamaceutic aid	15684	6502	-15.7	0.0
bismuth subnitrate	antacid	15571	6017	-15.8	0.0
sodium bromide, carbonate, sodium carbonate	sedative, phamaceutic aid	14989	5216	-15.8	0.0
danazol	steroid	15105	5377	-15.8	0.0
nebramycin	antibacterial	17018	9049	-15.9	0.0
homatropine, dl-homatropine	mydriatic	14998	5225	-16.0	0.0

meclofenamic acid, meclofenamate, meclofenamate sodium, meclofenamic acid sodium salt monohydrate, meclofenamic acid sodium salt, sodium meclofenamate	antiinflammatory	15113	5385	-16.0	0.0
glutamic acidl-glutamic acid monohydrate, l-glutamic acid sodium salt hydrate	anticonvulsant, nutrient	16110	6283	-16.0	0.0
etodolac, r-etodolac	antiinflammatory	15115	5387	-16.1	0.0
bornyl acetate, bornyl acetate, (-)-	antiseptic	15619	7363	-16.2	0.0
tryptophan		16119	6292	-16.2	0.0
thiourea 2.5, thiourea	antineoplastic	15608	6354	-16.3	0.0
periciazine	antipsychotic	16698	8622	-16.3	0.0
phenoxyethanol, 2-phenoxyethanol	antiseptic	15631	7375	-16.4	0.0
carboplatin, carboplatin (diamine(1,1-cyclobutane-dicarboxylato)platinum(ii))	antineoplastic	15613	6359	-16.4	0.0
nystatin, nystatine	antifungal	15180	6990	-16.5	0.0
akwa tears		15022	5249	-16.5	0.0
scopoletin		17066	9097	-16.5	0.0
vesamicol hydrochloride'l(-)-, vesamicol hydrochloride		17068	9099	-16.6	0.0
nisoxetine, nisoxetine hydrochloride	antidepressant	15743	6561	-16.7	0.0
chlorprothixene, chlorprothixene hydrochloride	antipsychotic	17750	9017	-16.8	0.0
phenytoin, phenytoin sodium, phenytoin (5,5diphenylhydantoin sodium salt)	anticonvulsant	16158	6331	-16.9	0.0

scopolamine, scopolamine hydrobromide, scopolamine hydrochloride, scopolamine (-)	antispasmodic	15040	5267	-16.9	0.0
bithionol	antiseptic	17764	9031	-17.0	0.0
cinromide, cinromide (trans-3-bromo-n-ethyl-cinnamide)	anticonvulsant	16749	8673	-17.0	0.0
ethosuximide	anticonvulsant	16162	6335	-17.0	0.0
adenosine 3',5'-cyclic monophosphate'(-)-		17107	9138	-17.1	0.0
iproniazid, iproniazide phosphate, iproniazid (isonicotinic acid hydrazide), iproniazid phosphate salt	antidepressant	16169	6342	-17.1	0.0
mianserin, mianserin hydrochloride, mianserine hydrochloride	antidepressant	16760	8684	-17.1	0.0
modafinil, armodafinil	nootropic	15051	5278	-17.2	0.0
pimecrolimus	immunosuppresant., immunosuppressant	15170	5442	-17.2	0.0
dicyclomine, dicyclomine hydrochloride	antispasmodic	17792	9059	-17.3	0.0
naproxen, naproxen sodium salt'(r) -, naproxen(+), naproxen (s-6-methoxy methyl 2-naphthalene acetic acid), naproxen sodium, naproxen (+)	antiinflammatory	15175	5447	-17.3	0.0
indinavir	antiviral	15248	7058	-17.6	0.0
paraaminobenzoic acid, aminobenzoic acid, 4-aminobenzoic acid, sodium salt,99%, p-aminobenzoic acid	dermatologic, vitamin, antibacterial	15070	5297	-17.6	0.0
tyloxapol	mucolytic	17159	9190	-17.7	0.0

salicin	analgesic	16812	8736	-17.8	0.0
ivermectin	anthelminithic	15231	7095	-18.0	0.0
cyclopentolate, cyclopentolate hydrochloride	mydriatic	15090	5317	-18.0	0.0
octanoic acid		15829	6647	-18.2	0.0
n acetyl l cysteine, acetylcysteine, n-acetyl-l-cysteine, acetylcysteine (n-acetyl l-cysteine)	misc- mucolytic, expectorant	15527	7766	-18.3	0.0
dextran, dextran 1, dextran 12gm/100ml, dextran from leuconostoc mesenteroides, belladonna extract	plasma volume expander	15434	7137	-18.3	0.0
biotin, d-biotin	vitamin	17206	9237	-18.3	0.0
butacaine, butacaine sulfate	anesthetic	16852	8776	-18.4	0.0
alcloxa	dermatologic	17210	9241	-18.4	0.0
bismuth iii citrate, bismuth(iii) citrate 325 mesh	antacid	15714	6160	-18.5	0.0
bopindolol, bopindolol maleate	antihypertensive	15750	8119	-18.6	0.0
nilutamide	antineoplastic	17889	9156	-18.6	0.0
aloe, aloe vera oil		15721	6167	-18.7	0.0
carbazole 0.34%, carbazole	antiseptic	15784	7528	-18.8	0.0
lomustine	antineoplastic	15745	6491	-18.8	0.0
ammonium carbonate, ammonium bicarbonate	expectorant	15567	7806	-18.9	0.0
famciclovir	antiviral	15327	7137	-18.9	0.0
pimethixene, pimethixene maleate	antipsychotic	17259	9290	-19.0	0.0
bentiromide, bentiromide (n-benzoyl-l-tyrosyl-p-aminobenzoic acid (bentiromide)	diagnostic aid	16000	8536	-19.1	0.0
arsanilic acid, p-arsanilic acid	antibacterial	16248	8605	-19.2	0.0

vardenafil, vardenafil hydrochloride	erectile dysfunction	14156	5727	-19.3	0.0
chlorothymol, chlorothymol (4-chloro-2-isopropyl-5-methylphenol)	antiseptic	15818	7562	-19.3	0.0
bicuculline		17287	9318	-19.4	0.0
vancomycin, vancomycin hydrochloride, vancomycin hcl hydrate	antibiotic	15823	7567	-19.4	0.0
chlorosalicylanilide 0.5%, chlorosalicylanilide, 5-chlorosalicylanilide	antiinflammatory	15826	7570	-19.4	0.0
cyproterone acetate, cyproterone	antineoplastic	19498	11030	-19.5	0.0
clebopride, clebopride maleate	antiemetic	19052	10684	-19.5	0.0
thymol iodide	antiseptic	15835	7579	-19.6	0.0
temozolomide	antineoplastic	15789	6535	-19.6	0.0
sodium taurocholate 0.125gr, taurocholate, sodium taurochlorate hydrate	choleretic	15770	6216	-19.6	0.0
bromide, potassium bromide	sedative	15162	5389	-19.6	0.0
benfluorex, benfluorex hydrochloride	antihyperlipidemic	17312	9343	-19.7	0.0
molindone, molindone hydrochloride	antipsychotic., antipsychotic	16314	6487	-19.8	0.0
glucosamine, glucosamine hydrochloride, d-galactosamine hydrochloride, d-glucosamine 2-sulfate, d-glucosamine sulfate	antiarthritic, antirheumatic	19091	10723	-19.9	0.0
mesalamine, mesalamine (5-aminosalicylic acid)	antiinflammatory	15298	5570	-20.0	0.0
demecarium, demecarium bromide	cholinergic	15186	5413	-20.2	0.0
carbromal	sedative	15194	5421	-20.3	0.0

primidone	anticonvulsant	16351	6524	-20.5	0.0
dorzolamide, dorzolamide hydrochloride	antiglaucoma	15204	5431	-20.6	0.0
cholesterol	pharmaceutic aid, phamaceutic aid	17385	9416	-20.6	0.0
nipecotic acid '(±)-, nipecotic acid		17385	9416	-20.6	0.0
aminoglutethimide	antineoplastic	15848	6594	-20.7	0.0
tazobactam, tazobactam sodium salt	antibiotic	15907	7651	-20.7	0.0
nifekalant	cardiotonic	15970	6788	-20.8	0.0
prasterone, dehydroepiandrosterone, prasterone (dehydroisoandrosterone), prasterone (dhea, dehydroepiandrosterone)	steroid	19620	11152	-20.8	0.0
piroxicam	antiinflammatory	18065	9332	-20.9	0.0
montelukast, montelukast sodium	antiasthmatic., bronchodilator	15700	7939	-20.9	0.0
mycophenolic acid	immunosuppressa nt	15341	5613	-20.9	0.0
aripiprazole	antipsychotic	16379	6552	-21.0	0.0
phenanthrene 0.63%, phenanthrene	antineoplastic	15873	6619	-21.1	0.0
tolazamide	antidiabetic	18090	9357	-21.2	0.0
famprofazone	analgesic	17438	9469	-21.3	0.0
lansoprazole	antiulcerative	18107	9374	-21.4	0.0
dichlorisone-acetate, dichlorisone acetate	dermatologic	15364	5636	-21.4	0.0
carbachol, carbachol (carbamylcholine chloride)	cholinergic	15247	5474	-21.5	0.0
nafronyl, nafronyl oxalate	vasodilator	19684	11610	-21.6	0.0
ethoxazenethoxazene hydrochloridethoxazene (4-(4-ethoxy-phenylazo)-benzene-1,3-diamine)	analgesic	13666	5188	-21.7	0.0

ferrous sulfate, iron sulfate 0.194gm, iron sulfate, iron(ii) sulfate heptahydrate	hematinic	14272	5843	-21.7	0.0
anabasine		17471	9502	-21.7	0.0
flurothyl, flurothyl (2,2,2-trifluoroethyl ether)	nootropic	15257	5484	-21.7	0.0
bifemelane	nootropic	17110	9034	-21.8	0.0
aluminum acetate, aluminum acetate, basic	antiseptic	15888	6334	-21.9	0.0
cotinine, cotinine fumarate, cotinine (-)	antidepressant	17116	9040	-21.9	0.0
3,5-dibromosalicylaldehyde 0.5pc, 3,5-dibromosalicylaldehyde	antiseptic	15990	7734	-22.0	0.0
rimexolone	antiinflammatory	15393	5665	-22.0	0.0
oxyphenonium, oxyphenonium bromide	antispasmodic	13688	5210	-22.2	0.0
potassium ricinoleate unk, ricinoleate	unclassified	14303	5874	-22.3	0.0
bromopride	antiemetic	19318	10950	-22.5	0.0
lithocholic acid		17536	9567	-22.6	0.0
mirtazapine	antidepressant	16471	6644	-22.7	0.0
ethamivan, ethamivan (vanillic acid diethylamide)	nootropic	15301	5528	-22.7	0.0
azacitidine, fazarabine, azacytidine, azacytidine-5, 5-azacytidine	antineoplastic	17552	9583	-22.8	0.0
flumethasone pivalate, flumethazone pivalate	glucocorticoid	15427	5699	-22.8	0.0
biperiden, biperiden hydrochloride	antiparkinsonian	16478	6651	-22.8	0.0
atracurium, atracurium besylate	anticonvulsant	16480	6653	-22.9	0.0
clozapine, clozapine-n-oxide	antipsychotic	17189	9113	-22.9	0.0
dibucaine, dibucaine hydrochloride	anesthetic	13724	5246	-23.0	0.0

mesoridazine, mesoridazine besylate	antipsychotic	16490	6663	-23.0	0.0
pergolide, pergolide mesylate	antiparkinsonian	18238	9505	-23.1	0.0
cinoxacin	antibiotic	18247	9514	-23.2	0.0
fluocinolone acetonide, fluocinolone acetonide 21-acetate	antiinflammatory	15454	5726	-23.4	0.0
hemicholinium bromide		17602	9633	-23.4	0.0
perborate monohydrate, perboratemonohydrate, sodium perborate monohydrate, sodium perborate tetrahydrate	antiseptic	16085	7829	-23.5	0.0
fluorometholone acetate	glucocorticoid	15473	5745	-23.8	0.0
meclizine, meclizine hydrochloride	antiemetic	15990	6436	-23.9	0.0
prochlorperazine, prochlorperazine dimaleate, prochlorperazine dimaleate salt	antiemetic	15990	6436	-23.9	0.0
inulin	antiulcerative	15996	6442	-24.0	0.0
bismuth subgallate, bismuth iii gallate basic hydrate	antiseptic, astringent	15997	6443	-24.0	0.0
corticosterone		17668	9699	-24.3	0.0
rugby artificial tears solution (1.4% polyvinyl alcohol)		15371	5598	-24.3	0.0
taurocholic acid, taurocholic acid, sodium salt hydrate	choleretic	16012	6458	-24.3	0.0
benzydamine, benzydamine hydrochloride	analgesic	17302	9226	-24.4	0.0
fenoprofen, fenoprofen calcium salt dihydrate, fenoprofen calcium salt hydrate	antiinflammatory	15516	5788	-24.7	0.0

dimethadione, dimethadione (5,5 dimethyl-2,4oxazolidinedione)	anticonvulsant	17327	9251	-24.8	0.0
silver sulfadiazine 1%, silver sulfadiazine, silver (i) sulfadiazine, 98%	antibacterial	15639	7503	-24.8	0.0
magnesium carbonate	antacid	16043	6489	-24.9	0.0
rubidium, rubidium chloride rb 82, rubidium chloride	antidepressant, diagnostic aid	16590	6763	-24.9	0.0
deoxycholic acid 1.5gr, deoxycholic acid	choleretic	16051	6497	-25.0	0.0
urea	diuretic	17730	9761	-25.1	0.0
calcium carbonate	antacid	16068	6514	-25.4	0.0
glycopyrrolate	antispasmodic	13824	5346	-25.4	0.0
oxybutynin, oxybutynin chloride	urologic	14450	6021	-25.4	0.0
minoxidil	antihypertensive	18419	9686	-25.4	0.0
mesna, mesna (2-mercaptoethane sulfonic acid sodium salt), mesna (2-mercaptoethanesulfonic acid)	mucolytic	14459	6030	-25.6	0.0
anthralin, anthralin (dithranol)	dermatologic	15561	5833	-25.7	0.0
ambroxol, ambroxol hydrochloride	expectorant	19604	11236	-25.7	0.0
lipoic acid, alpha, thioctic acid, lipoic acid, alpha (+-)-, thioctic acid (lipoamide)	misc- hepatic protectant, antidote, hepatic protectant	19627	11259	-25.9	0.0
vigabatrin	anticonvulsant	17413	9337	-25.9	0.0
aminopentamide sulfate, aminopentamide	antispasmodic	13864	5386	-26.3	0.0
thiethylperazine, thiethylperazine malate	antiemetic., antiemetic	16120	6566	-26.4	0.0
memantine, memantine hydrochloride	antiparkinsonian	18494	9761	-26.4	0.0

brimonidine, brimonidine tartrate, brimonidine (uk 14,304)	antiglaucoma	15468	5695	-26.4	0.0
scopolamine methyl bromide	antispasmodic	13871	5393	-26.5	0.0
naproxen, naproxen sodium salt'(r) -, naproxen(+), naproxen (s-6-methoxy methyl 2-naphthalene acetic acid), naproxen sodium, naproxen (+)	antiinflammatory	15601	5873	-26.5	0.0
orphenadrine citrate, orphenadrine	muscle relaxant (skeletal)	13874	5396	-26.5	0.0
procaine, procaine hydrochloride	anesthetic	13885	5407	-26.8	0.0
metoclopramide, metoclopramide hydrochloride, metoclopramide monohydrochloride, metoclopromide	antiemetic	19736	11368	-27.1	0.0
ergocryptine-alpha		17895	9926	-27.2	0.0
mivacurium, mivacurium chloride	muscle relaxant, muscle relaxant (skeletal)	13910	5432	-27.4	0.0
magnesium hydroxide	antacid	16175	6621	-27.4	0.0
phosphatemonobasic, potassium phosphate monobasic, sodium phosphate monobasic	laxative	16177	6623	-27.5	0.0
metergoline	pituitary	17920	9951	-27.5	0.0
flunisolide	glucocorticoid	15650	5922	-27.6	0.0
bismuth iii phosphate, bismuth (iii) phosphate	antacid	16184	6630	-27.6	0.0

tranylcypromine, tranylcypromine sulfate, tranylcypromine hcl (trans-2-phenylcyclopropylamine), trans-phenylcyclopropylamine hydrochloride	antidepressant	16744	6917	-27.7	0.0
perphenazine	antipsychotic	18604	9871	-27.8	0.0
chlorpromazine, chlorpromazine hydrochloride	antiemetic	16199	6645	-27.9	0.0
mandelic acid	dermatologic	17956	9987	-27.9	0.0
roxindole		17565	9489	-28.0	0.0
ethylenediamine, trisodium ethylenediamine tetracetatethylenediamine dihydrochloride	urologic	14575	6146	-28.0	0.0
ethohexadiol, ethohexadiol (2-ethyl-1,3-hexanediol)	insecticide	14576	6147	-28.0	0.0
penicillamine, l-(+)-penicillamine, l-penicillamine, d-penicillamine	antirheumatic	15673	5945	-28.1	0.0
sodium ethasulfatethasulfatethasulfate(2 ethylhexylsulfate), sodium ethasulfate (sodium-2-ethylhexyl sulfate)	misc- surfactant, respiratory stimulant	14579	6150	-28.1	0.0
isobutamben, isobutamben (isobutyl 4-aminobenzoate)	anesthetic	13941	5463	-28.1	0.0
gold au 198, gold, gold sodium thiosulfate, gold(iii) chloride trihydrate	antineoplastic, antirheumatic	16257	7003	-28.2	0.0
acetylleucine, acetyl-l-leucine, n-acetyl-l-leucine	antiemetic	17582	9506	-28.2	0.0
scopolamine n butyl bromide ( ), scopolamine n-butyl bromide (-)	antispasmodic	17590	9514	-28.3	0.0

dioxybenzone, dioxybenzone (2,2'-dihydroxy-4-methoxybenzophenone)	dermatologic	15554	5781	-28.3	0.0
zopiclone	sedative	17606	9530	-28.5	0.0
ferrous fumarate	misc- hematinic	14604	6175	-28.6	0.0
dipyridamole	antithrombotic	16581	8579	-28.7	0.0
benzoin tincture, compound 0.5oz", benzoin, benzoin tincture, bitter tincture	antiseptic, therapeutic plant extract	16422	8166	-28.9	0.0
ferrous gluconate hydrate, ferrous lactate	misc- hematinic	14620	6191	-28.9	0.0
aprepitant	antiemetic., antiemetic	16255	6701	-29.0	0.0
alverine, alverine citrate	antispasmodic	18694	9961	-29.0	0.0
ammonium phosphate dibasic	antiseptic	16439	8183	-29.1	0.0
boric-acid, boric acid	antiseptic	16267	6713	-29.2	0.0
diphenidol, diphenidol hydrochloride, difenidol hydrochloride (diphenidol)	antiemetic	16272	6718	-29.3	0.0
trimethadione, trimethadione (3,5,5,trimethyloxazol-idine,2,4 dione)	anticonvulsant	16837	7010	-29.4	0.0
trihexyphenidyl, trihexyphenidyl hydrochloride, trihexyphenidyl-d,l hydrochloride	antiparkinsonian	18732	9999	-29.5	0.0
phenylephrine, phenylephrine hydrochloride, l-phenylephrine hydrochloride	mydriatic	15608	5835	-29.5	0.0
oxyphencyclimine, oxyphencyclimine hydrochloride	antispasmodic	14023	5545	-30.0	0.0

trazodone, trazodone hydrochloride	antidepressant	18774	10041	-30.0	0.0
gaboxadol, thip hydrochloride, gaboxadol hydrochloride	anticonvulsant	18136	10167	-30.3	0.0
ferrous sulfate, iron sulfate 0.194gm, iron sulfate, iron(ii) sulfate heptahydrate	hematinic	14684	6255	-30.3	0.0
zinc oxide	astringent	16324	6770	-30.3	0.0
oxitriptan, hydroxydltryptophan, l-5-hydroxytryptophan, 5-hydroxy-dl-tryptophan	anticonvulsant, antidepressant	17759	9683	-30.6	0.0
isoproterenol (+)-bitartrate salt'(+)-		18165	10196	-30.6	0.0
tridihexethyl, tridihexethyl chloride	antispasmodic, mydriatic	14050	5572	-30.7	0.0
caffeine sodium benzoate, caffeine-sodium benzoate	nootropic	15669	5896	-30.9	0.0
benactyzine, benactyzine hcl	antispasmodic	14062	5584	-30.9	0.0
iodobenzoic acid, 2-iodobenzoic acid	unclassified	16558	8302	-31.0	0.0
risocaine, risocaine (n-propyl-p-aminobenzoate)	anesthetic	14066	5588	-31.0	0.0
magnesium oxide	phamaceutic aid	16366	6812	-31.1	0.0
omeprazole esomeprazole, omeprazole	antiulcerative	16383	6829	-31.4	0.0
sulfamethoxazole	antibiotic	18894	10161	-31.6	0.0
camphor water qs, camphor, camphor (1r), 1s-camphor, camphor, (1r)-(+)-, camphor white oil	analgesic, therapeutic plant extract	14090	5612	-31.6	0.0
chloroprocaine, chloroprocaine hydrochloride	anesthetic	14093	5615	-31.7	0.0

lithium carbonate	sedative	15706	5933	-31.7	0.0
simethicone, simethicone (40mg/0.6ml)	antiflatulent	14754	6325	-31.7	0.0
frovatriptan	antimigraine	15720	5947	-32.0	0.0
mesna, mesna (2-mercaptoethane sulfonic acid sodium salt), mesna (2-mercaptoethanesulfonic acid)	mucolytic	14768	6339	-32.0	0.0
aluminum chloride, aluminum chloride hexahydrate	dermatologic	15860	6132	-32.1	0.0
choline dihydrogen citrate, ferrocholinate	nootropic, hematinic	15725	5952	-32.1	0.0
zomepirac, zomepirac sodium, zomepirac sodium salt	analgesic	14115	5637	-32.2	0.0
proglumide	antiulcerative	18319	10350	-32.6	0.0
danthron, danthron (1,8-dihydroxyanthraquinone)	laxative	16446	6892	-32.6	0.0
diphenoxylate, diphenoxylate /atropine sulfate, diphenoxylate hydrochloride, diphenoxylate hydrochloride/atropine sulfate	antispasmodic	14135	5657	-32.7	0.0
cevimeline, cevimeline hydrochloride	misc- sialagogue., gastroprokinetic	14801	6372	-32.7	0.0
valethamate bromide	antispasmodic, mydriatic	14138	5660	-32.7	0.0
citrate monohydrate, citric acid, citric acid trisodium salt dihydrate, potassium citrate monohydrate	anticoagulant, misc- antiurolithic	14802	6373	-32.7	0.0
lithium, lithium hydroxide	antipsychotic	17922	9846	-32.8	0.0
pepstatin, pepstatin a	unclassified	18349	10380	-33.0	0.0

fluorescein, fluorescein sodium	diagnostic aid	17000	9536	-33.1	0.0
minaprine, minaprine dihydrochloride, minaprine hydrochloride	antidepressant	17964	9888	-33.4	0.0
butylparaben, butyl p hydroxybenzoate, butyl paraben (butyl 4-hydroxybenzoate)	phamaceutic aid	14838	6409	-33.5	0.0
amethopterin (r,s)		18394	10425	-33.6	0.0
panthenol, dexpanthenol, panthenol (d), dexpanthenol (d-panthenol), panthenol (pantothenol)	cholinergic	15793	6020	-33.6	0.0
streptozocin, streptozotocin, streptozosin	antineoplastic	16560	7306	-33.7	0.0
meticrane	diuretic	18411	10442	-33.8	0.0
magnesium bromide	sedative	15810	6037	-34.0	0.0
carbamazepine	anticonvulsant	19087	10354	-34.1	0.0
pimozide	antipsychotic	18443	10474	-34.2	0.0
cevimeline, cevimeline hydrochloride	misc- sialagogue., gastroprokinetic	14878	6449	-34.3	0.0
valproic acid, valproic acid (2-propylpentanoic acid)	anticonvulsant	17112	7285	-34.5	0.0
octyl methoxycinnamate 7.5%, octinoxate, octyl methoxycinnamate, octyl methoxycinnamate (2-ethylhexyl trans-4-methoxycinnamate)	dermatologic	15852	6079	-35.0	0.0
meperidine, meperidine hydrochloride, meperidine hcl	analgesic	14236	5758	-35.0	0.0

loxapine, loxapine succinate	anxiolytic	19162	10429	-35.1	0.0
laureth 9, polidocanol	anesthetic, phamaceutic aid	18094	10018	-35.1	0.0
anisotropine, anisotropine methylbromide	antispasmodic	14240	5762	-35.1	0.0
triclocarban, trilocarban, trilocarban (3,4,4'-trichlorocarbanilide, 99%)	antiseptic	17405	9762	-35.2	0.0
diphemanil methylsulfate	dermatologic	16006	6278	-35.2	0.0
bismuth subcarbonate, bismuth subcarbonate (bismuth carbonate oxide)	antacid	16585	7031	-35.3	0.0
acedoben, acedoben (4-acetamidobenzoic acid)	nootropic	18109	10033	-35.3	0.0
ketorolac, ketorolactromethamine, ketorolac tromethamine	analgesic	14255	5777	-35.5	0.0
salicyl alcohol, salicyl alcohol (2-hydroxybenzyl alcohol)	anesthetic	14259	5781	-35.6	0.0
naphazoline, naphazoline hydrochloride	decongestant	19206	10473	-35.6	0.0
nabumetone	antiinflammatory	16026	6298	-35.7	0.0
tribromoethanol, tribromoethanol (2,2,2-tribromoethanol)	anesthetic	18143	10067	-35.8	0.0
morantel tartrate, morantel citrate	anthelminithic	18570	10601	-35.8	0.0
aluminum sulfate	antiseptic	16630	7076	-36.2	0.0
xylitol, adonitol (ribitol)	pharmaceutic aid, phamaceutic aid	16963	9332	-36.3	0.0

hyoscyamine, atropine, belladonna alkaloids, belladonna alkaloids 0.016gm/100ml, hyoscyamine (l), atropine sulfate monohydrate, atropine sulfate, l-hyoscyamine hydrochloride, belladonna tincture	mydriatic, therapeutic plant extract, antispasmodic	14290	5812	-36.3	0.0
quetiapine, quetiapine fumarate	antipsychotic	17208	7381	-36.3	0.0
phenazopyridine, phenazopyridine hydrochloride	analgesic	14292	5814	-36.3	0.0
boldine		18612	10643	-36.4	0.0
norethindrone	progestogen	18613	10644	-36.4	0.0
chlorphenesin carbamate, chlorphensin carbamate	muscle relaxant (skeletal)	14322	5844	-37.0	0.0
dehydrocholic acid	choleretic	16677	7123	-37.1	0.0
scopolamine, scopolamine hydrobromide, scopolamine hydrochloride, scopolamine (-)	antispasmodic	14324	5846	-37.1	0.0
dimenhydrinate	antiemetic	16682	7128	-37.2	0.0
pilocarpine, pilocarpine nitrate, pilocarpine hydrochloride	antiglaucoma	15956	6183	-37.3	0.0
gallamine triethiodide	neuromuscular blocking agent	14332	5854	-37.3	0.0
meclofenoxate, meclofenoxate hydrochloride, meclofenoxate (centrophenoxine hydrochloride)	nootropic	18260	10184	-37.4	0.0

ondansetron, ondansetron hydrochloride, ondansetron hcl	antiemetic	16700	7146	-37.5	0.0
ethyl vinyl ether 97%, ethyl vinyl ether	anesthetic	14348	5870	-37.7	0.0
phenacaine, phenacaine hydrochloride	anesthetic, analgesic	14367	5889	-38.1	0.0
methantheline, methantheline bromide	antispasmodic	14381	5903	-38.4	0.0
chlorobutanol	analgesic	14382	5904	-38.4	0.0
isometheptene, isometheptene mucate	antimigraine	16021	6248	-38.7	0.0
bismuth subsalicylate, bismuth subsalicylate 262mg/15ml	antidiarrheal	15092	6663	-38.8	0.0
promazine, promazine hydrochloride	antipsychotic	19465	10732	-39.0	0.0
butamben, butamben (n-butyl p aminobenzoate)	anesthetic	14405	5927	-39.0	0.0
difenoxin	antispasmodic, antidiarrheal	14406	5928	-39.0	0.0
scopolamine n-oxide 0.25mg, scopolamine n oxide, scopolamin-n-oxide hydrobromide	antispasmodic	18835	10866	-39.2	0.0
pyritinol	nootropic	18399	10323	-39.2	0.0
pyrantel tartrate	anthelminithic	18839	10870	-39.3	0.0
ornidazole	antibacterial	18865	10896	-39.6	0.0
orotic acid	uricosuric	15131	6702	-39.6	0.0
dimethyl phthalate	vitamin	15133	6704	-39.6	0.0
picrotoxin, picrotin	nootropic	16073	6300	-39.9	0.0
ferric pyrophosphate	misc- hematinic	15146	6717	-39.9	0.0
oxytocin	oxytocic	15148	6719	-39.9	0.0
methocarbamol, methocarbamol (guaiacol glyceryl ether carbamate)	muscle relaxant, muscle relaxant (skeletal)	14454	5976	-40.1	0.0
benzyl salicylate 0.7%, benzyl salicylate	antiulcerative	16841	7287	-40.2	0.0

trimipramine, trimipramine maleate, trimipramine maleate salt	antidepressant	19566	10833	-40.3	0.0
quinaldine	anesthetic	14467	5989	-40.4	0.0
decamethonium, decamethonium bromide	muscle relaxant (skeletal)	14467	5989	-40.4	0.0
acetal 0.10gm, acetal	sedative	16112	6339	-40.7	0.0
benzyl alcohol	antiseptic	16869	7315	-40.8	0.0
flavoxate, flavoxate hydrochloride	antispasmodic	14516	6038	-41.6	0.0
succinylcholine, succinylcholine chloride, succinylcholine chloride dihydrate	neuromuscular blocking agent., neuromuscular blocking agent	14519	6041	-41.7	0.0
tetrandrine	analgesic	18615	10539	-42.1	0.0
mepenzolate, mepenzolate bromide	antispasmodic	14545	6067	-42.3	0.0
witch hazel		16978	7424	-42.9	0.0
lidocaine, lidoca•ne hydrochloride, lidocaine hydrochloride	anesthetic	14590	6112	-43.3	0.0
chloroform	anesthetic	14594	6116	-43.4	0.0
dibenzepin, dibenzepin hydrochloride, dibenzepine	antidepressant	18723	10647	-43.6	0.0
bitartrate	laxative	17017	7463	-43.6	0.0
dichlorphenamide, dichlorophenamide	antiglaucoma	16249	6476	-43.8	0.0
cortisone		19213	11244	-44.1	0.0
ammonium salicylate	analgesic	14646	6168	-44.6	0.0
dipyrone	analgesic	14647	6169	-44.7	0.0
eugenol, zinc eugenol, zinc-eugenol	analgesic, therapeutic plant extract	14648	6170	-44.7	0.0
cholic acid 33/4gr, cholic acid, cholic acid (3 alpha, 7 alpha, 12 alpha trihydroxy 5-beta cholanolic acid)	choleretic	17081	7527	-44.9	0.0
phenacetin, phenacetin (acetophenetidin)	analgesic	14660	6182	-45.0	0.0

benzocainethyl 3 aminobenzoate, benzocaine (ethyl p-aminobenzoate), ethyl 3-aminobenzoate (3-aminobenzoic acid ethyl ester)	anesthetic, n/a	14668	6190	-45.2	0.0
sodium bicarbonate, sodium bicarbonate, powder	antacid	17097	7543	-45.2	0.0
nikethamide, nikethamide (n,n diethylnicotinamide)	nootropic	16316	6543	-45.3	0.0
gluconic acid 1%, ferrous gluconate, gluconic acid, ferrous gluconate hydrate, d-gluconic acid	pharmaceutic aid, misc- hematinic	15406	6977	-45.3	0.0
rutin, rutoside (rutin), rutoside (rutin)	capillary protectant	15427	6998	-45.8	0.0
rocuronium, rocuronium bromide, rocuronium bromide (zemuron)	neuromuscular blocking agent	14715	6237	-46.3	0.0
bemegride, bemegride (3-methyl-3-ethylglutarimide)	nootropic	16364	6591	-46.3	0.0
methylatropine nitrate, methylatropine nitrate (atropine methyl nitrate)	mydriatic	16374	6601	-46.5	0.0
meparfynol, meparfynol (methylpentynol, 3-methyl-1-pentyn-3-ol)	sedative	16391	6618	-46.9	0.0
eletriptan, eletriptan hydrobromide	antimigraine., antimigraine	16398	6625	-47.1	0.0
guanfacine, guanfacine hydrochloride, guanidine hydrochloride	antihypertensive	16438	6665	-48.0	0.0
almotriptan	antimigraine	16439	6666	-48.0	0.0
calamine		17249	7695	-48.1	0.0

pancuronium, pancuronium bromide	neuromuscular blocking agent	14797	6319	-48.2	0.0
sodium bismuthate 150mg, sodium bismuthate	antacid	17278	7724	-48.6	0.0
pipenzolate bromide	antispasmodic	14817	6339	-48.7	0.0
neostigmine methylsulfate, neostigmine methyl sulfate	cholinergic	16470	6697	-48.7	0.0
1,2-ethanedisulfonic acid potency not given, 1,2-ethanedisulfonic acid	sedative	16489	6716	-49.1	0.0
ethynodiol diacetate	anesthetic	14839	6361	-49.2	0.0
isopropamide, isopropamide iodide	antispasmodic	14857	6379	-49.6	0.0
cimetidine	antiulcerative	17331	7777	-49.7	0.0
racemorphan, levorphanol, dextrorphan, dextrorphan hydrochloride, levorphanol tartrate	analgesic	19188	11112	-49.9	0.0
sulisobenzone, sulisobenzone (2-hydroxy-4-methoxy-5-sulfobenzophenone)	dermatologic	16540	6767	-50.2	0.0
busulphan		19724	11755	-50.6	0.0
strychnine	nootropic	16616	6843	-51.9	0.0
diethyl oxalate 0.0324gm, diethyl oxalate	anorexic	15791	7362	-53.3	0.0
acecarbromal, acecarbromal (acetylcarbromal)	sedative	16683	6910	-53.4	0.0
methylene blue, methylene blue hydrate	hemantic	15802	7373	-53.6	0.0
proparacaine, proparacaine hydrochloride	anesthetic	15032	6554	-53.7	0.0
tropicamide	mydriatic, opthalmic	16706	6933	-53.9	0.0

levobunolol, bunolol, bunolol hydrochloride, levobunolol hydrochloride, levobunolol hydrochloride'(-) -, levobunolol hydrochloride'(+) -, levobunolol, hcl (-)	antiglaucomantihy pertensive	16806	7033	-56.1	0.0
menthyl salicylate 5.000%, menthyl salicylate	dermatologic	16875	7102	-57.7	0.0
ferric ammonium citrate	hematinic	16009	7580	-57.9	0.0
hyoscyamine, atropine, belladonna alkaloids, belladonna alkaloids 0.016gm/100ml, hyoscyamine (l), atropine sulfate monohydrate, atropine sulfate, l-hyoscyamine hydrochloride, belladonna tincture	mydriatic, therapeutic plant extract, antispasmodic	16898	7125	-58.2	0.0
sulfinpyrazone, sulfinpyrazone (+-)	antiurolithic	16066	7637	-59.1	0.0
edrophonium, edrophonium chloride	cholinergic	16945	7172	-59.2	0.0
methacholine, methacholine chloride	cholinergic	16946	7173	-59.2	0.0
ergotaminergotamine tartratergonovine maleate	analgesic	16947	7174	-59.3	0.0
2,2,2-trichloroethanol unk, 2,2,2-trichloroethanol	sedative	16988	7215	-60.2	0.0
donepezil, donepezil hydrochloride	nootropic	17022	7249	-60.9	0.0
neostigmine bromide, neostigmine bromine	cholinergic	17029	7256	-61.1	0.0
brilliant blue	n/a	18311	9129	-62.4	0.0
sodium succinate 30%, succinate, sodium succinate dibasic hexahydrate	respiratory stimulant	16247	7818	-62.8	0.0

diperodon, diperodon hydrochloride	anesthetic	15530	7052	-65.4	0.0
methylatropine nitrate, methylatropine nitrate (atropine methyl nitrate)	mydriatic	17294	7521	-67.0	0.0
acetohydroxamic-acid, acetohydroxamic acid	misc- antiurolithic, antiurolithic	16461	8032	-67.3	0.0
panthenol, dexpanthenol, panthenol (d), dexpanthenol (d-panthenol), panthenol (pantothenol)	cholinergic	17511	7738	-71.8	0.0
artificial tears		17591	7818	-73.6	0.0
mifepristone	abortifacient	16968	8539	-77.9	0.0
sibutramine, sibutramine hydrochloride, sibutramine hcl	antiobesity	17012	8583	-78.8	0.0
finasteride, propecia	urologic	17259	8830	-83.9	0.0
colloidal oatmeal (aveeno)		24104	14376	-209.7	0.0
