# Supplementary material for: Gametocytocidal Screen Identifies Novel Chemical Classes with Plasmodium falciparum Transmission Blocking Activity
Source: PLoS One. 2014 Aug 26;9(8):e105817. doi: 10.1371/journal.pone.0105817 (PMC4144897; doi:10.1371/journal.pone.0105817)
Supplement: Table S7 — Membrane feeding assay data. (PDF) [file pone.0105817.s007.pdf]

Table S7. Membrane Feeding Assay Data

	Control	0.5 um Clotrimazole	5 uM Clotrimazole	0.5 uM Pyr Pam	5 uM Pyr Pam		Control	Methylene blue 0.5 uM	Methylene Blue 5uM	Cetalkonium 0.5uM	Cetalkonium 5 uM
Number of values	34	40	41	38	33		38	39	38	33	35
Minimum	17	4	1	0	0		4	0	0	0	0
25% Percentile	42.5	32.25	19	0	0		50.5	0	0	33	0
Median	53	41.5	26	0	0		58.5	0	0	41	0
75% Percentile	62.75	53.75	37	0	0		76.5	0	0	55	0
Maximum	101	84	66	0	0		132	0	0	78	2
Mean	54.41	42.15	28.95	0	0		61.45	0	0	41.42	0.2571
Std. Deviation	19.03	17.87	13.71	0	0		23.71	0	0	16.88	0.5606
Std. Error	3.263	2.825	2.141	0	0		3.846	0	0	2.938	0.09476
Lower 95% CI of mean	47.77	36.44	24.62	0	0		53.65	0	0	35.44	0.06456
Upper 95% CI of mean	61.05	47.86	33.28	0	0		69.24	0	0	47.41	0.4497
Sum	1850	1686	1187	0	0		2335	0	0	1367	9
